# Rapid molecular assays versus blood culture for bloodstream infections: a systematic review and meta-analysis

**DOI:** 10.1016/j.eclinm.2024.103028

**Published:** 2025-01-10

**Authors:** Gabriella Anna Rapszky, Uyen Nguyen Do To, Veronika Eszter Kiss, Tamás Kói, Anna Walter, Dorottya Gergő, Fanni Adél Meznerics, Márton Rakovics, Szilárd Váncsa, Lajos Vince Kemény, Dezső Csupor, Péter Hegyi, Michael R. Filbin, Csaba Varga, Bánk G. Fenyves

**Affiliations:** a Department of Emergency Medicine, Semmelweis University, Budapest, Hungary; b Centre for Translational Medicine, Semmelweis University, Budapest, Hungary; c András Pető Faculty, Semmelweis University, Budapest, Hungary; d Budapest University of Technology and Economics, Department of Stochastics, Budapest, Hungary; e Institute for Translational Medicine, Medical School, University of Pécs, Pécs, Hungary; f Department of Pharmacognosy, Semmelweis University, Budapest, Hungary; g Department of Dermatology, Venereology and Dermatooncology, Semmelweis University, Budapest, Hungary; h Eötvös Loránd University, Faculty of Social Sciences, Department of Statistics, Budapest, Hungary; i Institute of Pancreatic Diseases, Semmelweis University, Budapest, Hungary; j Department of Physiology, Semmelweis University, Budapest, Hungary; k HCEMM-SU, Translational Dermatology Research Group, Semmelweis University, Budapest, Hungary; l Institute of Clinical Pharmacy, University of Szeged, Szeged, Hungary; m Department of Emergency Medicine, Massachusetts General Hospital and Harvard Medical School, Boston, MA, USA; n Department of Molecular Biology, Semmelweis University, Budapest, Hungary

**Keywords:** Rapid pathogen identification, Whole blood, Suspected sepsis, Diagnostic method, Blood culture, Diagnostic accuracy

## Abstract

**Background:**

Timely management of sepsis with early targeted antimicrobial therapy improves patient outcomes. Rapid molecular assays (RMAs) have emerged, enabling the detection of bloodstream infection (BSI) with a shorter turnaround time than blood cultures (BCs). The accuracy of several RMAs has not been comprehensively reviewed. We aimed to identify commercial RMAs reported in the literature and evaluate their diagnostic performance compared to BC.

**Methods:**

A systematic review and meta-analysis was conducted, covering MEDLINE, Cochrane Library, Embase, and Web of Science from inception to September 23, 2024. Eligible studies included patients with suspected or documented BSI, tested with both an RMA (turnaround time of ≤12 h, targeting ≥20 pathogens) and BC. Non-original research articles and animal studies were excluded. The primary outcomes were pooled sensitivity and specificity of RMAs for pathogen detection compared to BC. Bivariate analysis was used to produce summary receiver operating characteristic plots and diagnostic metric measures stratified by different units of analysis (sample versus patient), RMA types, and patient populations. Risk of bias was assessed using the Quality Assessment of Diagnostic Accuracy Studies-2 (QUADAS-2) and Quality Assessment of Diagnostic Accuracy Studies-Comparative (QUADAS-C) tools. The study was registered with PROSPERO, CRD42022377280.

**Findings:**

A total of 63,916 articles were identified, of which 104 were included in the qualitative synthesis and 75 in the quantitative synthesis, covering 17,952 samples and 11,393 patients analyzed separately. Eleven RMAs were identified, with four included in the RMA-based subgroup analysis (LightCycler SeptiFast Test MGRADE®, IRIDICA BAC BSI assay, SepsiTest, MagicPlex Sepsis Test) and five additional ones in the pooled analysis (UMD-SelectNA, VYOO®, MicrobScan assay, MicrobScan-Kairos24/7, REBA Sepsis-ID test). Two RMAs were included in the qualitative synthesis only (InfectID-BSI, Pilot Gene Technology droplet digital polymerase chain reaction). Pooled specificity of RMAs was higher (0.858, 95% confidence interval (CI) 0.830–0.883) than sensitivity (0.659, 95% CI 0.594–0.719) by patient. Sensitivities varied by RMA type from 0.492 (95% CI 0.390–0.594, MagicPlex Sepsis Test) to 0.783 (95% CI 0.662–0.870, IRIDICA BAC BSI assay) by patient. Specificities varied more by patient population, ranging from 0.811 (95% CI 0.716–0.879) in the intensive care population to 0.892 (95% CI 0.838–0.930) in the emergency department population, by patient. Similar metrics were observed when the analysis was done by sample. Risk of bias was judged to be high in all included articles.

**Interpretation:**

Despite their shorter turnaround time, low sensitivity means RMAs cannot replace BCs. However, our data indicate that RMAs may have value as an add-on test by increasing pathogen detection rates. Higher-sensitivity RMAs are needed which could possibly be achieved by expanding pathogen coverage and increasing blood sample volumes. High-quality implementation studies and standardized reporting are required to assess the clinical advantages of RMAs.

**Funding:**

Centre for Translational Medicine, 10.13039/501100002332Semmelweis University.

**Research in context**

**Evidence before this study**

Timely management of sepsis improves patient outcomes, but targeted antimicrobial therapy relies on pathogen identification. Blood cultures, the current gold standard, have a long turnaround time. Rapid molecular assays (RMAs) have been developed to address this issue, but most clinical guidelines do not recommend replacing blood cultures with RMAs due to a lack of validation and limited clinical experience. Previous meta-analyses reviewed commercial RMAs with a target panel of at least 20 pathogens and a maximum 12-h turnaround time. Despite their short turnaround time, these studies did not recommend replacing blood cultures with RMAs due to their low sensitivity. Only one 2013 meta-analysis suggested RMA-guided antimicrobial management, however, that study used various reference tests alongside the blood culture. Limitations of these studies include the narrow scope of RMAs evaluated and the potential bias in diagnostic metrics due to pooling different units of analysis. As a result, many RMAs with published data have not been systematically evaluated.

**Added value of this study**

To our knowledge, this is the first comprehensive systematic review and meta-analysis seeking to include all commercially available RMAs without pre-selecting manufacturers. We identified 11 commercial polymerase chain reaction-based assays, including the previously unassessed MagicPlex Sepsis Test. Pooled sensitivities and specificities of RMAs for detecting bloodstream pathogens were low compared to standard blood cultures, with no significant differences when analyzing patient versus sample data separately. Sensitivities varied more than specificities among assays, with the IRIDICA BAC BSI assay showing the highest sensitivity. Subgroup analysis revealed the highest pooled specificity in emergency department patients and the lowest in intensive care patients. Supplementary analysis indicated that increasing the number of blood culture sets does not change the diagnostic metrics of either the blood culture or the LightCycler SeptiFast MGRADE®.

**Implications of all the available evidence**

Our findings confirm that RMAs still lack the sensitivity to be recommended as a replacement for blood culture for detecting bloodstream infection pathogens. However, diagnostic accuracies presented should be interpreted as a measure of RMAs’ performance against blood culture rather than true pathogen detection capabilities. Our findings suggest that RMAs might have an added value when utilized alongside blood cultures, regardless of the number of blood culture sets drawn. This study does not provide evidence to change current guidelines regarding the utilization of RMAs. High-quality implementation studies are needed to better determine the value of RMAs in clinical practice. For assay development, improved sensitivity might be achieved with wider pathogen coverage and larger blood sample volumes.

## Introduction

Sepsis is defined as life-threatening organ dysfunction caused by a dysregulated host response to infection. If not recognized and treated early, it can lead to septic shock, multiorgan failure, and death.^1^ The global burden of sepsis is increasing in terms of morbidity, mortality, and financial costs, with 48.9 million cases and 11 million deaths annually.^2^^,^^3^ Early appropriate antimicrobial therapy (AMT) is the cornerstone of management and is associated with improved patient outcomes.^4^ Hourly delays in the initiation of appropriate AMT decrease survival in septic shock.^5^ However, ineffective or unnecessarily broad-spectrum empiric AMT is associated with several problems, including extended hospital stay and increased healthcare costs.^6^

Blood culture (BC) is the current gold standard method for identifying bloodstream infections (BSI), but it has limitations, including its long turnaround time (TAT) and lower detection rates in patients on empiric AMT.^7^ Moreover, slow-growing, fastidious organisms are often difficult to detect, and this, alongside AMT, decreases BC’s sensitivity. These factors can delay the initiation of targeted AMT.^8^ Due to the long TAT and the urgency of AMT, empiric broad-spectrum antibiotics (ABs) are often initiated. This presents the risk of inappropriate antimicrobial treatment, which is associated with worse clinical outcomes, increased antimicrobial resistance, and higher financial costs.^9^, ^10^, ^11^ Thus, there is an unmet need for rapid pathogen diagnostics, which would allow clinicians to adjust targeted AMT promptly.

In the last two decades, rapid molecular methods have emerged as alternatives to BC for detecting BSI, including polymerase chain reaction (PCR),^12^^,^^13^ fluorescence in situ hybridization,^14^ biosensors,^15^^,^^16^ and combined methods.^15^^,^^16^ Of these, PCR-based methods are considered the most reliable, due to their high sensitivity and specificity.^17^ Methods vary between testing blood from positive BC bottles or whole blood drawn directly from patients, however, the ideal TAT of a few hours can only be achieved by methods that assay whole blood.^18^^,^^19^ Several whole-blood-based rapid molecular assays (RMAs) with shorter TAT^20^ were shown to contribute to decreased hospital or intensive care unit (ICU) stay.^21^ Despite promising results, however, guidelines do not recommend the replacement of BC with non-culture-based RMAs for the diagnosis of BSI due to limited clinical evidence and uncertain diagnostic accuracy.^19^ Moreover, replacing BCs with RMAs would limit our ability to store and sequence pathogens, track epidemiological trends in virulence and resistance, map transmission routes, and create biobanks that support vaccine development. Prior meta-analyses have investigated the diagnostic accuracy of RMAs, however, these meta-analyses are subject to limitations regarding the comparison, study selection, or narrow scope of RMAs analyzed.^22^, ^23^, ^24^, ^25^, ^26^

To address previous meta-analyses’ limitations and expand the coverage of studies, a comprehensive systematic review and meta-analysis was conducted to evaluate the diagnostic performance of commercially available RMAs for rapid pathogen detection of BSI in whole blood, using BC as the reference standard.

## Methods

### Search strategy and selection criteria

A systematic review and meta-analysis was reported according to the Preferred Reporting Items for Systematic Review and Meta-Analysis (PRISMA) statement,^27^ and performed following the recommendations of the Cochrane Handbook.^28^ The review protocol was registered on PROSPERO (registration number CRD42022377280, www.crd.york.ac.uk/prospero).

The systematic literature search was performed in four databases: MEDLINE (via PubMed), Cochrane Library (CENTRAL), Embase, and Web of Science from inception to September 23, 2024. The search query is available in the Supplementary Methods. The search applied no filters. The reference lists of all studies selected for inclusion were also searched.

The PIRD framework guided the formulation of the research question. Original studies were included where (P) patients with suspected sepsis or documented BSI were tested with an (I) index test and the (R) reference BC. The index test was defined as a commercially available (present or past) RMA, processing whole blood, with a target panel of ≥20 bacteria or fungi, at least at the genus level, and with a maximum TAT of 12 h. The reference test was defined as the gold standard BC technique, including at least two bottles (aerobic and anaerobic). Diagnoses of interest (D) were bacteremia or fungemia. Sensitivity (Se) and specificity (Sp) were considered as diagnostic outcomes.

Non-original research articles (reviews, meta-analyses, editorials, letters, comments, notes, short reports, communications, correspondences, case reports/series, protocols, book sections) were excluded along with non-peer-reviewed articles (gray literature). *In vitro* studies, animal studies, non-English reports, and those with sample sizes less than 11 (considered as case series, as suggested by Abu-Zidan et al.)^29^ were excluded. Studies were eligible for data synthesis if they provided data for true positives (TP), false negatives (FN), false positives (FP), and true negatives (TN). Articles, in which samples judged as (possible) contaminants were excluded, or reported the possibility of performing only one bottle of the BC set (either aerobic or anaerobic) were included in the systematic review but were ineligible for the statistical analysis. Publications not reporting on how contaminants were handled were included in the analysis. In the case of multiple publications from the same cohort, or overlapping cohorts, the study with the larger sample size (or, if it was the same, the study focusing on diagnostic accuracy) was selected. Studies reporting performance related to samples or patients were included, while those reporting performance related to episodes were excluded from the analysis due to the heterogeneous episode definitions. However, articles considering one sample/patient per episode were included in the analysis.

After the systematic search, articles were imported into EndNote (EndNote X7.4, Clarivate Analytics, Philadelphia, PA, USA), and duplicates were removed. Remaining articles were divided into 3 pools, each was screened by title-abstract and full text by two independent investigators (GAR and MR, GAR and UNDT, GAR and UNDT) using Rayyan.ai (Rayyan Systems, Inc., Cambridge MA, USA). Disagreements were resolved by reconciliation between the two investigators.

### Data extraction

The following data were extracted: first author, year of publication, country, study design, setting, number, sex, and age of enrolled patients, TP, TN, FP, and FN values (calculated from other parameters (e.g., patient number, sensitivity, specificity, positive predictive value), when necessary). The terms “women” and “female,” as reported by the authors of included studies, were categorized as “female,” and “men” and “male” were categorized as “male” in this report. Where studies provided TP, TN, FP, and FN values for specific subgroups, these data were extracted separately. Information on sampling indications, the number of BC sets drawn, RMA type, and manufacturer sponsorship was also collected. In included studies TP, TN, FP, and FN values were reported related either to samples or patients, which distinct measurement units were classified as “units of analysis”, according to Stevenson et al.^26^ If articles reported contaminants, they were treated as “positives” for the statistical analysis to objectively evaluate assay performance, independent of clinical evaluation. In the case of multiple pathogens, the result was considered positive both for BC and the RMA. Data were collected in a purpose-designed spreadsheet (Office 365 Excel, Microsoft, Redmond, WA, USA) by two independent investigators (GAR and UNDT).

### Statistics

Statistical analyses were carried out using the meta 6.2–1 and lme4 1.1–32 packages of the R statistical software (version 4.1.2.), and the online tools described by Freeman^30^ and Cerullo.^31^ The bivariate model of Reitsma et al.^32^; Chu and Cole^33^ was fitted to pool sensitivity and specificity. For all statistical analyses, a p-value of ≤0.05 was considered significant. Diagnostic performance measures were analyzed separately by sample and patient units because merging these units would be inappropriate from a statistical viewpoint. The summary estimates of sensitivity, specificity, and the corresponding 95% confidence and prediction regions were plotted on a receiver operating characteristic plot. Heterogeneity was evaluated by conducting separate univariate analyses of sensitivity and specificity using the generalized mixed-effect approach of Stijnen^34^ and calculating the *I*^2^ measure.

Subgroup analyses were performed based on 1) RMA types, 2) patient age category (adult versus infant–newborn in applicable RMA subgroups with feasible age comparisons), 3) patient population (emergency department (ED), intensive care unit (ICU), immunosuppressed, mixed: including internal medicine, surgery, and unspecified), 4) sponsorship status (manufacturer-funded versus non-funded), and 5) the number of BC sets (unknown, ≥2 sets, <2 sets). To test the null hypothesis that the sensitivities are equal in two subgroups (the same approach was applied for comparing two specificities), we first calculated the difference between the two logit-transformed sensitivities. Then, we determined the standard error of this difference using independence assumption, i.e., we estimated the standard error by calculating the sum of the variances of the two logit sensitivities and taking the square root of this value. Finally, we generated the p-values using the resulting logit difference and standard error. In a few cases, there were studies contributing to the pooled sensitivities of both compared subgroups. Although each subgroup included different patients, the random effects corresponding to these subgroups are correlated. Thus, in these cases, the independence assumption is only approximately valid. For this reason, as a sensitivity analysis, we also calculated the standard error of the logit difference by assuming a slight correlation between the two compared pooled logit sensitivities. In all cases, we received roughly the same p-value. When several tools/subgroups were compared pairwise, the adjusted p-values suggested by Holm^35^ were also reported to address the problem of multiple comparisons.

Due to the imperfection of the reference BC test which potentially biases diagnostic accuracy measures, a supplementary analysis was performed using the methodology described by Cerullo^31^ and the MetaBayesDTA web application (version 1.5.2) when the number of involved studies was large enough for this complex approach. In summary, this Bayesian approach considers the true disease status of the patients as hidden variables and estimates simultaneously the performance of the analyzed device and the (imperfect) reference standard. In the web application, the imperfect reference standard approach was performed without any assumptions using the default prior distributions by setting the prior mean of the pooled logit sensitivity and specificity to two for both the investigated test and the reference standard. Only articles reporting performance related to patients were considered.

Publication bias was evaluated by performing the methodology of Deeks, Macaskill, and Irwig,^36^ including the modified funnel plot.

### Risk of bias assessment and quality of evidence

The risk of bias (ROB) was assessed for all articles included in the quantitative synthesis using the Quality Assessment of Diagnostic Accuracy Studies-2 (QUADAS-2),^37^ and Quality Assessment of Diagnostic Accuracy Studies-Comparative (QUADAS-C) tools^38^ by two independent authors (GAR and VEK). QUADAS-C was used in cases of more comparable index tests. The study quality characteristics were assessed according to Stevenson et al.,^26^ with modifications detailed in Supplementary Table S1.

Certainty of evidence was assessed based on the Grades of Recommendation, Assessment, Development, and Evaluation (GRADE) workgroup’s recommendation.^39^ GRADE evidence profiles were made with the GRADEpro GDT Software (Copyright 2021, McMaster University and Evidence Prime Inc.) for the investigated outcomes. Disagreements were resolved by reconciliation between the two authors.

### Ethics

Ethical approval was not required for this study since it used only secondary data from published studies.

### Role of funding source

Funding was provided by the Centre for Translational Medicine, Semmelweis University. The funder of the study had no role in the study design, data collection, data analysis, data interpretation, or writing of the report. No authors have been paid to write this article by pharmaceutical companies or other agencies.

## Results

**Fig. 1:**
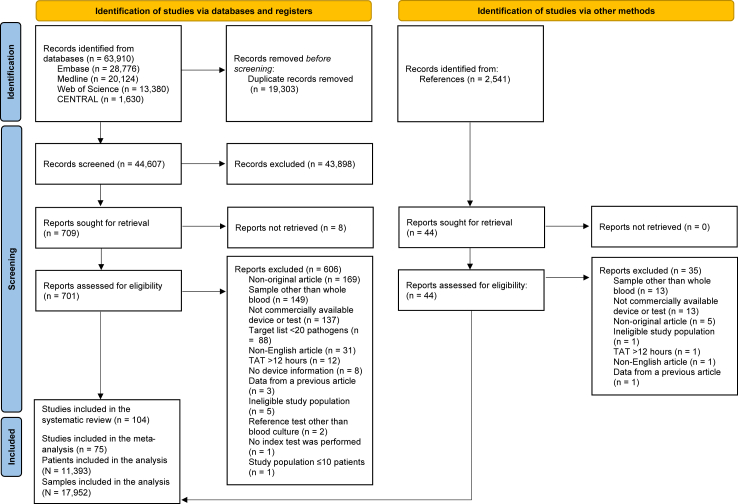
Preferred reporting items for systematic review and meta-analysis (PRISMA) 2020 flowchart representing the study selection process. n = number of publications. N = number of samples or patients. TAT = turnaround time.

A total of 63,916 citations were identified through the database search. After title-abstract (Cohen’s Kappa = 0.8981) and full text (Cohen’s Kappa = 0.8120) selection, 104 articles were included in the qualitative synthesis and 75 in the quantitative synthesis (Supplementary Tables S2 and S3).^12^^,^^13^^,^^20^^,^^40^, ^41^, ^42^, ^43^, ^44^, ^45^, ^46^, ^47^, ^48^, ^49^, ^50^, ^51^, ^52^, ^53^, ^54^, ^55^, ^56^, ^57^, ^58^, ^59^, ^60^, ^61^, ^62^, ^63^, ^64^, ^65^, ^66^, ^67^, ^68^^,^^69^, ^70^, ^71^, ^72^, ^73^, ^74^, ^75^, ^76^, ^77^, ^78^, ^79^, ^80^, ^81^, ^82^, ^83^, ^84^, ^85^, ^86^, ^87^, ^88^, ^89^, ^90^, ^91^, ^92^, ^93^, ^94^, ^95^, ^96^, ^97^, ^98^^,^^99^, ^100^, ^101^, ^102^, ^103^, ^104^, ^105^, ^106^, ^107^, ^108^, ^109^, ^110^, ^111^ Four articles were excluded from the data synthesis due to reporting “episode” as a unit of analysis,^112^, ^113^, ^114^, ^115^ and ten were excluded because the accurate number of excluded contaminants was missing.^116^, ^117^, ^118^, ^119^, ^120^, ^121^, ^122^, ^123^, ^124^, ^125^ Eight studies were only eligible for the qualitative synthesis because data for TP, TN, FP, and FN values were missing.^126^, ^127^, ^128^, ^129^, ^130^, ^131^, ^132^, ^133^ Six articles were excluded due to the possibility of a missing BC bottle type.^134^, ^135^, ^136^, ^137^, ^138^, ^139^ One article was excluded due to a possible 3-day interval between the reference and index test sampling.^140^ The selection process is summarized in a PRISMA flowchart in Fig. 1.

The main characteristics of the included studies for the systematic review and meta-analysis are summarized in Supplementary Tables S2 and S3. The following RMAs met the eligibility criteria (see Methods) for this study: LightCycler SeptiFast Test MGRADE® (Roche Diagnostics, Risch-Rotkreuz, Switzerland) (referred to hereon as Septifast), IRIDICA BAC BSI assay (Abbott Diagnostics, Lake Forest, IL, USA) (referred to hereon as IRIDICA), SepsiTest, UMD-SelectNA (Molzym Molecular Diagnostics, Bremen, Germany), MagicPlex Sepsis Test (Seegene, Seoul, South Korea) (referred to hereon as Magicplex), VYOO® (SIRS-Lab, Jena, Germany), MicrobScan assay, MicrobScan-Kairos24/7 (Nurex, Sassari, Italy), REBA Sepsis-ID test (Optipharm, Osong, Republic of Korea), InfectID-BSI test (Microbio, Queensland, Australia), and the ddPCR (Pilot Gene Technology, Hangzhou, China) (Table 1). A total of 17,952 samples and 11,393 patients from 62 and 43 articles (mostly from high-income countries) were included in the analysis, involving one or more of the aforementioned RMAs.

**Table 1:** Characteristics of rapid molecular assays included in the systematic review and meta-analysis.

	Technology	Coverage of pathogens and resistance genes	TAT(hours)	Recommended blood volume (ml)
LightCycler SeptiFast Test MGRADE®^a^	multiplex real-time PCR	19 bacterial and 6 fungal species (MRSA included - *mecA*)^68^	6–7^97^	1.5^48^
IRIDICA BAC BSI assay^a^^,^^b^	PCR/ESI-MS	>200 different species of bacteria and fungi, *mecA, vanA, vanB, blaKPC*^98^	8^98^	5
SepsiTest	broad-range PCR and sequencing	>200 species from 200 genera of bacteria and 65 genera of fungi^26^^,^^141^	8–12^142^	1^60^
UMD-SelectNA^c^	broad-range PCR and sequencing^143^^,^^144^	>200 species from 200 genera of bacteria and 65 genera of fungi^26^^,^^141^	10^102^	1^69^
MagicPlex Sepsis Test	multiplex real-time PCR	>90 microorganisms at the genus level and 27 at the species level, *mecA, vanA, vanB*^13^	6^13^	1^13^
VYOO®^a^	multiplex PCR and gel electrophoresis	34 bacterial and 6 fungal species, *mecA, vanA, vanB, blaSHV, blaCTX*^53^	8^53^	5^44^
MicrobScan assay	multiplex qPCR	20/40 microorgamisms, *mecA, vanA/vanB, blaKPC*^58^^,^^73^	4–5^58^^,^^73^	0.1^58^
MicrobScan-Kairos24/7^d^^,^^e^	multiplex qPCR	39 microorganisms, *mecA, vanA/vanB, blaKPC*^58^	4^58^	0.1^58^
REBA Sepsis-ID test	PCR-reverse blot hybridization	16 bacterial probes and 5 fungal probes, *mecA, vanA, vanB*^67^	3–4^67^	1^107^
InfectID-BSI test^f^	real-time qPCR	26 microbial targets^133^	3^133^	4^133^
ddPCR assay^f^	5-fluorescent-channel ddPCR system	20 microorganisms, *blaKPC, mecA, blaNDM*|^g^^,^^125^	2.5^125^	5^125^

TAT = turnaround time. PCR = polymerase chain reaction. qPCR = quantitative polymerase chain reaction. ESI-MS = electrospray ionization mass spectrometry. MRSA = Methicillin-resistant *Staphylococcus aureus.* BSI = bloodstream infection. ddPCR = droplet digital polymerase chain reaction.

a Discontinued assay.^142^

b IRIDICA BAC BSI assay was developed from PLEX-ID^26^; solely articles mentioning “IRIDICA” were included.

c Semi-automated version of SepsiTest (automated deoxyribonucleic acid extraction instead of manual) was treated separately.

d Modified version of the MicrobScan assay was treated separately.

e Out of the 40 microorganisms, *Enterobacter cloacae* was excluded from the first version of the MicrobScan assay.

f Included solely in the qualitative synthesis.

g PilotBac-1, PilotBac-2, PilotBac-3, PilotBac-4, PilotFungi, and PilotAMR assay panels were used in the study.

**Fig. 2:**
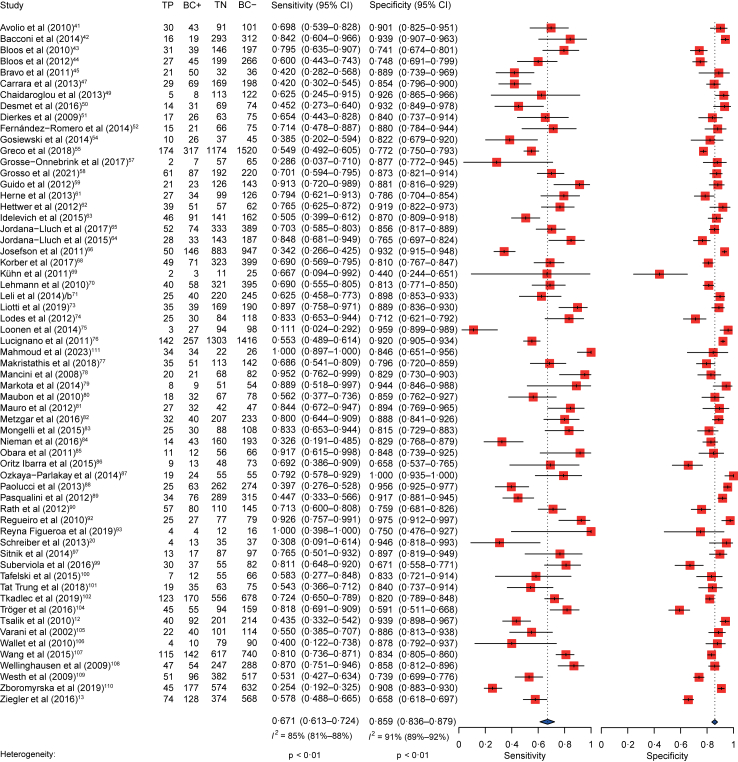
Pooled sensitivity and specificity of rapid molecular assays for the detection of bloodstream pathogens when compared to blood culture. Unit of analysis: sample. TP = true positive. BC+ = blood culture positive. TN = true negative. BC– = blood culture negative. CI = confidence interval.

Estimates of pooled sensitivity and specificity for all nine RMAs included in the statistical analysis compared to BC, by sample were 0.672 (95% confidence interval (CI) 0.615–0.725) and 0.858 (95% CI 0.835–0.879) (Fig. 2), and by patient were 0.659 (95% CI 0.594–0.719) and 0.858 (95% CI 0.830–0.883) (Supplementary Fig. S1). Pooled diagnostic performance of all included RMAs was very similar between sample-versus patient-based analyses (Supplementary Fig. S2). The examined outcomes showed a high between-study heterogeneity for publications reporting performance related both to samples (Se *I*^2^ = 85%, p < 0.01; Sp *I*^2^ = 91%, p < 0.01) and patients (Se *I*^2^ = 78%, p < 0.01; Sp *I*^2^ = 90%, p < 0.01) (Fig. 2, Supplementary Fig. S1).

**Fig. 3:**
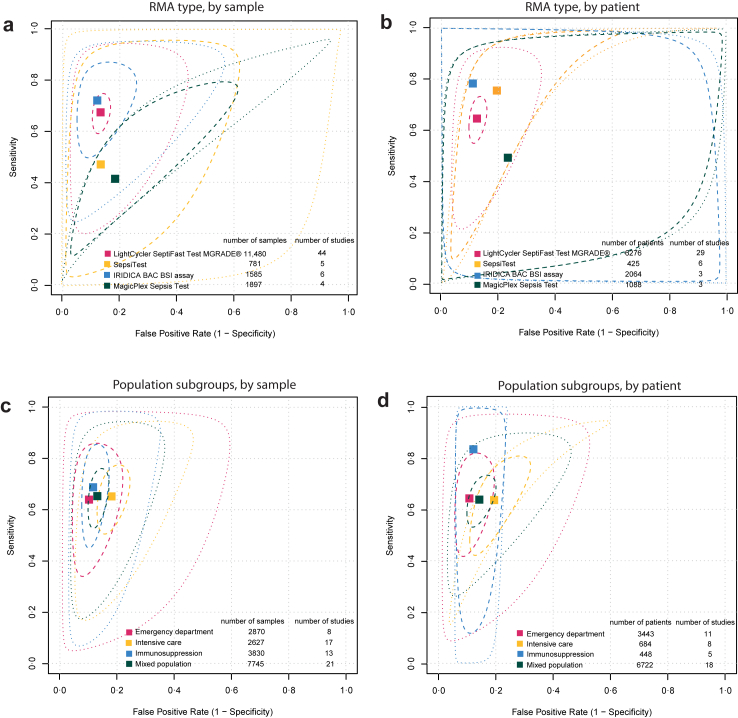
Subgroup analyses focusing on individual rapid molecular assays (RMA) and various patient populations. Bivariate summary estimates of sensitivity and specificity (filled squares) of RMAs compared to blood culture are shown with 95% confidence regions (dashed ellipses) and 95% prediction regions (dotted ellipses). A–B panels display diagnostic accuracies of different RMAs. C–D panels represent the pooled diagnostic accuracy of all RMAs in various patient populations. A) Diagnostic accuracy of individual RMAs (sample-level analysis). B) Diagnostic accuracy of individual RMAs (patient-level analysis). C) Diagnostic accuracy of all RMAs combined when restricting studies to specific patient populations (sample-level analysis). D) Diagnostic accuracy of all RMAs combined when restricting studies to specific patient populations. RMA = rapid molecular assay.

RMA-based subgroup analysis was performed for four assays (Septifast, IRIDICA, SepsiTest, and Magicplex) (Table 2). The remaining five RMAs included in the quantitative synthesis could not be analyzed in subgroups due to the limited number of studies (UMD-SelectNA, VYOO, MicrobScan assay, MicrobScan-Kairos24/7, and REBA Sepsis-ID test). Generally, RMAs had higher specificity than sensitivity, with a narrow range of specificities and a wider range of sensitivities (Fig. 3A and B, Table 2). IRIDICA had the highest sensitivity in both sample-level and patient-level analyses, while Magicplex had the lowest sensitivity at both levels (Table 2, Fig. 3A and B). IRIDICA’s sensitivity was significantly higher than Magicplex’s in both analyses (sample: p = 0.00029; patient: p = 0.0030) (Table 2, Fig. 3A and B, Supplementary Table S4). The sensitivity of Magicplex by sample was significantly lower than that of Septifast (p = 0.0026) after correction for multiple testing (Table 2, Supplementary Table S4). SepsiTest was the only RMA that showed a notable difference when analyzed separately by sample versus patient (Fig. 3A and B, Table 2). Heterogeneity was either high across subgroups, or CIs of *I*^*2*^ values were wide to properly assess heterogeneity (Supplementary Table S5).

**Table 2:** Subgroup analyses based on rapid molecular assay type, patient age category, and contaminant handling practices.

	Sensitivity (95% CI)	Specificity (95% CI)
Rapid molecular assay-based subgroup analysis (N)		
LightCycler SeptiFast Test MGRADE®		
Sample (44)	0.678 (0.615–0.734)	0.863 (0.837–0.886)
Patient (29)	0.645 (0.573–0.712)	0.875 (0.849–0.897)
IRIDICA BAC BSI assay		
Sample (6)	0.722 (0.611–0.811)	0.875 (0.813–0.919)
Patient (3)	0.783 (0.662–0.870)	0.890 (0.819–0.935)
SepsiTest		
Sample (5)	0.473 (0.175–0.791)	0.863 (0.689–0.947)
Patient (6)	0.755 (0.342–0.948)	0.805 (0.597–0.920)
MagicPlex Sepsis Test		
Sample (4)	0.417 (0.295–0.550)	0.813 (0.701–0.890)
Patient (3)	0.492 (0.390–0.594)	0.767 (0.658–0.849)
Patient age category-based subgroup analysis in the LightCycler SeptiFast Test MGRADE® subgroup (N)		
Adults		
Sample (21)	0.717 (0.637–0.786)	0.879 (0.844–0.906)
Patient (12)	0.639 (0.546–0.722)	0.884 (0.852–0.910)
Infants and neonates		
Sample (3)	0.806 (0.698–0.881)	0.621 (0.559–0.679)
Subgroup analysis based on contaminant reporting practices in the LightCycler SeptiFast Test MGRADE® subgroup (N)		
Contaminant handling was clearly reported		
Sample (33)	0.613 (0.551–0.672)	0.865 (0.833–0.892)
Patient (19)	0.559 (0.495–0.622)	0.888 (0.859–0.911)
Contaminant handling was unclear or not reported		
Sample (12)	0.812 (0.720–0.878)	0.871 (0.822–0.908)
Patient (10)	0.776 (0.644–0.869)	0.851 (0.803–0.889)

RMA = rapid molecular assay. CI = confidence interval. N = number of extracted reports.

Different units of analysis are displayed separately.

Age category-based (adult versus infant/newborn) subgroup analysis was performed for the Septifast device (Supplementary Figs. S3 and S4). Data were available for comparison only for the sample-level analysis (Supplementary Fig. S3). Sensitivities did not differ significantly between the subgroups (p = 0.16), but specificity was significantly higher in adults (0.879 versus 0.621, p < 0.0001) (Table 2).

Our analysis included studies that lacked detailed information or any reporting on contaminant handling. To address this, we performed a subgroup analysis based on how contaminants were reported. Pooled sensitivity was significantly higher in both sample-level (p = 0.00063) and patient-level analyses (p = 0.0048) for studies where contaminant handling was unclear or unreported (Table 2, Supplementary Figs. S5 and S6).

Pooled diagnostic accuracy metrics were analyzed across different well-defined patient populations (see Methods). Pooled sensitivity estimates were low across all subgroups in sample-based analyses, ranging from 0.653 (95% CI 0.561–0.735) in the mixed group to 0.688 (95% CI 0.525–0.814) in the immunosuppressed group, with no significant differences found between subgroups (Table 3, Supplementary Table S6). In patient-based analyses, sensitivity ranged from 0.591 (95% CI 0.459–0.710) in the ICU group to 0.834 (95% CI 0.497–0.963) in the immunosuppressed group (Table 3). Specificity was the highest in the ED population and lowest in the ICU population by both sample and patient. Specificity by sample was lower in the ICU population compared to other groups (ICU versus ED, p = 0.036; ICU versus immunosuppressed, p = 0.016; ICU versus mixed, p = 0.041); however, these differences were no longer significant after adjusting for multiple testing (Supplementary Table S6). Significant heterogeneity was observed in all subgroups, except for the immunosuppressed group when analyzed by patient (Supplementary Figs. S7 and S8).

**Table 3:** Patient population-based subgroup analysis.

	Sensitivity (95% CI)	Specificity (95% CI)
Population-based subgroup analysis (N)		
Intensive care		
Sample (17)	0.652 (0.547–0.744)	0.819 (0.774–0.857)
Patient (8)	0.591 (0.459–0.710)	0.811 (0.716–0.879)
Emergency department		
Sample (8)	0.640 (0.454–0.791)	0.898 (0.835–0.939)
Patient (11)	0.643 (0.492–0.770)	0.892 (0.838–0.930)
Immunosuppressed		
Sample (13)	0.688 (0.525–0.814)	0.884 (0.847–0.913)
Patient (5)	0.834 (0.497–0.963)	0.878 (0.835–0.911)
Mixed		
Sample (21)	0.653 (0.561–0.735)	0.869 (0.840–0.894)
Patient (18)	0.638 (0.561–0.709)	0.857 (0.817–0.890)

CI = confidence interval. N = number of extracted reports.

Different units of analysis are displayed separately.

Of the 75 articles included in the meta-analysis, 36 (48%) were funded by the manufacturer, 35 (46.66%) were not, and 4 (5.33%) did not report sponsorship (Supplementary Table S2). Only in studies of Septifast was there sufficient data to compare the pooled diagnostic metrics. There was no difference in sensitivity (sponsored: 0.629, 95% CI 0.547–0.704; not sponsored: 0.694, 95% CI 0.574–0.793, p = 0.36) or specificity (sponsored: 0.861, 95% CI 0.826–0.889; not sponsored: 0.856, 95% CI 0.793–0.902, p = 0.87) when analyzed by sample (Supplementary Table S7). The sensitivity of Septifast by patient was not associated with sponsorship status (sponsored: 0.585, 95% CI 0.469–0.692; not sponsored: 0.705, 95% CI 0.562–0.816, p = 0.18), yet specificity was higher in sponsored studies (sponsored: 0.888, 95% CI 0.854–0.915; not sponsored: 0.826, 95% CI 0.769–0.871, p = 0.031).

The question of whether the diagnostic accuracy of RMAs varies by the quantity of BC sets drawn was further investigated. Diagnostic metrics were similar in studies reporting the drawing of at least two BC sets compared with the pooled diagnostic estimates from studies that included all possible numbers of BC sets (Supplementary Table S8).

Risk of bias assessment results are shown in Supplementary Table S9. Applicability concern was unclear in 44 articles (58.67%) regarding the QUADAS-2 “reference standard” domain, due to poor description of the BC technique, unreported number of BC sets, or lack of information on contaminant handling. Blinding was often unreported for both index and reference tests but was deemed inconsequential regarding the outcomes since manipulation of the results is not feasible. In the QUADAS-2 flow and timing domain, three articles (4%) had a high risk due to inappropriate time intervals between the index and reference tests, while 21 studies (28%) had unclear risk due to a lack of data on these intervals. Our calculations were made assuming a perfect reference test, which is improbable, given the various factors affecting the diagnostic accuracy of BCs. Therefore, ROB in the reference standard domain was judged “high risk” in all articles. There was low risk in all QUADAS-C domains, except for one article with unclear risk. However, this extension was applied solely to four studies (5.33%) comparing more than one RMA to BC.^20^^,^^58^^,^^75^^,^^77^

The quality of evidence was assessed separately for articles with different units of analysis (Supplementary Fig. S9). The GRADE evidence tables showed very low certainty of evidence for the investigated outcomes. For ROB, the evidence was downgraded due to the high proportion of high and unclear risk in several domains in the QUADAS-2. Additionally, the evidence was further downgraded due to the indirectness and significantly high between-study heterogeneities.

The results of the publication bias assessment are displayed on funnel plots (Supplementary Fig. S10). Deek’s funnel-plot asymmetry test was not significant (p = 0.24) for patient-wise data, while it was significant (p = 0.0034) for sample-wise data.

Articles evaluating Septifast had sufficient data on the number of BC sets (at least two versus unclear and less than two) for comparison. A supplementary analysis of pooled diagnostic metrics for BC and Septifast showed no difference in the diagnostic accuracies of the BC and the Septifast for studies reporting at least two BC sets (Table 4, Supplementary Fig. S11).

**Table 4:** Pooled diagnostic metrics of the LightCycler SeptiFast Test MGRADE® and the blood culture with increasing the number of blood culture sets drawn.

Number of BC sets	Sensitivity of SF (95% CI)	Specificity of SF (95% CI)	Sensitivity of BC (95% CI)	Specificity of BC (95% CI)
≥2 BC sets	0.661 (0.417–0.929)	0.917 (0.839–0.988)	0.647 (0.417–0.960)	0.923 (0.865–0.990)
<2 or unclear number of BC sets	0.743 (0.541–0.974)	0.917 (0.851–0.983)	0.672 (0.513–0.897)	0.900 (0.842–0.985)

CI = confidence interval. BC = blood culture. SF = LightCycler SeptiFast Test MGRADE®.

## Discussion

In this systematic review and meta-analysis, we compared the diagnostic accuracy of commercial RMAs with BC for the first time. We reviewed 104 studies on RMAs and included 75 in the meta-analysis of sensitivity and specificity of nine RMAs compared with clinical BCs. We found that the RMAs studied had high specificities, but low and variable sensitivities when compared to BC. The pooled diagnostic metrics did not differ significantly between sample-based and patient-based studies. We then focused on the four RMAs for which sufficient data were available to conduct a subgroup analysis. There were notable differences between the populations studied, with RMAs having the lowest specificity in ICU patients.

The fact that the sensitivity range of the assessed RMAs is 0.42–0.78 suggests that they are not suitable to replace BC to diagnose BSI as a standalone diagnostic, considering the potentially fatal consequences of sepsis. The sensitivity of RMAs is dictated by the number of FN results (more FNs decrease sensitivity), which may be attributed to several factors: 1) limited number of pathogens on the RMA target panel, 2) low blood volume for RMA sample compared to BC, or 3) low bacterial load in blood samples. The first two factors are a function of the assay and sampling method, and they deserve attention. Most studies (including previous meta-analyses) include samples with pathogens found by BC but not covered by the RMA panel, which penalizes sensitivity. Only two of 75 studies included in our meta-analysis excluded off-panel samples,^98^^,^^110^ and this is a likely contributor to the low sensitivities observed. It is also important that RMAs utilize varying blood volumes, with Magicplex requiring 1 ml and IRIDICA requiring 5 ml of whole blood. The lower blood volume used by Magicplex, along with its smaller target panel, may explain its lower sensitivity.^26^ This aligns with IRIDICA and its previous version (PLEX-ID), where increasing the blood volume (from 1.25 ml to 5 ml) resulted in enhanced sensitivity.^42^ Although panel size may influence sensitivity, IRIDICA (panel >200 pathogens) and Septifast (panel 25 pathogens) have remarkably similar sensitivities, even though a large difference in their panel sizes. This might be attributable to the differences in assay techniques or the dominant effect of the panel – including the most frequent pathogens – on overall performance. Thus, there is much more to be understood about performance characteristics before RMAs can be solely relied upon as a primary diagnostic modality. Currently, there are several RMAs under development, highlighting the potential advancements in this field.^145^, ^146^, ^147^

We acknowledge the imperfect nature of the reference BC test. Since our analyses assumed BC to be 100% sensitive and specific, this assumption may have led to underestimated diagnostic accuracies of RMAs in this study, as seen in a previous meta-analysis where the same assumption was made.^26^ Consequently, the reported performances should be interpreted as relative to BC results rather than true bloodstream pathogen detection accuracies. This is supported by our subgroup analysis based on contaminant reporting, which showed higher sensitivities in studies with unclear or missing contaminant reporting. It can be partially explained by the possibility that in articles that did not report contaminants, BC+/RMA– results were judged as contaminants, decreasing FN RMA results, leading to higher RMA sensitivities. This emphasizes the importance of clear and accurate contaminant reporting in future research.

Although the poor rule-out performance (sensitivity) is counterweighted by a better rule-in performance (specificity), the 11–23% FP rate warrants further discussion. On one hand, it has been shown that prior AB therapy decreases culture yield and thus incorrectly labels TPs as FPs.^61^^,^^77^ Therefore, a FP result, especially in the setting of prior AB exposure, sometimes reflects true infection.^106^ Specificity was found to be the lowest in the ICU population where many patients are AB exposed, while it was highest among ED patients, most of whom are AB naive. Our findings are consistent with those of a previous meta-analysis and a prospective trial of Septifast, that observed lower BC yield rather than low RMA specificity in the ICU population.^23^^,^^89^ According to our subgroup analysis, specificity was higher in adults compared to the newborn-infant population, indicating a higher FP rate in infants and newborns. This may reflect higher contamination rates in this age group, as reported in the literature.^148^^,^^149^ Alternatively, pathogen detection by an RMA without culture positivity might not necessarily indicate bacteremia, but instead the presence of non-viable or nonrelevant contaminant pathogen cells^47^^,^^98^^,^^108^^,^^113^ from which deoxyribonucleic acid strands are amplified, leading to FP results. The converse argument can be made for RMA test performance in the ED setting, where an RMA-identified pathogen is more likely to represent true infection. Our findings suggest that in 11–23% of suspected sepsis cases, clinicians may start unnecessary AMT if relying solely on the RMA result.

As discussed, we acknowledge that the reference BC is itself an imperfect diagnostic test.^7^^,^^8^ Given that the diagnostic yield of BC increases with the number of BC sets drawn,^150^ the determination of RMA test characteristics may also be influenced by the number of BC sets obtained. Our results did not show an improvement in the diagnostic yield of BC with an increased number of sets. However, due to the wide confidence intervals, we cannot conclude that drawing more BC sets does not improve detection rates. Our results, nonetheless, highlight that BC is an imperfect reference standard. Specificity of Septifast remained the same, despite increasing the number of BC sets. Theoretically, this should improve BC pathogen detection rates (turning FPs into TPs if the BC detects the pathogen), leading to increased RMA specificity. Notably, we could not confirm this with our analysis. Our results suggest – albeit indirectly – that RMAs are likely to provide an added value to BC, by detecting a pathogen in some cases when it is not detected by BC, independent of the number of BC sets obtained. This partial overlap of detection capabilities of the two methods supports previous suggestions of combining methods to improve detection rates.^24^^,^^67^^,^^119^ Combination of the two methods could potentially provide benefits when fastidious and non-cultivable pathogens are suspected,^24^ or in the treatment of patients already receiving AMT. Despite these benefits, it is important to note that the use of RMAs is associated with increased costs and larger blood draw volumes, which can be challenging or unnecessary in certain patient groups.^26^^,^^139^^,^^151^

We included studies that utilized either sample- or patient-based units of analysis (or both) and performed independent grouped analyses based on the method employed. Although the different reporting approaches could potentially bias the diagnostic metrics obtained, we did not find a difference in overall sensitivity or specificity. To specifically quantify the influence of sample-versus patient-based analysis on resulting test characteristics, one would need to include articles that report both types of data with the exact number of samples and BC sets per patient, but this was not reliably reported.

The observed high between-study heterogeneities can be linked to several potential sources: 1) the inclusion of studies with a diverse study population; 2) the heterogeneous indication for sampling among articles (even though all articles included those with suspected sepsis or documented BSI); 3) the pooling of articles reporting different units of analysis, which has been suggested as a potential cause of heterogeneity,^22^^,^^26^ and 4) the pooling of different RMAs. Within the diverse study population, sex differences may have contributed to the observed heterogeneity. However, sex-based analysis could not be performed as studies did not report data separately by sex. Regarding variability due to combining different units of analysis, we found no such variation except for SepsiTest, which showed higher diagnostic metrics when analyzed by patient. The explanation for the significant difference between the sample-versus patient-based diagnostic metrics of SepsiTest potentially be due to the inclusion of two studies^60^^,^^95^ reporting 1.00 sensitivity in only the patient-based analysis.

The strengths and unique features of our analysis compared with prior meta-analyses include 1) targeting commercially available RMAs without pre-selecting manufacturers; 2) separately handling articles reporting different units of analysis; 3) including more than 100 articles in the review.

There are several limitations to this report. First, we included articles that lack information on contaminant handling that potentially biased FP, TP, FN, and TN values. Although these studies demonstrated higher sensitivities, they still remained suboptimal. Second, we included articles where samples with off-panel pathogens detected by BC were excluded and not reported, which may underrepresent clinically significant RMA “misses.” This was the case in only two studies,^98^^,^^110^ where the most commonly occurring pathogens were covered in the representative pathogen panels.^142^ Third, some parts of the population were underrepresented. Since anaerobic BCs are less commonly drawn from children, yet they are clinically important,^152^ our exclusion of studies performing only one BC bottle of the set may have resulted in the underrepresentation of the pediatric population. Limited evidence from low- and middle-income countries (LMICs), where antimicrobial resistance, sepsis incidence, and mortality are higher, limits the generalizability of our findings.^2^^,^^153^^,^^154^ Moreover, excluding non-English articles may have led to the underrepresentation of research from non-English-speaking regions, as seen in previous meta-analyses.^22^, ^23^, ^24^, ^25^, ^26^ Fourth, sex-based analysis could not be performed due to the lack of reported data in studies, which further limits the generalizability of this research. Fifth, only published, English-language, original research articles were included, which may have biased the study results and contributed to the observed publication bias. Additionally, we did not analyze pathogen-specific data, as this would have exceeded the scope of the current paper. Lastly, reporting details of paired BC/RMA samples were lacking in several articles, which limits the interpretability of the results.

To increase translatability into clinical practice,^155^^,^^156^ future studies should report precisely the patient-level counts, timing, and volume of blood obtained for BC and RMA sampling, as these were not consistently reported. Reporting blood volume is important because it influences the diagnostic performance of both BC and RMAs.^25^^,^^150^ In our opinion, sample-based analysis provides a more precise evaluation of diagnostic metrics for RMAs and should be the gold standard of analysis. We advocate for future studies to draw at least two BC sets to increase the pathogen detection yield of the reference standard. To ensure a more representative population, more studies from LMICs and non-English-speaking regions are needed. For existing and new device improvements, there is a need to further develop RMAs with higher sensitivity, for instance, by increasing the sample blood volume and widening the pathogen coverage.

In summary, rapid molecular assays still lack the sensitivity to replace blood culture as a standalone diagnostic. Our findings do not provide evidence to change current guidelines regarding the use of rapid molecular assays in the diagnosis of bloodstream infections. Our data suggest that rapid molecular assays may have value as an add-on test to increase overall pathogen detection rates. Further research, including direct comparative and high-quality implementation studies, is needed to better define the role of RMAs in clinical care.

## Contributors

GAR, FAM, DG, and BGF designed the study. BGF provided study supervision. GAR, MR, and UNDT screened and selected studies. GAR and UNDT extracted and verified the data. TK and AW performed the statistical analysis. GAR, DC, LVK, VEK, PH, SV, CV, MRF, and BGF interpreted the results. GAR, VEK, and BGF wrote the first draft of the manuscript, and all other co-authors critically revised subsequent drafts. All authors read and approved the final version of the manuscript. All authors had full access to all the data in the study and accept responsibility for the decision to submit for publication.

## Data sharing statement

The study protocol is available at PROSPERO (CRD42022377280). The protocol was specified during the duplicate removal, expanding the exclusion criteria with “not commercially available/commercially developed rapid molecular assay” and “sample size less than 11”. The datasets generated and analyzed are published in the appendix. Additional information collected from studies and the analytic codes applied are available from the corresponding author upon reasonable request. All data used in this study can be found in the full-text articles included in this systematic review and meta-analysis.

## Declaration of interests

MRF received grant funding from Day Zero Diagnostics to conduct a clinical study of a genomics-based rapid pathogen detection technology. All other authors declare no competing interests.
