# Supplementary material for: Rapid molecular assays versus blood culture for bloodstream infections: a systematic review and meta-analysis
Source: eClinicalMedicine. 2025 Jan 10;79:103028. doi: 10.1016/j.eclinm.2024.103028 (PMC11833021; doi:10.1016/j.eclinm.2024.103028)
Supplement: Supplementary Material [file mmc1.pdf]

Supplementary appendix

Title

Rapid molecular assays versus blood culture for bloodstream infections: a systematic review and meta-analysis

Authors

Gabriella Anna Rapszky[1,2], Uyen Nguyen Do To[2,3], Veronika Eszter Kiss[1], Tamás Kói[2,4], Anna Walter[5], Dorottya Gergő[2,6], Fanni Adél Meznerics[2,7], Márton Rakovics[2,8], Szilárd Váncsa[2,5,9], Lajos Vince Kemény[2,7,10,11], Dezső Csupor[5,12], Péter Hegyi[2,5,9], Michael R. Filbin[2,13], Csaba Varga[1,2], Bank G. Fenyves[1,2,14]

Affiliations

1. Department of Emergency Medicine, Semmelweis University, Budapest, Hungary
2. Centre for Translational Medicine, Semmelweis University, Budapest, Hungary
3. András Pető Faculty, Semmelweis University, Budapest, Hungary
4. Budapest University of Technology and Economics, Department of Stochastics, Budapest, Hungary
5. Institute for Translational Medicine, Medical School, University of Pécs, Pécs, Hungary
6. Department of Pharmacognosy, Semmelweis University, Budapest, Hungary
7. Department of Dermatology, Venereology and Dermatooncology, Semmelweis University, Budapest, Hungary
8. Eötvös Loránd University, Faculty of Social Sciences, Department of Statistics, Budapest, Hungary
9. Institute of Pancreatic Diseases, Semmelweis University, Budapest, Hungary
10. Department of Physiology, Semmelweis University, Budapest, Hungary
11. HCEMM-SU, Translational Dermatology Research Group, Semmelweis University, Budapest, Hungary
12. Institute of Clinical Pharmacy, University of Szeged, Szeged, Hungary
13. Department of Emergency Medicine, Massachusetts General Hospital and Harvard Medical School, Boston, MA, USA
14. Department of Molecular Biology, Semmelweis University, Budapest, Hungary

Table of content

Supplementary Methods....4

Details of the systematic search 4

Details of the updated systematic search....4

Supplementary Tables....5

Table S1. The QUADAS-2 tool for the methodological assessment of diagnostic studies adapted from Stevenson et al....5

Table S2. Studies included in the meta-analysis 7

Table S3. Studies included in the systematic review only....14

Table S4. Examining the difference between diagnostic measures of different devices. Different units of analysis are displayed separately.....17

Table S5. Heterogeneities of diagnostic metrics across RMA type-based subgroups. Different units of analysis are displayed separately....18

Table S6. Examining the difference between diagnostic measures of rapid molecular assays subgrouped by patient population. Different units of analysis are displayed separately. 19

Table S7. Examining the difference between diagnostic measures of sponsored vs not sponsored articles regarding the LightCycler SeptiFast Test MGRADE®. Articles reporting different units of analysis were analyzed separately. 20

Table S8. Subgroup analysis based on the number of blood culture sets drawn 21

Table S9. Risk of bias assessment of included studies....22

Supplementary Figures....26

Figure S1. Pooled sensitivity and specificity of rapid molecular assays for the diagnosis of bloodstream infections when compared to blood culture. Unit of analysis: patient....26

Figure S2. Bivariate summary estimates of sensitivity and specificity for rapid molecular assays (RMA) when compared to blood culture with 95% confidence (dashed) and 95% prediction ellipses (dotted). 27

Figure S3. Age category-based subgroup analysis in the LightCycler SeptiFast Test MGRADE® subgroup. 28

Figure S4. Age category-based subgroup analysis in the LightCycler SeptiFast Test MGRADE® subgroup. Age category: adult population. Unit of analysis: patient. 29

Figure S5. Subgroup analysis of articles that properly reported contaminants in the LightCycler SeptiFast Test MGRADE® subgroup....30

Figure S6. Subgroup analysis of articles that either did not report contaminants properly or did not report them all in the LightCycler SeptiFast Test MGRADE® subgroup. 31

Figure S7. Pooled specificity and sensitivity of rapid molecular assays when compared to blood culture. Population-based subgroup analyses are displayed in A-D plots. Unit of analysis: sample....32

Figure S8. Pooled specificity and sensitivity of rapid molecular assays when compared to blood culture. Population-based subgroup analyses are displayed in A-D plots. Unit of analysis: patient. 33

Figure S9. Summary of evidence tables (GRADE approach) 34

Figure S10. The Deek's funnel-plot asymmetry test. Filled dots represent individual studies, dashed line represents the regression line....35

Figure S11. Supplementary analysis. Receiver operating curve (ROC) plots of the diagnostic accuracy of the LightCycler SeptiFast Test MGRADE®and blood culture with 95% confidence (ellipses with dashed lines) and 95% prediction regions (ellipses with continuous lines). 36

References 37
Supplementary Data 42
Data S1. Data extracted and analyzed from studies included in the quantitative synthesis 42
Studies excluded at full-text screening stage with brief reasons 50

Supplementary Methods

Details of the systematic search

Databases screened and number of articles found with the query:
Medline (18,477), Embase (24,376), CENTRAL (1,400), Web of Science (10,906)

Date of search:
January 10, 2023

Search query:
(blood culture OR culture OR hemoculture OR bloodculture) AND (blood poison OR blood poisoning OR blood stream OR bloodstream OR sepsis OR septic OR bacteremia OR septicemia OR fungemia OR candidemia OR candidaemia OR viremia OR invasive candidiasis OR invasive aspergillosis) AND (molecular OR technique OR method* OR assay OR device OR array OR tool* OR panel OR test OR tests OR Septifast OR Magicplex OR VYOO OR Sepsitest OR PCR OR qPCR OR polymerase chain reaction OR real-time) AND (rapid* OR earl* OR fast* OR quick* OR turnaround time* OR time OR time* OR timely OR duration OR period* OR period) AND (diagnosis OR diagnostic OR diagnose OR identif* OR detect* OR recog* OR sensitivity OR sensitive OR specificity OR specific OR „true positive” OR „true positives” OR „true negative” OR „true negatives” OR „false positive” OR „false positives” OR „false negative” OR „false negatives”)

Details of the updated systematic search

Databases screened and number of articles found with the query:
Medline (20,124), Embase (28,776), CENTRAL (1,630), Web of Science (13,380)

Date of search:
September 23, 2024

Search query:
No modifications were made to the search query.

Supplementary Tables

Table S1. The QUADAS-2 tool for the methodological assessment of diagnostic studies adapted from Stevenson et al[1]

	Scoring	Summary judgment
**Risk of bias**		
**Patient selection**		
Was a consecutive or random sample of patients enrolled?	'Yes' if authors mention consecutive/random or referring to these methods.	Could the selection of patients have introduced bias?<br><br>'Low risk'<br>According to Stevenson et al.<br><br>'High risk'<br>According to Stevenson et al.<br><br>'Unclear risk'<br>According to Stevenson et al.
	'No'<br>According to Stevenson et al.	
	'Unclear'<br>According to Stevenson et al.	
Was a case-control design avoided?	'Yes'<br>According to Stevenson et al.	
	'No'<br>According to Stevenson et al.	
	' Unclear'<br>According to Stevenson et al.	
Did the study avoid inappropriate exclusions?	'Yes'<br>According to Stevenson et al.	
	'No'<br>In case of excluding samples or patients with microorganisms not on the target list	
	' Unclear'<br>According to Stevenson et al.	
**Index test**		
Were the index test results interpreted without knowledge of the results of the reference standard?	'Yes'<br>According to Stevenson et al.	Could the conduct or interpretation of the index test have introduced bias?<br><br>Since manipulation of the results is impossible in this case, the answer to this question was 'Low risk' in each case.
	'No'<br>According to Stevenson et al.	
	' Unclear'<br>According to Stevenson et al.	
If a threshold was used, was it pre-specified?	In this review we considered the current question irrelevant.	
**Reference standard**		
Is the reference standard likely to correctly classify the target condition?	Since the „gold standard” in our review is not 100% sensitive and specific, the answer to this question was 'No' in each case.	Could the reference standard, its conduct, or its interpretation have introduced bias?<br><br>Due to the answer to the first signaling question, the judgment was 'High risk' in each case.
Were the reference standard results interpreted without knowledge of the results of the index test?	'Yes'<br>According to Stevenson et al.	
	'No'<br>According to Stevenson et al.	
	' Unclear'<br>According to Stevenson et al.	
**Flow and timing**		
Was there an appropriate interval between index test(s) and reference standard?	'Yes' in case authors mentioned sampling from the same venipuncture/sampling dor the index test and blood culture right after each other/sample for blood culture was drawn immediately after the sampling for the index test/samples were taken at the same time", or synonyms.	Could the patient flow have introduced bias?<br><br>'No' answer to the first question potentially introduces a high risk of bias.<br>Answers to all other signaling questions to this domain were insufficient to increase the risk of bias to „high risk", therefore the answer to this question was tailored to the first signaling question.<br><br>If the answer was 'Yes'<br>'Low risk'.<br><br>If the answer was 'No'<br>'High risk'.
	'No' in case of sampling for the index and reference test did not happen at the same time (e.g. 1, 2, 4, 12, 24 hours apart).	
	' Unclear'<br>According to Stevenson et al.	
Did all patients receive a reference standard?	'Yes'<br>According to Stevenson et al.	
	'No'	

	According to Stevenson et al.	If the answer was ’Unclear’ ’Unclear risk’.
	’ Unclear’ According to Stevenson et al.	
Did all patients receive the same reference standard?	Studies were excluded in which the reference standard was other than blood culture, in all extracted studies patients’ index results were compared to the blood culture, therefore this question was marked ’Yes’ in each case.	
Were all patients included in the analysis?	’Yes’ According to Stevenson et al.	
	’No’ According to Stevenson et al.	
	’ Unclear’ According to Stevenson et al.	
**Applicability domains**		
**Patient selection**		
Are there concerns that the included patients and setting do not match the review question?	Since all articles with suspected septic patients or patients with documented bloodstream infection were excluded from the analysis, the answer to this question was ’No’.	
**Index test**		
Could the conduct or interpretation of the index test have introduced bias?	If the authors mentioned ’according to the manufacturers”, or the standard protocol was followed, we answered ’No’.<br>In case if only 1-2 articles were available with the rapid molecular assay, we considered the first article’s protocol as the „standard protocol”.	
	If insufficient data was available we answered ’Unclear’.<br>If authors did not report on the handling of contaminants, we answered ’Unclear’.	
	If there were modifications to the standard procedure, we answered ’Yes’.	
**Reference standard**		
Is there concern that the target condition, as defined by the reference standard, does not match the review question?	In case if „standard laboratory procedure” (including drawing at least 2 blood culture sets) was mentioned, the answer was ’No’.	
	If insufficient data was available we answered ’Unclear’.<br>If authors did not report on the handling of contaminants or the number of blood culture sets drawn, we answered ’Unclear’.	
	If less than 2 blood culture sets were drawn, we answered ’Yes’.	

Table S2. Studies included in the meta-analysis

Author (year)	Study type	Country	Number of patients	Number of samples	Age/age category	Female sex (%)	Male sex (%)	Index test	Unit of extracted data („Unit of analysis”)	reason for sampling	Patient population	Spon-sor-ship
Avolio et al (2010)[2]	prospective cohort	Italy	144	144	Mean 51 (range 20-89)	41·6	58·4	LightCycler SeptiFast Test MGRADE®	sample and patient	suspected bloodstream infection	ED	?
Avolio et al (2014)[3]	prospective cohort	Italy	830	NA	adults	NA	NA	LightCycler SeptiFast Test MGRADE®	patient	suspected sepsis	mixed (ED and ICU)	?
Bacconi et al (2014)[4]	prospective cohort	USA	331	331	adults	NA	NA	IRIDICA BAC BSI assay	sample and patient	Suspected sepsis	ED	✓
Bloos et al (2012)[5]	observational trial	Germany	245	336	Median 67 (IQR 55-74)	39·4	60·6	VYOO®	sample	suspected sepsis	ICU	✓
Bloos et al (2010)[6]	prospective non-randomized controlled trial	France, Germany	205	347	adults	30·7	69·3	LightCycler SeptiFast Test MGRADE®	sample	severe sepsis/septic shock	ICU	✓
Bravo et al (2011)[7]	NR	Spain	33	33	Median 58 (range 24-80)	45·0	55·0	LightCycler SeptiFast Test MGRADE®	sample and patient	fever	immunosuppression (hematology unit)	X
			53	53	Median 65·5 (range 23-86)	37·7	62·3				ICU	
Cambau et al (2017)[8]	cluster-randomized trial	France	1416	NA	Median 59 (IQR 48-70)*	34·6*	65·4*	LightCycler SeptiFast Test MGRADE®	patient	febrile neutropenia/severe sepsis/suspicion of infective endocarditis	mixed (ICU and other)	X
Carrara et al (2013)[9]	prospective cohort	Barcelona	267	267	adults	NA	NA	MagicPlex Sepsis Test	sample and patient	suspected sepsis	mixed (ICU, hematology and ED)	X
Casalta et al (2009)[10]	NR	France	63	63	NA	NA	NA	LightCycler SeptiFast Test MGRADE®	patient	patients with infective endocarditis	NA	✓
Chaidaroglou et al (2013)[11]	NR	Greece	30	130	Median 42 (range 20-65)	20·0	80·0	LightCycler SeptiFast Test MGRADE®	sample	Suspected bloodstream infection in thoracic allograft recipients	immunosuppression (cardiac surgery center)	X

Desmet et al (2016)[12]	prospective cohort	Belgium	74	106	Mean 52 (range 19-73)	39·0	61·0	IRIDICA BAC BSI assay	sample	febrile neutropenia	immunosuppression (acut leukemia and allogeneic stem cell transplantation unit)	✓
Dierkes et al (2009)[13]	retrospective cohort	Germany	77	101	Median 55	36·4	63·6	LightCycler SeptiFast Test MGRADE®	sample and patient	suspected sepsis	mixed (surgical and other medical wards)	✓
Fernández-Romero et al (2014)[14]	prospective cohort	Spain	81	96	Mean 58, median 63 (range 21-96)	45·0	55·0	LightCycler SeptiFast Test MGRADE®	sample	suspected sepsis	mixed (nephrology department)	✓
Fitting et al (2012)[15]	prospective cohort	Brazil	86	NA	Median 73 (range 55-83)	37·0	23·0	VYOO®	patient	suspected sepsis/severe sepsis/septic shock	ICU	✓
Gosiewski et al (2014)[16]	prospective cohort	Poland	NA	71	adults	NA	NA	LightCycler SeptiFast Test MGRADE®	sample	suspected sepsis	ICU	X
Greco et al (2018)[17]	retrospective cohort	Italy	514	1837	Mean 50±15	39·3	60·7	LightCycler SeptiFast Test MGRADE®	samples	febrile neutropenia	immunosuppression (hematology and BMT unit)	X
Grijalva et al (2020)[18]	clinical trial	Ecuador	255	255	Mean 54·2±17·5	34·5	65·5	LightCycler SeptiFast Test MGRADE®	sample and patient	suspected sepsis	ICU	X
Grosse-Onnebrink et al (2017)[19]	prospective cohort	Germany	28	72	Median 21 (IQR 16-23)	39·3	60·7	LightCycler SeptiFast Test MGRADE®	sample	CF patients with fever/ CF patients hospitalized for infective pulmonary exacerbation	mixed (hospitalized patients)	X
Grosso et al (2021)[20]	prospective cohort	Italy	307	307	NA	NA	NA	MicrobScan assay	sample and patient	suspected sepsis	mixed (hospitalized patients)	✓
			441	441	NA	NA	NA	MicrobScan-Kairos24/7 assay				
Guido et al (2012)[21]	NR	Italy	166	166	Median 66·1 (range 23-82)	38·0	62·0	LightCycler SeptiFast Test MGRADE®	sample and patient	neutropenic patients with suspected sepsis (with hematological malignancies)	immunosuppression (hematology department)	?
Haag et al (2013)[22]	retrospective cohort	Germany	66†	96‡	Mean 58§ (all study population) Mean 61±24 (range 48-87) (female) Mean 55±22 (range 18-87) (male) (range 18-87)¶	24·0¶	76·0¶	SepsiTest	patient	septicemia	mixed (orthopedics hospital, cardiac clinic, general hospital wards)	X

Herne et al (2013)[23]	retrospective cohort	Estonia	144	160	Mean 58 (range 20-81)	42·4	57·6	LightCycler SeptiFast Test MGRADE®	sample and patient	suspected sepsis/septic shock	mixed (ICU, abdominal surgery, orthopedics, cardiology units)	X
Hettwer et al (2012)[24]	prospective cohort	Germany	211‖	211‖	Mean 62·8±17·7**	39·2**	60·8**	LightCycler SeptiFast Test MGRADE®	sample and patient	suspected sepsis	ED	✓
Idelevich et al (2015)[25]	RCT	Germany	150	253	Mean 52·4	40·7	59·3	LightCycler SeptiFast Test MGRADE®	sample	febrile neutropenia/suspected sepsis in neutropenic patients	immunosuppression (haematologic malignancy, department of medicine)	✓
Jordana-Lluch et al (2015)[26]	prospective cohort	Spain	405††	410††	Median 55·7 (range 16-83) ‡‡	36·9‡‡	63·1‡‡	IRIDICA BAC BSI assay	sample	suspected sepsis	ICU and ED	✓
Jordana-Lluch et al (2017)[27]	prospective cohort	Spain	429	463	Mean 59·9 (range 17-91)	35·4	64·6	IRIDICA BAC BSI assay	sample	suspected sepsis	immunosuppression (hematology and oncology ward, ED)	✓
Josefson et al (2011)[28]	prospective cohort	Sweden	1093	1093	Median 67 (range 14-98)	44·0	56·0	LightCycler SeptiFast Test MGRADE®	sample and patient	suspected sepsis	mixed (infectious diseases department)	✓
Kim et al (2020)[29]	prospective cohort	Korea	440	NA	≥16 years	NA	NA	REBA Sepsis-ID test	patient	suspected sepsis	ED	X
Korber et al (2017)[30]	retrospective cohort	Austria	398	470	NA	NA	NA	LightCycler SeptiFast Test MGRADE®	sample	sepsis, endocarditis, FUO, pneumonia, immunosuppression	mixed (internal, surgical wards, ICU, ED and outpatient units)	✓
Kühn et al (2011)[31]	prospective cohort	Germany	30	28	Mean 68 (range 51-84) (female) Mean 60 (range 37-78) (male)	20·0	80·0	SepsiTest	sample and patient	patients operated for infective endocarditis	mixed (cardiothoracic, transplant and cardiovascular surgery)	?
Lehmann et al (2010)[32]	prospective cohort	Germany	108	453	Mean 60·1 (range 19-84) (female) Mean 57·42 (range 18-84) (male)	33·3	66·7	LightCycler SeptiFast Test MGRADE®	sample	suspected sepsis	intensive care unit	✓
Leli et al (2014)/a[33]	retrospective cohort	Italy	571	NA	Median 71 (IQR 54-82)	41·3	58·7	LightCycler SeptiFast Test MGRADE®	patient	suspected sepsis	mixed (medical, surgical and intensive care units)	?
Leli et al (2014)/b[34]	retrospective cohort	Italy	285	285	Median 62·9 (IQR 51-81)	40·0	60·0	LightCycler SeptiFast Test MGRADE®	sample and patient	suspected sepsis	mixed (internal medicine, gastroenterology, surgical ward, cardiology, infectious diseases, pediatrics,	?

											nephrology, oncohematologic, ICU)	
Liotti et al (2019)[35]	prospective cohort	Italy	229	229	adults and children	NA	NA	MicrobScan assay	sample	suspected bloodstream infection	mixed (hospitalized patients)	✓
Lodes et al (2012)[36]	prospective cohort	Germany	104	148	Mean 63·1 (range 20-88)	28·8	71·2	LightCycler SeptiFast Test MGRADE®	sample	suspected sepsis (abdominal origin)	ICU	X
Loonen et al (2014)[37]	retrospective cohort	The Netherlands	125	125	Mean 68·9±17·3 (blood culture positive patients) Mean 60·4±18 (blood culture negative patients)	40·8	59·2	SepsiTest; MagicPlex Sepsis Test	sample and patient	suspected sepsis	ED	✓
Lucignano et al (2011)[38]	retrospective cohort	Italy	811	1673	Range 0-18	NA	NA	LightCycler SeptiFast Test MGRADE®	sample	suspected sepsis	mixed (ICU, surgery department, oncology-hematology, neonatology, ED)	X
Mahmoud et al (2023)[39]	prospective cohort	Egypt	120§§	120§§	Median 7 (IQR 4-13)	42·5	57·5	LightCycler SeptiFast Test MGRADE®	sample and patient	suspected sepsis	immunosuppression (cancer patients)	X
Makristathis et al (2018)[40]	prospective cohort	Austria	193	193	Median 56 (IQR 48-68) (patients with bloodstream infection) Median 57 (IQR 37-69) (patients without bloodstream infection)	33·7	66·3	LightCycler SeptiFast Test MGRADE®; IRIDICA BAC BSI assay	sample	suspected sepsis	mixed (ED, ICU, internal medicine ward)	✓
Mancini et al (2008)[41]	NR	Italy	34	103	Mean 47 (range 21-69)	32·4	67·6	LightCycler SeptiFast Test MGRADE®	sample	Neutropenic patients with suspected sepsis (with hematological malignancies)	immunosuppression (hematology unit)	✓
Markota et al (2014)[42]	prospective cohort	Slovenia	57	63	Mean 59·5±14·8	33·3	66·7	LightCycler SeptiFast Test MGRADE®	sample	severe sepsis/septic shock	ICU	?
Maubon et al (2010)[43]	prospective cohort	France	110	110	Mean 56·3±13·7	39·1	60·9	LightCycler SeptiFast Test MGRADE®	sample and patient	suspected sepsis in patients with malignancies	immunosuppression (hospitalized patients)	✓
Mauro et al (2012)[44]	NR	Italy	79	79	Range 5-68	51·9	48·1	LightCycler SeptiFast Test MGRADE®	sample and patient	suspected sepsis	immunosuppression (hospitalized patients)	?
Metzgar et al (2016)[45]	prospective cohort	USA	285	285	NA	NA	NA	IRIDICA BAC BSI assay	sample and patient	suspected sepsis	ED	?

Mongelli et al (2015)[46]	prospective cohort	Italy	65	138	Mean 54 (range 18-85)	40·0	60·0	LightCycler SeptiFast Test MGRADE®	sample	febrile patients with suspected bacteremia	ICU	?
Nieman et al (2016)[47]	prospective cohort	Germany, The Netherlands	166	236	Mean 65 (range 26-92)	38·0	62·0	SepsiTest	sample	suspected sepsis	ICU	✓
Obara et al (2011)[48]	prospective cohort	Japan	54	78	Mean 61·6 (range 30-86)	35·2	64·8	LightCycler SeptiFast Test MGRADE®	sample	suspected sepsis	mixed (surgery, ED, ICU, hematology and pulmonology unit)	✓
Ortiz Ibarra et al (2015)[49]	retrospective cohort	Mexico	86	86	newborns	31·3	68·7	LightCycler SeptiFast Test MGRADE®	sample and patient	suspected sepsis	NA	X
Ozkaya-Parlakay et al (2014)[50]	prospective cohort	Turkey	69	79	Mean 2·71±4·11 (range 0-17)	37·7	62·3	LightCycler SeptiFast Test MGRADE®	sample	severe sepsis/septic shock	mixed (ICU and other)	X
Paolucci et al (2013)[51]	prospective cohort	Italy	201	437	adults and pediatric	NA	NA	LightCycler SeptiFast Test MGRADE®	sample	febrile neutropenia in patients with hematological malignancies	immunosuppression (institute of hematology and oncology)	✓
Pasqualini et al (2012)[52]	prospective cohort	Italy	391	391	Median 73 (IQR 20-99)	45·0	55·0	LightCycler SeptiFast Test MGRADE®	sample and patient	suspected sepsis	mixed (internal medicine ward)	?
Rath et al (2012)[53]	prospective cohort	Germany	170	225	Mean 52·6±10·9 (range 27-70) (patients in the liver transplantation group) Mean 60·2±13·2 (range 28-88) (patients outside the liver translpantation group)	44·0	56·0	LightCycler SeptiFast Test MGRADE®	sample	suspected sepsis (after LTX or abdominal surgery)	ICU	?
Ratzinger et al (2016)[54]	prospective cohort	Austria	220	NA	Median 56.5 (IQR 41.5-68.0)	42·3	57·7	LightCycler SeptiFast Test MGRADE®	patient	suspected sepsis	mixed (surgical and medical wards)	X
Regueiro et al (2010)[55]	prospective cohort	Spain	72	106	Mean 64 (range 21-92)	26·0	74·0	LightCycler SeptiFast Test MGRADE®	sample	suspected sepsis	ICU	X
Reyna Figueroa et al (2019)[56]	case-control	Mexico	60¶¶	60¶¶	Mean 12±7 (cases) Mean 13±10 (controls)	38·3***	61·7***	LightCycler SeptiFast Test MGRADE®	sample and patient	suspected sepsis	ICU	X
Rodrigues et al (2019)[57]	RCT	Brazil	200	200	Median 65 (IQR 57-75) (intervention group)	29·0 (control) 34·0	71·0 (control) 66·0	LightCycler SeptiFast Test MGRADE®	patient	septic patients	mixed (Heart Institute)	X

					Median 64 (IQR 56-72) (control group)	(interve ntion)	(interve ntion)					
Rogina et al (2014)[58]	prospective cohort	Slovenia	23	NA	Median 59 (range 24-88)	NA	NA	SepsiTest	patient	suspected sepsis	ED	X
Schaub et al (2014)[59]	prospective cohort	Switzerland	110	205	Median 64	40·0	60·0	LightCycler SeptiFast Test MGRADE®	patient	suspected sepsis	ED	✓
Schreiber et al (2013)[60]	prospective cohort	Germany	50	50	Median 64 (IQR 51-70)	20·0	80·0	LightCycler SeptiFast Test MGRADE®<br>SepsiTest<br>VYOO®	sample and patient	suspected sepsis	ICU	✓
Sitnik et al (2014)[61]	prospective cohort	Brazil	114	114	Mean 49·7±24·8	35·1	64·9	LightCycler SeptiFast Test MGRADE®	sample and patient	suspected sepsis	mixed (ICU, ED, and oncology ward)	✓
Strålin et al (2020)[62]	prospective cohort	USA, United Kingdom, Sweden	1501	NA	Median 54 (range 6-96)	41·6	58·4	IRIDICA BAC BSI assay	patient	suspected sepsis	mixed (ED, ICU and other)	✓
Suberviola et al (2016)[63]	prospective cohort	Spain	119	119	Mean 63·5±12·8	37·0	63·0	LightCycler SeptiFast Test MGRADE®	sample	severe sepsis/septic shock	ICU	✓
Tafelski et al (2015)[64]	RCT	Germany	78	78	Median 59 (IQR 47-67) (control group) Median 67 (IQR 53-74) (intervention group)	36·0	64·0	LightCycler SeptiFast Test MGRADE®	sample and patient	suspected sepsis (pulmonary or abdominal origin)	ICU	✓
Tat Trung et al (2018)[65]	NR	Vietnam	110	110	NA	NA	NA	LightCycler SeptiFast Test MGRADE®	sample and patient	septic patients	mixed (hospitalized patients)	X
Tkadlec et al (2019)[66]	prospective cohort	Czech Republic	973	1370†††	NA	NA	NA	UMD-SelectNA	sample	suspected bloodstream infection	mixed (hospitalized or outpatient units)	X
Tran et al (2012)[67]	prospective cohort	USA	50	NA	Mean 41·55	18·0	82·0	LightCycler SeptiFast Test MGRADE®	patient	suspected sepsis	ICU	✓
Tröger et al (2016)[68]	prospective cohort	Germany	133	214	Median in weeks: 25·6 (IQR 24·5-28·4)	53·5	46·5	LightCycler SeptiFast Test MGRADE®	sample	suspected sepsis	NA	X

Tsalik et al (2010)[69]	prospective cohort	England	306	306	Mean 54·1 (range 18-97)	45·1	54·9	LightCycler SeptiFast Test MGRADE®	sample and patient	suspected sepsis	ED	X
Varani et al (2009)[70]	NR	Italy	100	154	adults and children	NA	NA	LightCycler SeptiFast Test MGRADE®	sample	Immunosupressed patients with suspected sepsis	immunosuppression (institute of hematology and oncology)	✓
Wallet et al (2009)[71]	prospective cohort	France	72	100	adults	NA	NA	LightCycler SeptiFast Test MGRADE®	sample and patient	suspected sepsis	ICU	✓
Wang et al (2015)[72]	prospective cohort	Korea	882	882	Mean 59·7±19·7 (range 19-96)	43·5	56·5	REBA Sepsis-ID test	sample and patient	suspected sepsis	ED	X
Wellinghausen et al (2009)[73]	prospective cohort	Germany	187	342	adults and children	NA	NA	SepsiTest	sample and patient	SIRS, sepsis, neutropenic fever or fever in haematooncologic patients	mixed (departments of medicine, pediatrics and surgical ICU)	X
Westh et al (2009)[74]	NR	Germany	359	613	NA	NA	NA	LightCycler SeptiFast Test MGRADE®	sample	suspected sepsis	NA	✓
Zboromyrska et al (2019)[75]	prospective cohort	Spain	636	809	Mean 56·7±5·6	43·0	57·0	MagicPlex Sepsis Test	sample	suspected bloodstream infection	mixed (ED, ICU, surgery unit, oncology and hematology and other units)	X
Ziegler et al (2016)[76]	NR	Sweden	696	696	Median 67 (range 18-97)	42·0	58·0	MagicPlex Sepsis Test	sample and patient	suspected sepsis	ED	✓

✓ indicates sponsorship by the manufacturer. X indicates no sponsorship by the manufacturer. ? indicates no information regarding the sponsorship by the manufacturer.
NA=not available. NR=not reported. IQR=interquartile range. CF=cystic fibrosis. FUO= fever of unknown origin. LTX=liver transplantation. ED=emergency department. ICU=intensive care unit. RCT=randomized controlled trial.
* data regarding 731 patients solely from the intervention period
† whole blood was drawn from 12 patients, thus 12 patients were included in the analysis
‡ other sample types were included besides whole blood. Data regarding whole blood was not extractable.
§ data regarding 66 patients
¶ data regarding 12 patients
|| blood culture was available for 113 samples and patients, thus only those were included in the analysis
** data regarding the whole study population
†† data were available from 222 ICU patients, thus only those were included in the analysis
‡‡ data regarding the whole study population
§§ rapid molecular assay was performed for 60 patients, thus only 60 patients and 60 samples were included in the analysis
¶¶ 40/60 patients were controls in the case-control study, thus only 20 patients and 20 samples were included in the analysis
*** data regarding the whole study population
††† from 1370 different sample types, 848 whole blood samples were included in the analysis

Table S3. Studies included in the systematic review only

Author (year)	Reason for exclusion from the data synthesis	Study type	Country	Number of patients	Number of samples	Age/age category	Female sex (%)	Male sex (%)	Index test	Unit of data extraction	reason for sampling	Patient population	Sponsorship
Burdino et al (2014)[77]	excluded contaminants	prospective cohort	Italy	1024	1186	NA	NA	NA	LightCycler SeptiFast Test MGRADE®	sample	suspected sepsis	mixed (infectious diseases department, ICU, ED, cardiology, internal medicine, surgery)	X
Camp et al (2020)[78]	insufficient data	NR	Austria	232	365	NA	NA	NA	LightCycler SeptiFast Test MGRADE®	NA	suspected sepsis	mixed (hospitalized patients)	X
Camp et al (2024)[79]	inappropriate time interval between the index and refernce tests	prospective cohort	Austria	138	177	Median 61 (range 20-84)	31·9	68·1	LightCycler SeptiFast Test MGRADE®	sample	suspected candidemia	ICU	✓
Cortegiani et al (2014)[80]	insufficient data	retrospective cohort	Italy	182	260	Mean 65·8±13·6	46·5	53·5	LightCycler SeptiFast Test MGRADE®	NA	Severe sepsis/septic shock	ICU	X
Delcò et al (2017)[81]	excluded contaminants	prospective cohort	Switzerland	114	114	Median in weeks: 32·43 (IQR 27·14-36·41)	58·0	42·0	IRIDICA BAC BSI assay	sample and patient	suspected sepsis	ICU	✓
Dinç et al (2016)[82]	only one BC bottle	prospective cohort	Turkey	67	78	Mean 55·7 (range 21-95)	36·0	64·0	LightCycler SeptiFast Test MGRADE®	sample	suspected or diagnosed sepsis/severe sepsis/septic shock	mixed (ICU and internal medicine wards)	X
Elges et al (2017)[83]	insufficient data	prospective cohort	Germany	104	847	Mean 51 (range 19-70)	45·2	54·8	LightCycler SeptiFast Test MGRADE®	NA	fever in patients with hematological malignancies	immunosuppression (bone marrow transplant unit)	✓
Gies et al (2016)[84]	only one BC bottle	prospective cohort	Germany	39	39	Median 5 (range 0·1-18)	59·0	41·0	LightCycler SeptiFast Test MGRADE®	sample and patient	suspected sepsis	ICU	X
Huber et al (2021)[85]	insufficient data	retrospective cohort	Austria	1528	2167	Mean 59·47±17·51	34·0	66·0	LightCycler SeptiFast Test MGRADE®	NA	Per clinical judgment	mixed (ICU units and general wards)	X
Kasper et al (2013)[86]	only one BC bottle	prospective cohort	Austria	46	NA	infants	NA	NA	LightCycler SeptiFast Test MGRADE®	patient	suspected sepsis	NA	✓

Lamoth et al (2010)[87]	unit of analysis: episodes	prospective cohort	Switzerland	86	141 episodes	Median 54 (range 17-71)	38·0	62·0	LightCycler SeptiFast Test MGRADE®	NA	febrile neutropenia (day 0 of fever onset)	immunosuppression (infectious diseases service)	✓
Lehmann et al (2009)[88]	unit of analysis: episodes	prospective cohort	Germany, Spain, Italy, USA	436	467 episodes	Mean 54·8 (range 18-92)	38·5	61·5	LightCycler SeptiFast Test MGRADE®	NA	suspected sepsis	mixed (hospitalized patients)	?
Ljungström et al (2015)[89]	excluded contaminants	prospective cohort	Sweden	375	383	adults	NA	NA	MagicPlex Sepsis Test	patient	suspected sepsis	ED	X
Louie et al (2008)[90]	excluded contaminants	prospective cohort	USA	200	NA	Median 47 (range 18-80) (male) Median 46 (range 18-91) (female)	39·0	61·0	LightCycler SeptiFast Test MGRADE®	patient	suspected sepsis	mixed (ED, medical ward and ICU)	✓
Matsushima et al (2012)[91]	insufficient data	prospective cohort	Japan	26	NA	Median 66 (range 39-91)	38·4	61·6	LightCycler SeptiFast Test MGRADE®	NA	diagnosed/suspected sepsis	ICU	X
Mencacci et al (2012)[92]	insufficient data	retrospective cohort	Italy	1009	1009	Median 69 (IQR 51-80)	45·0	55·0	LightCycler SeptiFast Test MGRADE®	NA	fever and suspected sepsis	mixed (medical wards, surgical wards, ICU)	X
Mihajlovic et al (2017)[93]	excluded contaminants	retrospective cohort	Serbia	100	100	Range 19-80	29·0	71·0	LightCycler SeptiFast Test MGRADE®	sample	severe sepsis/septic shock	ICU	X
Phung et al (2017)[94]	insufficient data	prospective cohort	Vietnam	57	57	Median 0·75 (range 0·085-9)	43·9	56·1	LightCycler SeptiFast Test MGRADE®	NA	infectious pneumonia	ICU	X
Pilarczyk et al (2019)[95]	excluded contaminants	retrospective cohort	Germany	169	279	NA	NA	NA	LightCycler SeptiFast Test MGRADE®	NA	suspected sepsis (in patients after cardiothoracic surgery)	ICU	X
Simms et al (2023)[96]	insufficient data	prospective cohort	Australia	203	360	Mean 58 (male) Mean 59 (female) Median 62 (range 12-94)	43·4	56·6	InfectID-BSI test	NA	suspected sepsis	ED	✓
Stein et al (2023)[97]	only one BC bottle	prospective cohort	Germany	229	229	Mean in weeks 35·3±3·9 (range in weeks 23·57-41·57)	NA	NA	LightCycler SeptiFast Test MGRADE®	NA	suspected sepsis and healthy newborns with perinatal risk factors	mixed	✓

Straub et al (2017)[98]	only one BC bottle	prospective, clinical trial	Austria	206	NA	Mean 0·545±0·007	46·6	53·4	LightCycler SeptiFast Test MGRADE®	sample	suspected sepsis	ICU	✓
Tassinari et al (2018)[99]	excluded contaminants	prospective cohort	Italy	300	300	adults	NA	NA	IRIDICA BAC BSI assay	sample	suspected sepsis	mixed (ED, infectious diseases unit)	✓
Tschiedel et al (2012)[100]	only one BC bottle	retrospective cohort	Germany	75	110	Median 6 (range 0-24)	50·7	49·3	LightCycler SeptiFast Test MGRADE®	sample	suspected sepsis	ICU	X
Vincent et al (2015)[101]	excluded contaminants	prospective cohort	Belgium, UK, Switzerland, France, Poland and Germany	543	616	Mean 61±17·9	35·4	64·6	IRIDICA BAC BSI assay	sample	suspected sepsis/proven sepsis/severe infection	ICU	✓
von Lilienfeld-Toal et al (2009)[102]	unit of analysis: episodes	prospective cohort	Germany	70	119 episodes	Median 60 (IQR 49-66)	46·0	54·0	LightCycler SeptiFast Test MGRADE®	NA	febrile neutropenia	immunosuppression (hematology ward)	✓
Warhurst et al (2014)[103]	unit of analysis: episodes	prospective cohort	England	795	922 episodes	Median 58 (IQR 44-68)	40·0	60·0	LightCycler SeptiFast Test MGRADE®	NA	suspected sepsis	ICU	X
Wu et al (2024)[104]	excluded contaminants	prospective cohort	China	89	100	Median 63 (IQR 48-71)	31·5	68·5	ddPCR	sample	suspected sepsis	ICU	X
Yanagihara et al (2010)[105]	excluded contaminants	prospective cohort	Japan	212	407	NA	35·0	65·0	LightCycler SeptiFast Test MGRADE®	sample	suspected sepsis	mixed (surgery, hematology, ED, cardiopulmonary and ICU)	✓

✓ indicates sponsorship by the manufacturer. X indicates no sponsorship by the manufacturer. ? indicates no information regarding the sponsorship by the manufacturer.
NA=not available. BC=blood culture. ED=emergency department. ICU=intensive care unit. IQR=interquartile range.

Table S4. Examining the difference between diagnostic measures of different devices. Different units of analysis are displayed separately.

	Sensitivity	p-value	Adjusted p-value*	Specificity	p-value	Adjusted p-value*
**LightCycler SeptiFast Test MGRADE® vs IRIDICA BAC BSI assay**						
Sample	0·678 vs 0·722	0·47	0·94	0·863 vs 0·875	0·69	1·0
Patient	0·645 vs 0·783	0·050	0·25	0·875 vs 0·890	0·65	1·0
**LightCycler SeptiFast Test MGRADE® vs MagicPlex Sepsis Test**						
Sample	0·678 vs 0·417	0·00043	0·0026	0·863 vs 0·813	0·27	1·0
Patient	0·645 vs 0·492	0·016	0·66	0·875 vs 0·767	0·010	0·064
**LightCycler SeptiFast Test MGRADE® vs SepsiTest**						
Sample	0·678 vs 0·473	0·25	0·76	0·863 vs 0·863	1·0	1·0
Patient	0·645 vs 0·755	0·57	1·0	0·875 vs 0·805	0·32	0·97
**IRIDICA BAC BSI assay vs MagicPlex Sepsis Test**						
Sample	0·722 vs 0·417	0·00058	0·0029	0·875 vs 0·813	0·23	1·0
Patient	0·783 vs 0·492	0·00050	0·0030	0·890 vs 0·767	0·025	0·13
**IRIDICA BAC BSI assay vs SepsiTest**						
Sample	0·722 vs 0·473	0·17	0·69	0·875 vs 0·863	0·86	1·0
Patient	0·783 vs 0·755	0·87	0·87	0·890 vs 0·805	0·26	1·0
**SepsiTest vs MagicPlex Sepsis Test**						
Sample	0·473 vs 0·417	0·77	0·77	0.863 vs 0·813	0·55	1·0
Patient	0·755 vs 0·492	0·21	0·64	0.805 vs 0·767	0·70	0·70
*Holm-Bonferroni method was performed.						

Table S5. Heterogeneities of diagnostic metrics across RMA type-based subgroups. Different units of analysis are displayed separately.

	Sensitivity I^2 (95% CI)	Specificity I^2 (95% CI)
**LightCycler SeptiFast Test MGRADE®**		
Sample	0·773 (0·698–0·829)	0·903 (0·878–0·922)
Patient	0·720 (0·594–0·807)	0·838 (0·777–0·882)
**IRIDICA BAC BSI assay**		
Sample	0·671 (0·217–0·862)	0·868 (0·735–0·934)
Patient	0·293 (0·00–0·926)	0·929 (0·826–0·971)
**SepsiTest**		
Sample	0·901 (0·798–0·952)	0·884 (0·755–0·945)
Patient	0·803 (0·574–0·909)	0·860 (0·718–0·931)
**MagicPlex Sepsis Test**		
Sample	0·905 (0·787–0·958)	0·972 (0·951–0·984)
Patient	0·600 (0·00–0·886)	0·927 (0·818–0·970)
CI=confidence interval.		

Table S6. Examining the difference between diagnostic measures of rapid molecular assays subgrouped by patient population. Different units of analysis are displayed separately.

	Sensitivity	p-value	Adjusted p-value*	Specificity	p-value	Adjusted p-value*
**Intensive care vs Emergency Department**						
Sample	0·652 vs 0·640	0·91	1·0	0·819 vs 0·898	0·036	0·18
Patient	0·591 vs 0·643	0·60	1·0	0·811 vs 0·892	0·070	0·42
**Intensive care vs Immunosuppressed**						
Sample	0·652 vs 0·688	0·70	1·0	0·819 vs 0·884	0·016	0·097
Patient	0·591 vs 0·834	0·16	0·93	0·811 vs 0·878	0·11	0·55
**Intensive care vs Mixed**						
Sample	0·652 vs 0·653	0·99	0·99	0·819 vs 0·869	0·041	0·16
Patient	0·591 vs 0·638	0·53	1·00	0·811 vs 0·861	0·28	0·84
**Emergency Department vs Immunosuppressed**						
Sample	0·640 vs 0·688	0·68	1·0	0·898 vs 0·884	0·66	0·66
Patient	0·643 vs 0·834	0·25	1·0	0·892 vs 0·878	0·65	0·65
**Emergency Department vs Mixed**						
Sample	0·640 vs 0·653	0·90	1·0	0·898 vs 0·869	0·36	1·0
Patient	0·643 vs 0·638	0·95	0·95	0·892 vs 0·861	0·26	1·0
**Immunosuppressed vs Mixed**						
Sample	0·688 vs 0·653	0·69	1·0	0·884 vs 0·869	0·49	0·99
Patient	0·834 vs 0·638	0·22	1·0	0·878 vs 0·861	0·44	0·87
*Holm-Bonferroni method was performed.						

Table S7. Examining the difference between diagnostic measures of sponsored vs not sponsored articles regarding the LightCycler SeptiFast Test MGRADE®. Articles reporting different units of analysis were analyzed separately.

	Sponsorship by the manufacturer	No sponsorship by the manufacturer	p-value
**Unit of analysis: sample**			
Sensitivity (95% CI)	0·629 (0·547–0·704)	0·694 (0·574– 0·793)	0·36
Specificity (95% CI)	0·861 (0·826–0·889)	0·856 (0·793–0·902)	0·87
**Unit of analysis: patient**			
Sensitivity (95% CI)	0·585 (0·469–0·692)	0·705 (0·562–0·816)	0·18
Specificity (95% CI)	0·888 (0·854–0·915)	0·826 (0·769–0·871)	0·031
CI=confidence interval.			

Table S8. Subgroup analysis based on the number of blood culture sets drawn

	Sensitivity (95% CI) at least 2 BC sets were drawn*	Sensitivity (95% CI) all articles	Specificity (95% CI) at least 2 BC sets were drawn*	Specificity (95% CI) all articles
**Unit of analysis: sample**				
All articles containing sample numbers	0·670 (0.599–0·734)	0·672 (0.615–0·725)	0·849 (0·814–0·879)	0·858 (0·835–0·879)
IRIDICA subgroup	0·722 (0·611–0·811)	0·722 (0.611–0·811)	0·875 (0·813–0·919)	0·875 (0·813–0·919)
Septifast subgroup	0·637 (0·541–0·724)	0·678 (0.615–0·734)	0·860 (0·815–0·895)	0·863 (0·837–0·886)
Intensive care subgroup	0·754 (0·600–0·862)	0·652 (0.547–0·744)	0·820 (0·741–0·879)	0·819 (0·774–0·857)
Emergency department subgroup	0·745 (0·587–0·858)	0·640 (0.454–0·791)	0·833 (0·724–0·905)	0·898 (0·835–0·939)
Immunosuppression subgroup	0·588 (0·457–0·708)	0·688 (0·525–0·814)	0·865 (0·798–0·913)	0·884 (0·847–0·913)
Mixed subgroup	0·643 (0·505–0·761)	0·653 (0·561–0·735)	0·869 (0·821–0·906)	0·869 (0·840–0·894)
**Unit of analysis: patient**				
All articles containing patient numbers	0·641 (0·556–0·718)	0·659 (0·594–0·719)	0·864 (0·825–0·896)	0·858 (0·830–0·883)
Septifast subgroup	0·598 (0·485–0·702)	0·645 (0·573–0·712)	0·882 (0·837–0·915)	0·875 (0·849–0·897)
Emergency department subgroup	0·689 (0·549–0·801)	0·643 (0·492–0·770)	0·837 (0·756–0·896)	0·892 (0·838–0·930)
Immunosuppression subgroup	0·609 (0·353–0·816)	0·834 (0·497–0·963)	0·879 (0·809–0·926)	0·878 (0·835–0·911)
Mixed subgroup	0·606 (0·490–0·711)	0·638 (0·561–0·709)	0·880 (0·828–0·918)	0·857 (0·817–0·890)

CI=confidence interval. BC=blood culture.
* one BC set=one aerobic and one anaerobic blood culture bottle

Table S9. Risk of bias assessment of included studies

Author (year)	Index test	QUADAS-2							QUADAS-C			
		P	I	R	FT	P	I	R	P	I	R	FT
Avolio et al (2014)	LightCycler SeptiFast Test MGRADE®	X	✓	X	✓	✓	✓	?	NA	NA	NA	NA
Avolio et al (2010)	LightCycler SeptiFast Test MGRADE®	✓	✓	X	?	✓	✓	?	NA	NA	NA	NA
Bacconi et al (2014)	IRIDICA BAC BSI	✓	✓	X	✓	✓	✓	✓	NA	NA	NA	NA
Bloos et al (2010)	VYOO	✓	✓	X	?	✓	?	?	NA	NA	NA	NA
Bloos et al (2012)	VYOO	✓	✓	X	?	✓	✓	?	NA	NA	NA	NA
Bravo et al (2011)	LightCycler SeptiFast Test MGRADE®	X	✓	X	✓	✓	✓	✓	NA	NA	NA	NA
Cambau et al (2017)	LightCycler SeptiFast Test MGRADE®	✓	✓	X	?	✓	✓	✓	NA	NA	NA	NA
Carrara et al (2013)	MagicPlex Sepsis Test	✓	✓	X	✓	✓	✓	?	NA	NA	NA	NA
Casalta et al (2009)	LightCycler SeptiFast Test MGRADE®	X	✓	X	✓	✓	?	?	NA	NA	NA	NA
Chaidaroglou et al (2013)	LightCycler SeptiFast Test MGRADE®	✓	✓	X	✓	✓	✓	?	NA	NA	NA	NA
Desmet et al (2016)	IRIDICA BAC BSI	✓	✓	X	✓	✓	✓	✓	NA	NA	NA	NA
Dierkes et al (2009)	LightCycler SeptiFast Test MGRADE®	?	✓	X	✓	✓	?	?	NA	NA	NA	NA
Fernández-Romero et al (2014)	LightCycler SeptiFast Test MGRADE®	✓	✓	X	✓	✓	?	?	NA	NA	NA	NA
Fitting et al (2012)	VYOO	✓	✓	X	?	✓	✓	?	NA	NA	NA	NA
Gosiewski et al (2014)	LightCycler SeptiFast Test MGRADE	✓	✓	X	?	✓	✓	?	NA	NA	NA	NA
Greco et al (2018)	LightCycler SeptiFast Test MGRADE®	✓	✓	X	✓	✓	✓	✓	NA	NA	NA	NA
Grijalva et al (2020)	LightCycler SeptiFast Test MGRADE®	✓	✓	X	?	✓	?	?	NA	NA	NA	NA
Grosse-Onnebrink et al (2017)	LightCycler SeptiFast Test MGRADE®	✓	✓	X	✓	✓	✓	?	NA	NA	NA	NA
Grosso et al (2021)	MicrobScan	✓	✓	X	?	✓	✓	✓	✓	✓	✓	✓
	MicrobScan-Kairos24/7	✓	✓	X	?	✓	X	✓				
Guido et al (2012)	LightCycler SeptiFast Test MGRADE®	✓	✓	X	?	✓	?	X	NA	NA	NA	NA
Haag et al (2013)	SepsiTest	✓	✓	X	?	✓	✓	?	NA	NA	NA	NA
Herne et al (2013)	LightCycler SeptiFast Test MGRADE®	✓	✓	X	X	✓	✓	✓	NA	NA	NA	NA

Hettwer et al (2012)	LightCycler SeptiFast Test MGRADE®	?	✓	X	✓	✓	✓	?	NA	NA	NA	NA
Idelevich et al (2015)	LightCycler SeptiFast Test MGRADE®	✓	✓	X	✓	✓	✓	X	NA	NA	NA	NA
Jordana-Lluch et al (2017)	IRIDICA BAC BSI	✓	✓	X	✓	✓	✓	✓	NA	NA	NA	NA
Jordana-Lluch et al (2015)	IRIDICA BAC BSI	✓	✓	X	✓	✓	✓	✓	NA	NA	NA	NA
Josefson et al (2011)	LightCycler SeptiFast Test MGRADE®	✓	✓	X	✓	✓	✓	✓	NA	NA	NA	NA
Khün et al (2011)	SepsiTest	?	✓	X	✓	✓	✓	?	NA	NA	NA	NA
Kim et al (2020)	REBA Sepsis-ID	✓	✓	X	?	✓	✓	✓	NA	NA	NA	NA
Korber et al (2017)	LightCycler SeptiFast Test MGRADE®	✓	✓	X	X	✓	X	?	NA	NA	NA	NA
Lehmann et al (2010)	LightCycler SeptiFast Test MGRADE®	✓	✓	X	✓	✓	✓	?	NA	NA	NA	NA
Leli et al (2014)/a	LightCycler SeptiFast Test MGRADE®	✓	✓	X	✓	✓	✓	✓	NA	NA	NA	NA
Leli et al (2014) /b	LightCycler SeptiFast Test MGRADE®	✓	✓	X	✓	✓	✓	✓	NA	NA	NA	NA
Liotti et al (2019)	MicrobScan	✓	✓	X	✓	✓	✓	?	NA	NA	NA	NA
Lodes et al (2012)	LightCycler SeptiFast Test MGRADE®	✓	✓	X	✓	✓	✓	?	NA	NA	NA	NA
Loonen et al (2014)	SepsiTest	✓	✓	X	✓	✓	✓	✓	✓	✓	✓	✓
	MagicPlex Sepsis Test	✓	✓	X	✓	✓	✓	✓				
Lucignano et al (2011)	LightCycler SeptiFast Test MGRADE®	✓	✓	X	✓	✓	✓	?	NA	NA	NA	NA
Mahmoud et al (2023)	LightCycler SeptiFast Test MGRADE®	✓	✓	X	?	✓	?	?	NA	NA	NA	NA
Makristathis et al (2018)	IRIDICA BAC BSI	✓	✓	X	✓	✓	✓	✓	✓	✓	✓	✓
	LightCycler SeptiFast Test MGRADE®	✓	✓	X	✓	✓	✓	✓				
Mancini et al (2008)	LightCycler SeptiFast Test MGRADE®	✓	✓	X	✓	✓	✓	?	NA	NA	NA	NA
Markota et al (2014)	LightCycler SeptiFast Test MGRADE®	✓	✓	X	✓	✓	?	?	NA	NA	NA	NA
Maubon et al (2010)	LightCycler SeptiFast Test MGRADE®	✓	✓	X	✓	✓	✓	✓	NA	NA	NA	NA
Mauro et al (2012)	LightCycler SeptiFast Test MGRADE®	✓	✓	X	✓	✓	✓	✓	NA	NA	NA	NA
Metzgar et al (2016)	IRIDICA BAC BSI	✓	✓	X	?	✓	✓	✓	NA	NA	NA	NA
Mongelli et al (2015)	LightCycler SeptiFast Test MGRADE®	✓	✓	X	✓	✓	✓	✓	NA	NA	NA	NA

Nieman et al (2016)	SepsiTest	✓	✓	X	✓	✓	X	?	NA	NA	NA	NA
Obara et al.(2011)	LightCycler SeptiFast Test MGRADE®	✓	✓	X	✓	✓	✓	?	NA	NA	NA	NA
Ortiz Ibarra et al (2015)	LightCycler SeptiFast Test MGRADE®	X	✓	X	✓	✓	✓	?	NA	NA	NA	NA
Ozkaya-Parlakay et al (2014)	LightCycler SeptiFast Test MGRADE®	✓	✓	X	✓	✓	?	?	NA	NA	NA	NA
Paolucci et al (2013)	LightCycler SeptiFast Test MGRADE®	✓	✓	X	✓	✓	✓	?	NA	NA	NA	NA
Pasqualini et al (2012)	LightCycler SeptiFast Test MGRADE®	✓	✓	X	✓	✓	✓	?	NA	NA	NA	NA
Rath et al (2012)	LightCycler SeptiFast Test MGRADE®	✓	✓	X	✓	✓	✓	✓	NA	NA	NA	NA
Ratzinger et al (2016)	LightCycler SeptiFast Test MGRADE®	X	✓	X	X	✓	✓	?	NA	NA	NA	NA
Regueiro et al (2010)	LightCycler SeptiFast Test MGRADE®	✓	✓	X	?	✓	X	?	NA	NA	NA	NA
Reyna-Figueroa et al (2019)	LightCycler SeptiFast Test MGRADE®	X	✓	X	?	✓	?	?	NA	NA	NA	NA
Rodrigues et al (2019)	LightCycler SeptiFast Test MGRADE®	✓	✓	X	?	✓	?	?	NA	NA	NA	NA
Rogina et al (2014)	SepsiTest	X	✓	X	?	✓	✓	✓	NA	NA	NA	NA
Schaub et al (2014)	LightCycler SeptiFast Test MGRADE®	✓	✓	X	✓	✓	✓	X	NA	NA	NA	NA
Schreiber et al (2013)	LightCycler SeptiFast Test MGRADE®	✓	✓	X	?	✓	✓	?	✓	✓	✓	?
	SepsiTest	✓	✓	X	?	✓	✓	?				
	VYOO	✓	✓	X	?	✓	✓	?				
Sitnik et al (2014)	LightCycler SeptiFast Test MGRADE	✓	✓	X	✓	✓	✓	?	NA	NA	NA	NA
Strålin et al (2020)	IRIDICA BAC BSI	X	✓	X	✓	✓	✓	?	NA	NA	NA	NA
Suberviola et al (2016)	LightCycler SeptiFast Test MGRADE®	✓	✓	X	✓	✓	✓	?	NA	NA	NA	NA
Tafelski et al (2015)	LightCycler SeptiFast Test MGRADE®	✓	✓	X	✓	✓	✓	✓	NA	NA	NA	NA
Tat Trung et al (2018)	LightCycler SeptiFast Test MGRADE®	✓	✓	X	✓	✓	?	X	NA	NA	NA	NA
Tkadlec et al (2019)	UMD-SelectNA	✓	✓	X	?	✓	✓	?	NA	NA	NA	NA
Tran et al (2012)	LightCycler SeptiFast Test MGRADE®	X	✓	X	✓	✓	?	?	NA	NA	NA	NA

Tröger et al (2016)	LightCycler SeptiFast Test MGRADE®	X	✓	X	?	✓	?	?	NA	NA	NA	NA
Tsalik et al (2010)	LightCycler SeptiFast Test MGRADE®	✓	✓	X	?	✓	✓	?	NA	NA	NA	NA
Varani et al (2009)	LightCycler SeptiFast Test MGRADE®	✓	✓	X	✓	✓	✓	?	NA	NA	NA	NA
Wallet et al (2009)	LightCycler SeptiFast Test MGRADE®	✓	✓	X	✓	✓	✓	X	NA	NA	NA	NA
Wang et al (2015)	REBA Sepsis-ID	✓	✓	X	✓	✓	✓	✓	NA	NA	NA	NA
Wellinghausen et al (2009)	SepsiTest	✓	✓	X	✓	✓	✓	?	NA	NA	NA	NA
Westh et al (2009)	LightCycler SeptiFast Test MGRADE®	?	✓	X	✓	✓	X	✓	NA	NA	NA	NA
Zboromyrska et al (2019)	MagicPlex Sepsis Test	X	✓	X	✓	✓	✓	?	NA	NA	NA	NA
Ziegler et al (2016)	MagicPlex Sepsis Test	✓	✓	X	✓	✓	✓	✓	NA	NA	NA	NA

P=patient selection. I=index test. R=reference standard. FT=flow and timing. NA=not applicable. ✓ indicates low risk. X indicates high risk. ? indicates unclear risk.

Supplementary Figures

Figure S1. Pooled sensitivity and specificity of rapid molecular assays for the diagnosis of bloodstream infections when compared to blood culture. Unit of analysis: patient.

Study	TP	BC+	TN	BC−	Sensitivity [95% CI]	Specificity [95% CI]
Avolio et al (2010)	30	43	91	101	0.698 [0.539–0.828]	0.901 [0.825–0.951]
Avolio et al (2014)	83	129	356	396	0.643 [0.554–0.726]	0.899 [0.865–0.927]
Bacconi at al (2014)	16	19	293	312	0.842 [0.604–0.966]	0.939 [0.907–0.963]
Bravo et al (2011))	12	25	24	28	0.480 [0.278–0.687]	0.857 [0.673–0.960]
Cambauet al (2017)	83	164	446	567	0.506 [0.427–0.585]	0.787 [0.751–0.820]
Carrara et al (2013)	29	69	169	198	0.420 [0.302–0.545]	0.854 [0.796–0.900]
Casalta et al (2009)	8	19	41	44	0.421 [0.203–0.665]	0.932 [0.813–0.986]
Dierkes et al (2009)	14	21	48	56	0.667 [0.430–0.854]	0.857 [0.738–0.936]
Fitting et al (2012)	14	20	41	66	0.700 [0.457–0.881]	0.621 [0.493–0.738]
Grijalva et al (2020)	34	56	158	199	0.607 [0.468–0.735]	0.794 [0.731–0.848]
Grosso et al (2021)	61	87	192	220	0.701 [0.594–0.795]	0.873 [0.821–0.914]
Guido et al (2012)	21	23	126	143	0.913 [0.720–0.989]	0.881 [0.816–0.929]
Haag et al (2013)	3	3	5	9	1.000 [0.292–1.000]	0.556 [0.212–0.863]
Herne et al (2013)	27	34	83	110	0.794 [0.621–0.913]	0.755 [0.663–0.832]
Hettwer et al (2012)	39	51	57	62	0.765 [0.625–0.872]	0.919 [0.822–0.973]
Josefson et al (2011)	50	146	883	947	0.342 [0.266–0.425]	0.932 [0.915–0.948]
Kim et al (2020)	30	61	336	379	0.492 [0.361–0.623]	0.887 [0.850–0.917]
Kühn et al (2011)	2	3	11	25	0.667 [0.094–0.992]	0.440 [0.244–0.651]
Leli et al (2014)/a	61	104	437	467	0.587 [0.486–0.682]	0.936 [0.910–0.956]
Leli et al (2014)/b	25	40	220	245	0.625 [0.458–0.773]	0.898 [0.853–0.933]
Loonen et al (2014)	3	27	94	98	0.111 [0.024–0.292]	0.959 [0.899–0.989]
Mahmoud et al (2023)	34	34	22	26	1.000 [0.897–1.000]	0.846 [0.651–0.956]
Maubon et al (2010)	18	32	67	78	0.562 [0.377–0.736]	0.859 [0.762–0.927]
Mauro et al (2012)	27	32	42	47	0.844 [0.672–0.947]	0.894 [0.769–0.965]
Metzgar et al (2016)	32	40	207	233	0.800 [0.644–0.909]	0.888 [0.841–0.926]
Oritz Ibarra et al (2015)	9	13	48	73	0.692 [0.386–0.909]	0.658 [0.537–0.765]
Pasqualini et al (2012)	34	76	289	315	0.447 [0.333–0.566]	0.917 [0.881–0.945]
Ratzinger et al (2016)	31	46	156	174	0.674 [0.520–0.805]	0.897 [0.841–0.938]
Reyna Figueroa et al (2019)	4	4	12	16	1.000 [0.398–1.000]	0.750 [0.476–0.927]
Rodrigues et al (2019)	28	39	144	161	0.718 [0.551–0.850]	0.894 [0.836–0.937]
Rogina et al (2014)	3	3	14	20	1.000 [0.292–1.000]	0.700 [0.457–0.881]
Schaub et al (2014)	16	27	78	83	0.593 [0.388–0.776]	0.940 [0.865–0.980]
Schreiber et al (2013)	3	13	33	37	0.231 [0.050–0.538]	0.892 [0.746–0.970]
Sitnik et al (2014)	13	17	87	97	0.765 [0.501–0.932]	0.897 [0.819–0.949]
Stralin et al (2020)	150	213	1023	1247	0.704 [0.638–0.765]	0.820 [0.798–0.841]
Tafelski et al (2015)	7	12	55	66	0.583 [0.277–0.848]	0.833 [0.721–0.914]
Tat Trung et al (2018)	19	35	63	75	0.543 [0.366–0.712]	0.840 [0.737–0.914]
Tran et al (2012)	13	16	23	34	0.812 [0.544–0.960]	0.676 [0.495–0.826]
Tsalik et al (2010)	40	92	201	214	0.435 [0.332–0.542]	0.939 [0.898–0.967]
Wallet et al (2009)	5	11	53	61	0.455 [0.167–0.766]	0.869 [0.758–0.942]
Wang et al (2015)	115	142	617	740	0.810 [0.736–0.871]	0.834 [0.805–0.860]
Wellinghausen et al (2009)	28	34	122	153	0.824 [0.655–0.932]	0.797 [0.725–0.858]
Ziegler et al (2016)	74	128	374	568	0.578 [0.488–0.665]	0.658 [0.618–0.697]
					0.659 [0.594; 0.719]	0.858 [0.830; 0.883]
Heterogeneity:					I^2 = 78% [71%–83%] p < 0.01	I^2 = 90% [88%–92%] p < 0.01

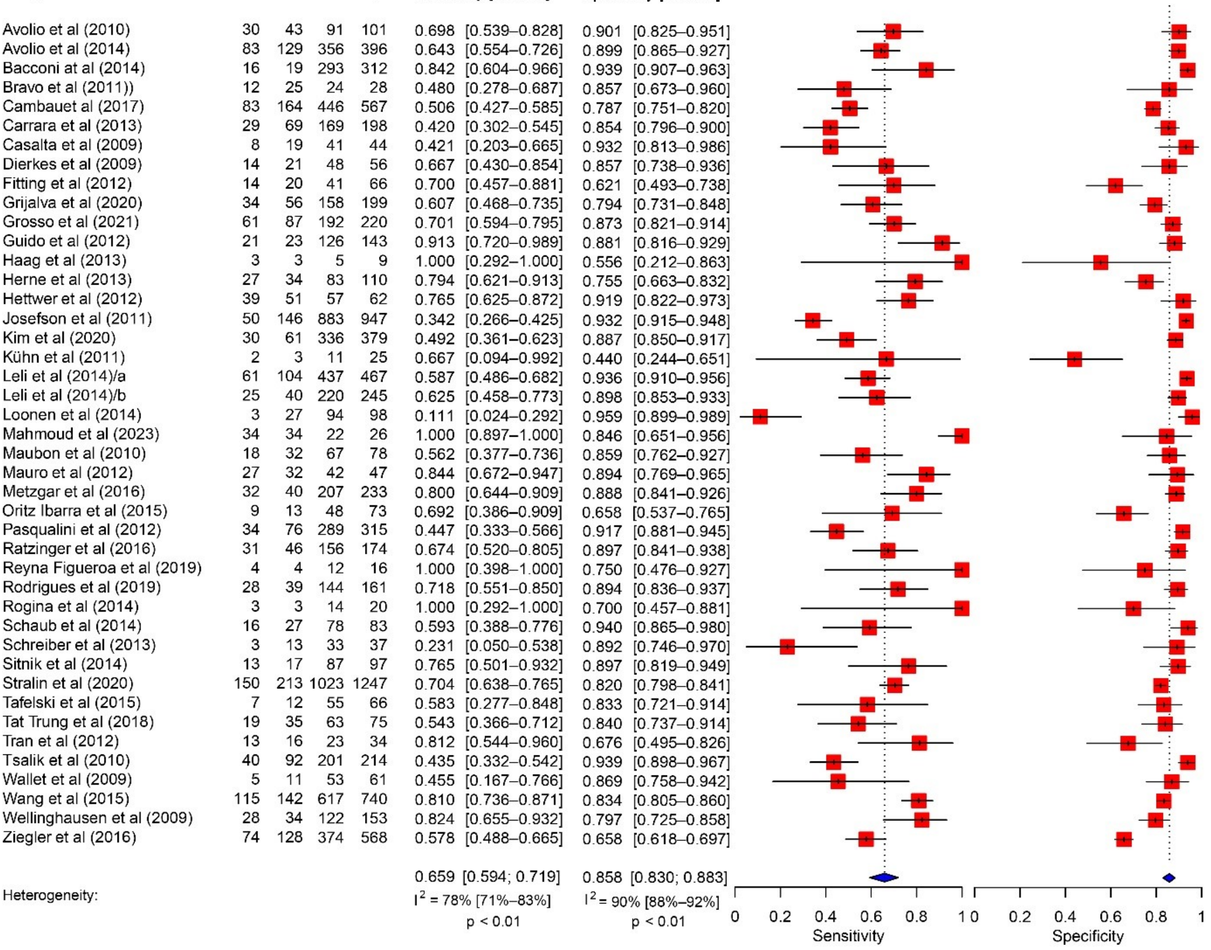

Abbreviations: TP=true positive. BC+=blood culture positive. TN=true negative. BC-=blood culture negative. CI=confidence interval.

Figure S2. Bivariate summary estimates of sensitivity and specificity for rapid molecular assays (RMA) when compared to blood culture with 95% confidence (dashed) and 95% prediction ellipses (dotted). Filled squares represent pooled diagnostic metric estimates. Different units of analysis are displayed separately.

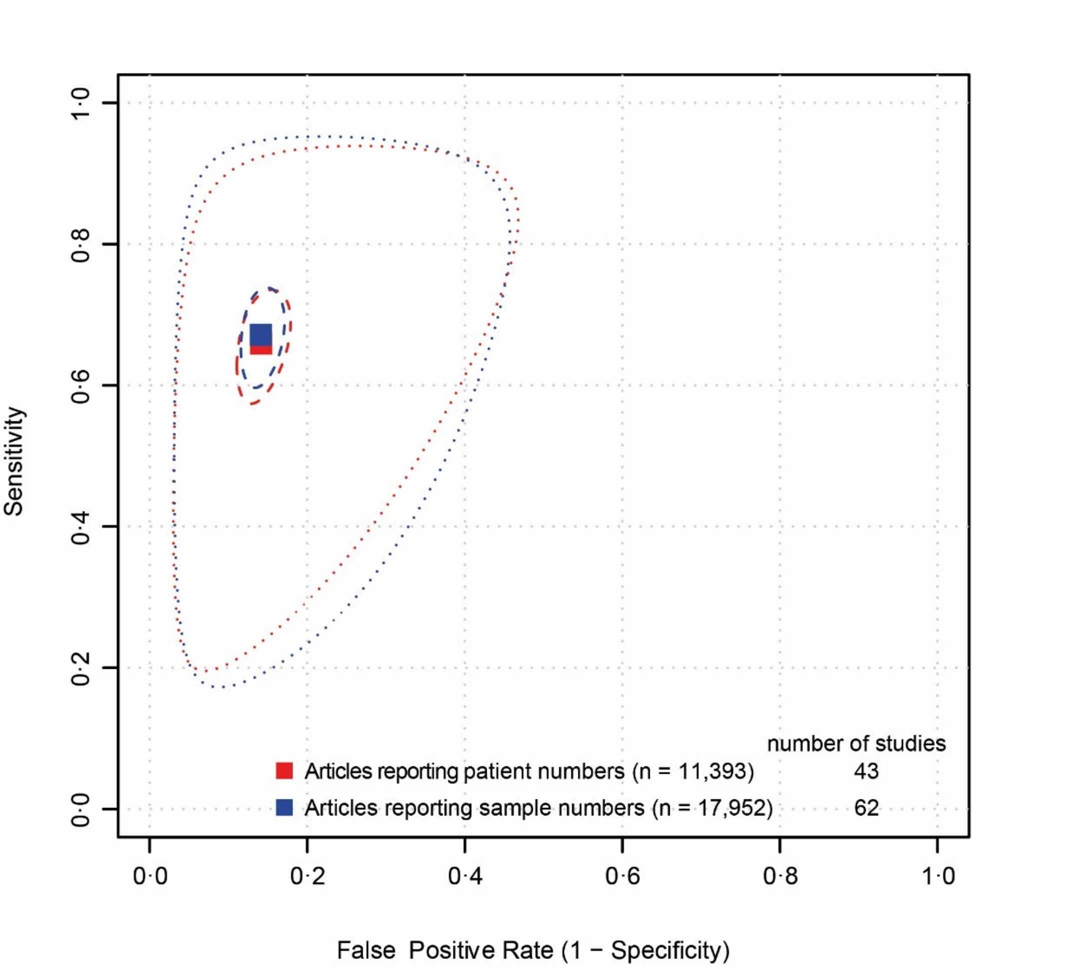

Figure S3. Age category-based subgroup analysis in the LightCycler SeptiFast Test MGRADE® subgroup. A) Articles with adult population. B) Articles with infant, newborn or both categories. Unit of analysis: sample.

a)

Study	TP	BC+	TN	BC−	Sensitivity [95% CI]	Specificity [95% CI]
Avolio, M.et al., 2010	30	43	91	101	0.698 [0.539; 0.828]	0.901 [0.825; 0.951]
Bloos, F. et al., 2010	31	39	146	197	0.795 [0.635; 0.907]	0.741 [0.674; 0.801]
Bravo, D. et al., 2011	21	50	32	36	0.420 [0.282; 0.568]	0.889 [0.739; 0.969]
Chaidaroglou, A.et al., 2013	5	8	113	122	0.625 [0.245; 0.915]	0.926 [0.865; 0.966]
Dierkes, C.et al., 2009	17	26	63	75	0.654 [0.443; 0.828]	0.840 [0.737; 0.914]
Fernández−Romero, N. et al., 2014	15	21	66	75	0.714 [0.478; 0.887]	0.880 [0.784; 0.944]
Gosiewski, T. et al., 2014	10	26	37	45	0.385 [0.202; 0.594]	0.822 [0.679; 0.920]
Guido, M. et al., 2012	21	23	126	143	0.913 [0.720; 0.989]	0.881 [0.816; 0.929]
Herne, V. et al., 2013	27	34	99	126	0.794 [0.621; 0.913]	0.786 [0.704; 0.854]
Hettwer, S. et al., 2012	39	51	57	62	0.765 [0.625; 0.872]	0.919 [0.822; 0.973]
Lehmann, L. E. et al., 2010	40	58	321	395	0.690 [0.555; 0.805]	0.813 [0.771; 0.850]
Lodes, U. et al., 2012	25	30	84	118	0.833 [0.653; 0.944]	0.712 [0.621; 0.792]
Makristathis, A. et al., 2018	31	51	131	142	0.608 [0.461; 0.742]	0.923 [0.866; 0.961]
Mancini, N. et al., 2008	20	21	68	82	0.952 [0.762; 0.999]	0.829 [0.730; 0.903]
Markota, A. et al., 2014	8	9	51	54	0.889 [0.518; 0.997]	0.944 [0.846; 0.988]
Mongelli, G. et al., 2015	25	30	88	108	0.833 [0.653; 0.944]	0.815 [0.729; 0.883]
Obara, H. et al., 2011	11	12	56	66	0.917 [0.615; 0.998]	0.848 [0.739; 0.925]
Pasqualini, L. et al., 2012	34	76	289	315	0.447 [0.333; 0.566]	0.917 [0.881; 0.945]
Rath, P. M. et al., 2012	57	80	110	145	0.713 [0.600; 0.808]	0.759 [0.681; 0.826]
Regueiro, B. J. et al., 2010	16	27	78	79	0.593 [0.388; 0.776]	0.987 [0.931; 1.000]
Regueiro, B. J. et al., 2010	25	27	77	79	0.926 [0.757; 0.991]	0.975 [0.912; 0.997]
Tsalik, E. L. et al., 2010	40	92	201	214	0.435 [0.332; 0.542]	0.939 [0.898; 0.967]
					0.717 [0.637; 0.786]	0.879 [0.844; 0.906]
					Heterogeneity: I^2 = 77% [65%; 85%] p < 0.01	I^2 = 81% [72%; 87%], p < 0.01

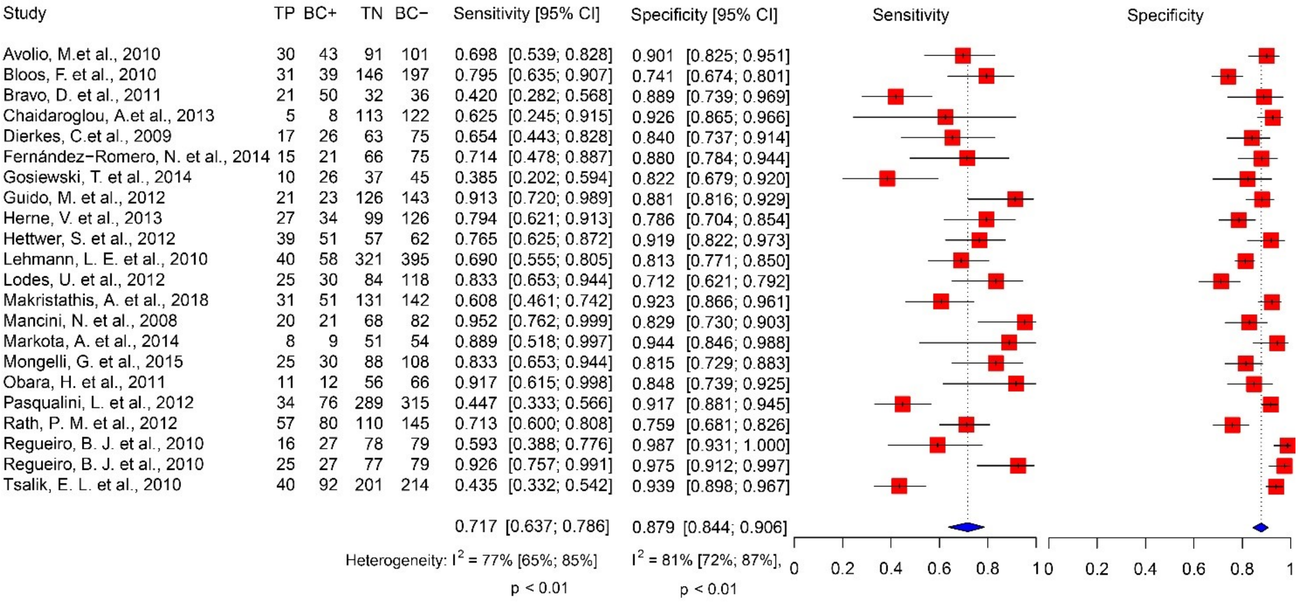

b)

Study	TP	BC+	TN	BC−	Sensitivity [95% CI]	Specificity [95% CI]
Oritz Ibarra, J. et al., 2015	9	13	48	73	0.692 [0.386; 0.909]	0.658 [0.537; 0.765]
Reyna Figueroa, J. et al., 2019	4	4	12	16	1.000 [0.398; 1.000]	0.750 [0.476; 0.927]
Tröger, B. et al., 2016	45	55	94	159	0.818 [0.691; 0.909]	0.591 [0.511; 0.668]
					0.806 [0.698; 0.881]	0.621 [0.559; 0.679]
					Heterogeneity: I^2 = 0% [0%; 90%] p = 0.61	I^2 = 5% [0%; 90%] p = 0.35

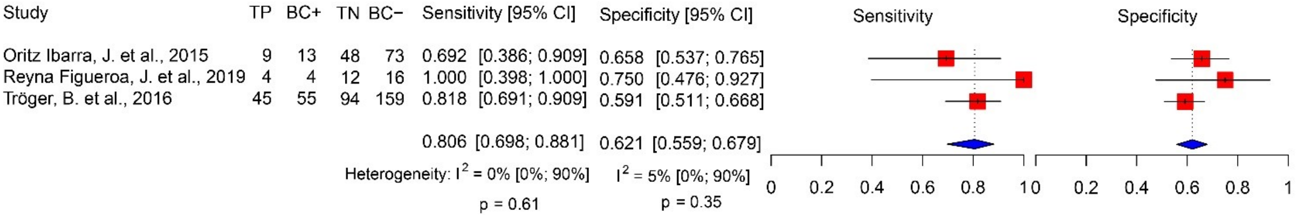

Abbreviations: TP=true positive. BC+=blood culture positive. TN=true negative. BC-=blood culture negative. CI=confidence interval.

Figure S4. Age category-based subgroup analysis in the LightCycler SeptiFast Test MGRADE® subgroup. Age category: adult population. Unit of analysis: patient.

Study	TP	BC+	TN	BC−	Sensitivity [95% CI]	Specificity [95% CI]
Avolio, M.et al., 2010	30	43	91	101	0.698 [0.539; 0.828]	0.901 [0.825; 0.951]
Avolio, M.et al., 2014	83	129	356	396	0.643 [0.554; 0.726]	0.899 [0.865; 0.927]
Bravo, D.et al., 2011	21	50	32	36	0.420 [0.282; 0.568]	0.889 [0.739; 0.969]
Cambau, E.et al., 2017	83	164	446	567	0.506 [0.427; 0.585]	0.787 [0.751; 0.820]
Dierkes, C.et al., 2009	14	21	48	56	0.667 [0.430; 0.854]	0.857 [0.738; 0.936]
Guido, M.et al., 2012	21	23	126	143	0.913 [0.720; 0.989]	0.881 [0.816; 0.929]
Herne, V.et al., 2013	27	34	83	110	0.794 [0.621; 0.913]	0.755 [0.663; 0.832]
Hettwer, S.et al., 2012	39	51	57	62	0.765 [0.625; 0.872]	0.919 [0.822; 0.973]
Pasqualini, L.et al., 2012	34	76	289	315	0.447 [0.333; 0.566]	0.917 [0.881; 0.945]
Ratzinger, F.et al., 2016	31	46	156	174	0.674 [0.520; 0.805]	0.897 [0.841; 0.938]
Rodrigues, C.et al., 2019	28	39	144	161	0.718 [0.551; 0.850]	0.894 [0.836; 0.937]
Tsalik, E. L.et al., 2010	40	92	201	214	0.435 [0.332; 0.542]	0.939 [0.898; 0.967]
					0.639 [0.546; 0.722]	0.884 [0.852; 0.910]
				Heterogeneity:	$I^2 = 79\%$ [63%; 88%] $p < 0.01$	$I^2 = 83\%$ [72%; 90%] $p < 0.01$

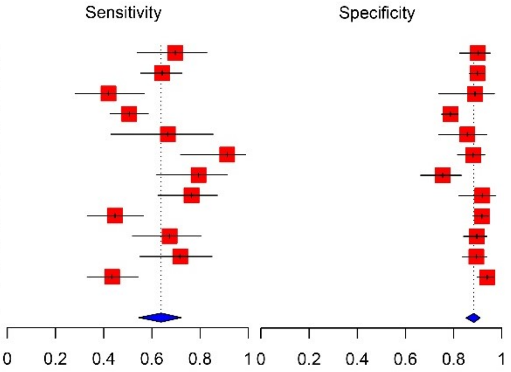

Abbreviations: TP=true positive. BC+=blood culture positive. TN=true negative. BC-=blood culture negative. CI=confidence interval.

Figure S5. Subgroup analysis of articles that properly reported contaminants in the LightCycler SeptiFast Test MGRADE® subgroup.
A) Unit of analysis: sample. B) Unit of analysis: patient.

a)

Study	TP	BC+	TN	BC−	Sensitivity [95% CI]	Specificity [95% CI]
Avolio, M. et al., 2010	30	43	91	101	0.698 [0.539; 0.828]	0.901 [0.825; 0.951]
Bravo, D. et al., 2011	21	50	32	36	0.420 [0.282; 0.568]	0.889 [0.739; 0.969]
Chaidaroglou, A. et al., 2013	5	8	113	122	0.625 [0.245; 0.915]	0.926 [0.865; 0.966]
Dierkes, C. et al., 2009	17	26	63	75	0.654 [0.443; 0.828]	0.840 [0.737; 0.914]
Gosiewski, T. et al., 2014	10	26	37	45	0.385 [0.202; 0.594]	0.822 [0.679; 0.920]
Greco, R. et al., 2018	174	317	1174	1520	0.549 [0.492; 0.605]	0.772 [0.750; 0.793]
Grosse−Onnebrink, J. et al., 2017	2	7	57	65	0.286 [0.037; 0.710]	0.877 [0.772; 0.945]
Oritz Ibarra, J. et al., 2015	9	13	48	73	0.692 [0.386; 0.909]	0.658 [0.537; 0.765]
Idelevich, E. et al., 2015	46	91	141	162	0.505 [0.399; 0.612]	0.870 [0.809; 0.918]
Josefson, P. et al., 2011	50	146	883	947	0.342 [0.266; 0.425]	0.932 [0.915; 0.948]
Korber, F. et al., 2017	49	71	323	399	0.690 [0.569; 0.795]	0.810 [0.767; 0.847]
Lehmann, L. E. et al., 2010	40	58	321	395	0.690 [0.555; 0.805]	0.813 [0.771; 0.850]
Leli, C. et al., 2014/b	25	40	220	245	0.625 [0.458; 0.773]	0.898 [0.853; 0.933]
Lodes, U. et al., 2012	25	30	84	118	0.833 [0.653; 0.944]	0.712 [0.621; 0.792]
Lucignano, B. et al., 2011	142	257	1303	1416	0.553 [0.489; 0.614]	0.920 [0.905; 0.934]
Makristathis, A. et al., 2018	31	51	131	142	0.608 [0.461; 0.742]	0.923 [0.866; 0.961]
Mancini, N. et al., 2008	20	21	68	82	0.952 [0.762; 0.999]	0.829 [0.730; 0.903]
Maubon, D. et al., 2010	18	32	67	78	0.562 [0.377; 0.736]	0.859 [0.762; 0.927]
Mongelli, G. et al., 2015	25	30	88	108	0.833 [0.653; 0.944]	0.815 [0.729; 0.883]
Paolucci, M. et al., 2013	25	63	262	274	0.397 [0.276; 0.528]	0.956 [0.925; 0.977]
Pasqualini, L. et al., 2012	34	76	289	315	0.447 [0.333; 0.566]	0.917 [0.881; 0.945]
Rath, P. M. et al., 2012	57	80	110	145	0.713 [0.600; 0.808]	0.759 [0.681; 0.826]
Regueiro, B. J. et al., 2010	16	27	78	79	0.593 [0.388; 0.776]	0.987 [0.931; 1.000]
Regueiro, B. J. et al., 2010	25	27	77	79	0.926 [0.757; 0.991]	0.975 [0.912; 0.997]
Reyna Figueroa, J. et al., 2019	4	4	12	16	1.000 [0.398; 1.000]	0.750 [0.476; 0.927]
Schreiber, J. et al., 2013	3	13	33	37	0.231 [0.050; 0.538]	0.892 [0.746; 0.970]
Sitnik, R. et al., 2014	13	17	87	97	0.765 [0.501; 0.932]	0.897 [0.819; 0.949]
Suberviola, B. et al., 2016	30	37	55	82	0.811 [0.648; 0.920]	0.671 [0.558; 0.771]
Tafelski, S. et al., 2015	7	12	55	66	0.583 [0.277; 0.848]	0.833 [0.721; 0.914]
Tröger, B. et al., 2016	45	55	94	159	0.818 [0.691; 0.909]	0.591 [0.511; 0.668]
Tsalik, E. L. et al., 2010	40	92	201	214	0.435 [0.332; 0.542]	0.939 [0.898; 0.967]
Varani, S. et al., 2009	22	40	101	114	0.550 [0.385; 0.707]	0.886 [0.813; 0.938]
Wallet, F. et al., 2010	4	10	79	90	0.400 [0.122; 0.738]	0.878 [0.792; 0.937]
Westh, H. et al., 2009	51	96	382	517	0.531 [0.427; 0.634]	0.739 [0.699; 0.776]
					0.613 [0.551; 0.672]	0.865 [0.833; 0.892]
Heterogeneity:					I^2 = 76% [67%; 83%] $p < 0.01$	I^2 = 92% [90%; 94%] $p < 0.01$

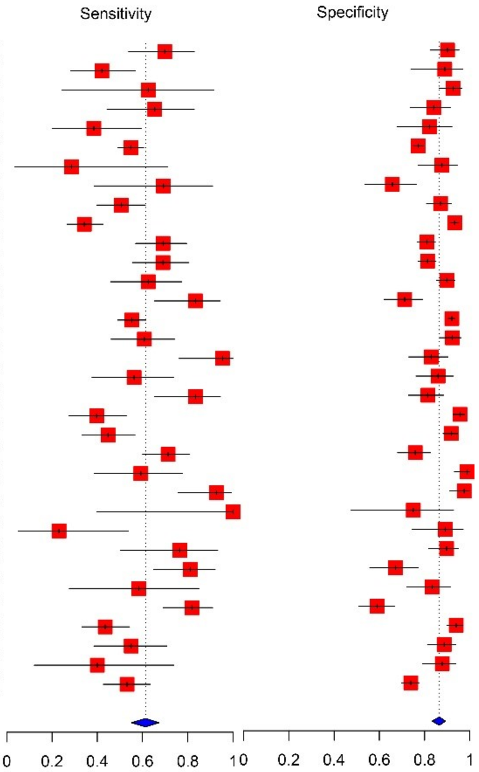

b)

Study	TP	BC+	TN	BC−	Sensitivity [95% CI]	Specificity [95% CI]
Avolio, M. et al., 2010	30	43	91	101	0.698 [0.539; 0.828]	0.901 [0.825; 0.951]
Avolio, M. et al., 2014	83	129	356	396	0.643 [0.554; 0.726]	0.899 [0.865; 0.927]
Bravo, D. et al., 2011	21	50	32	36	0.420 [0.282; 0.568]	0.889 [0.739; 0.969]
Cambau, E. et al., 2017	83	164	446	567	0.506 [0.427; 0.585]	0.787 [0.751; 0.820]
Dierkes, C. et al., 2009	14	21	48	56	0.667 [0.430; 0.854]	0.857 [0.738; 0.936]
Oritz Ibarra, J. et al., 2015	9	13	48	73	0.692 [0.386; 0.909]	0.658 [0.537; 0.765]
Josefson, P. et al., 2011	50	146	883	947	0.342 [0.266; 0.425]	0.932 [0.915; 0.948]
Leli, C. et al., 2014	61	104	437	467	0.587 [0.486; 0.682]	0.936 [0.910; 0.956]
Leli, C. et al., 2014/b	25	40	220	245	0.625 [0.458; 0.773]	0.898 [0.853; 0.933]
Maubon, D. et al., 2010	18	32	67	78	0.562 [0.377; 0.736]	0.859 [0.762; 0.927]
Pasqualini, L. et al., 2012	34	76	289	315	0.447 [0.333; 0.566]	0.917 [0.881; 0.945]
Ratzinger, F. et al., 2016	31	46	156	174	0.674 [0.520; 0.805]	0.897 [0.841; 0.938]
Reyna Figueroa, J. et al., 2019	4	4	12	16	1.000 [0.398; 1.000]	0.750 [0.476; 0.927]
Schaub, N. et al., 2014	16	27	78	83	0.593 [0.388; 0.776]	0.940 [0.865; 0.980]
Schreiber, J. et al., 2013	3	13	33	37	0.231 [0.050; 0.538]	0.892 [0.746; 0.970]
Sitnik, R. et al., 2014	13	17	87	97	0.765 [0.501; 0.932]	0.897 [0.819; 0.949]
Tafelski, S. et al., 2015	7	12	55	66	0.583 [0.277; 0.848]	0.833 [0.721; 0.914]
Tsalik, E. L. et al., 2010	40	92	201	214	0.435 [0.332; 0.542]	0.939 [0.898; 0.967]
Wallet, F. et al., 2010	5	11	53	61	0.455 [0.167; 0.766]	0.869 [0.758; 0.942]
					0.559 [0.495; 0.622]	0.888 [0.859; 0.911]
Heterogeneity:					I^2 = 68% [49%; 80%] $p < 0.01$	I^2 = 86% [80%; 91%] $p < 0.01$

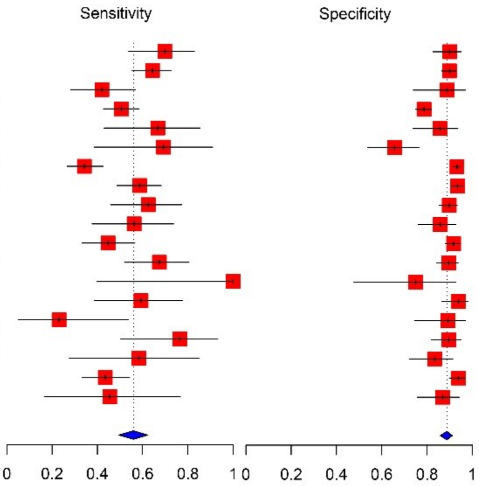

Abbreviations: TP=true positive. BC+=blood culture positive. TN=true negative. BC-=blood culture negative. CI=confidence interval.

Figure S6. Subgroup analysis of articles that either did not report contaminants properly or did not report them all in the LightCycler SeptiFast Test MGRADE® subgroup.
A) Unit of analysis: sample. B) Unit of analysis: patient.

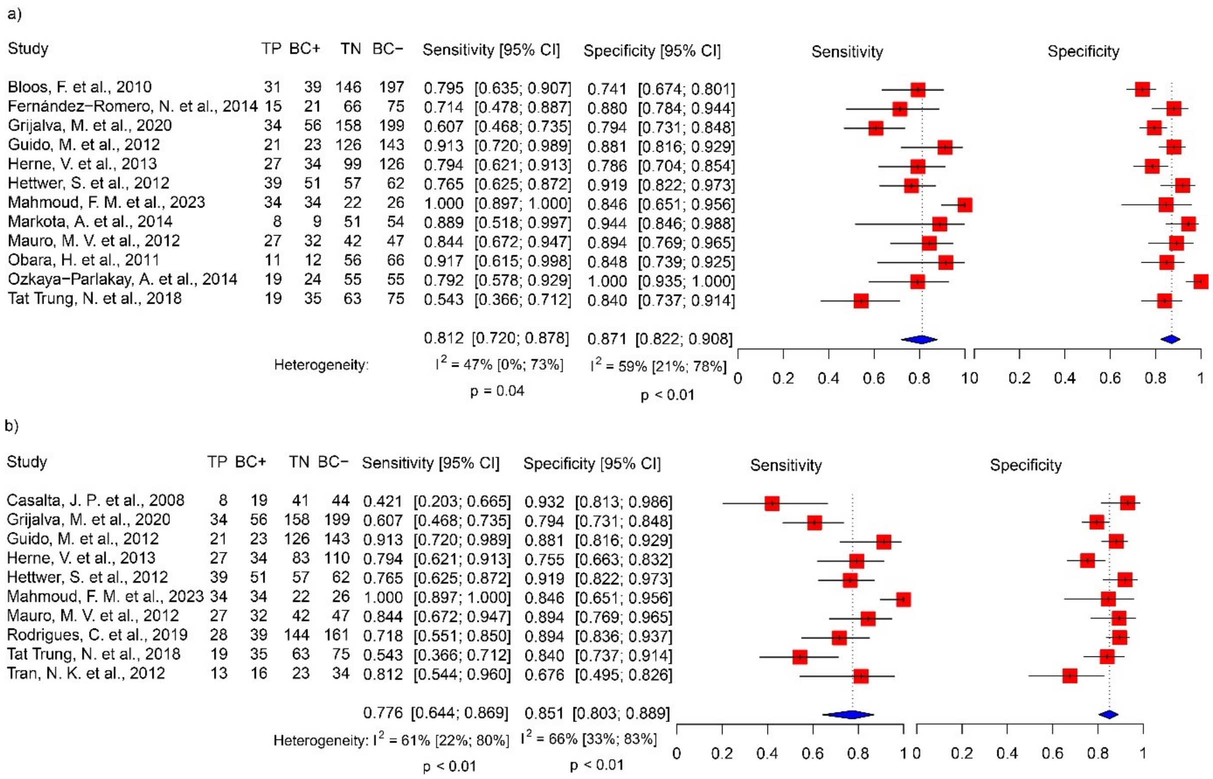

Abbreviations: TP=true positive. BC+=blood culture positive. TN=true negative. BC-=blood culture negative. CI=confidence interval.

Figure S7. Pooled specificity and sensitivity of rapid molecular assays when compared to blood culture. Population-based subgroup analyses are displayed in A-D plots. Unit of analysis: sample.
A) Immunosupressed patient population. B) Emergency department patient population. C) "Mixed" patient population. D) Intensive care patient population.

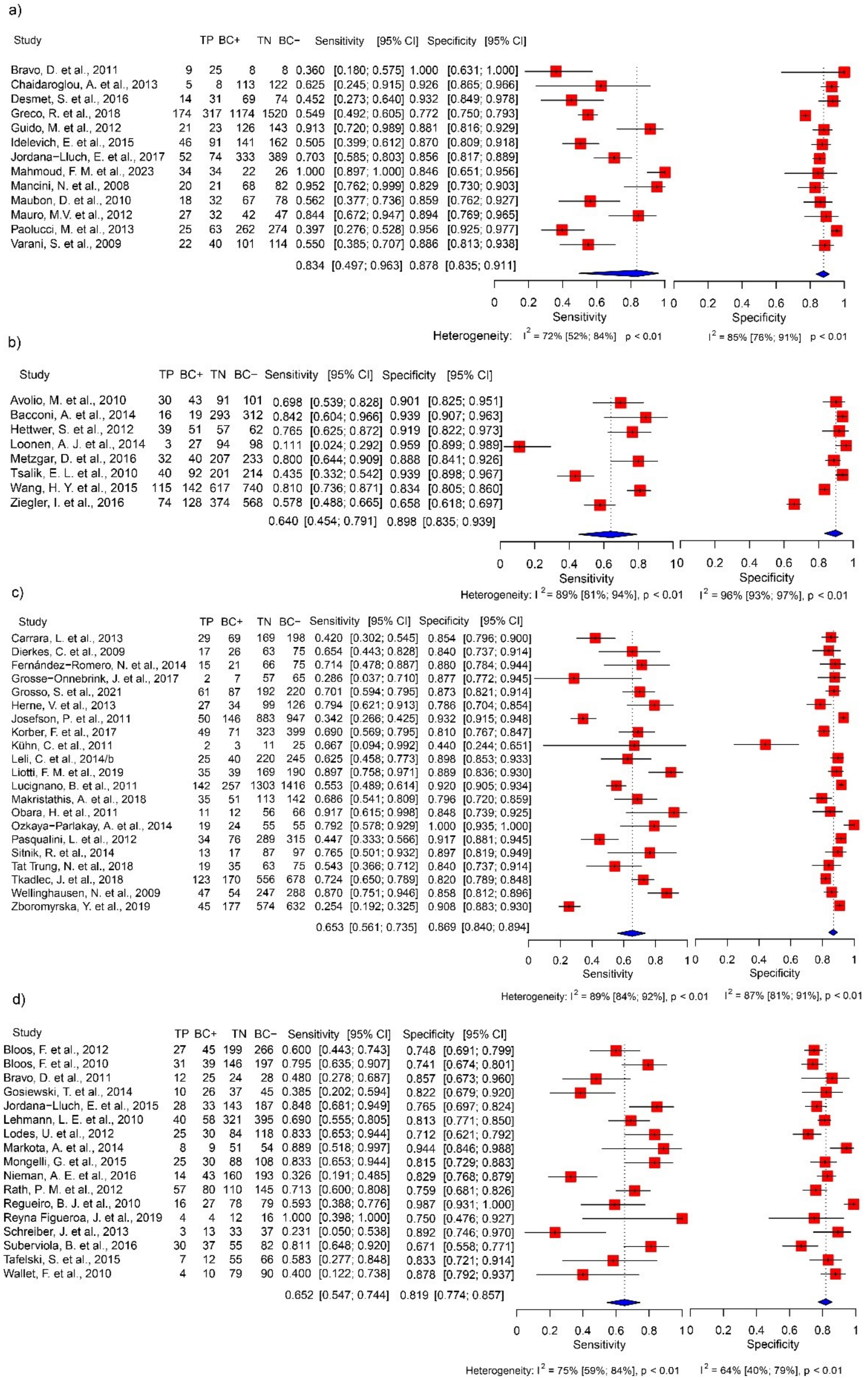

Abbreviations: TP=true positive. BC+=blood culture positive. TN=true negative. BC-=blood culture negative. CI=confidence interval.

Figure S8. Pooled specificity and sensitivity of rapid molecular assays when compared to blood culture. Population-based subgroup analyses are displayed in A-D plots. Unit of analysis: patient.

A) Immunosuppressed patient population. B) Emergency department patient population. C) “Mixed” patient population. D) Intensive care patient population.

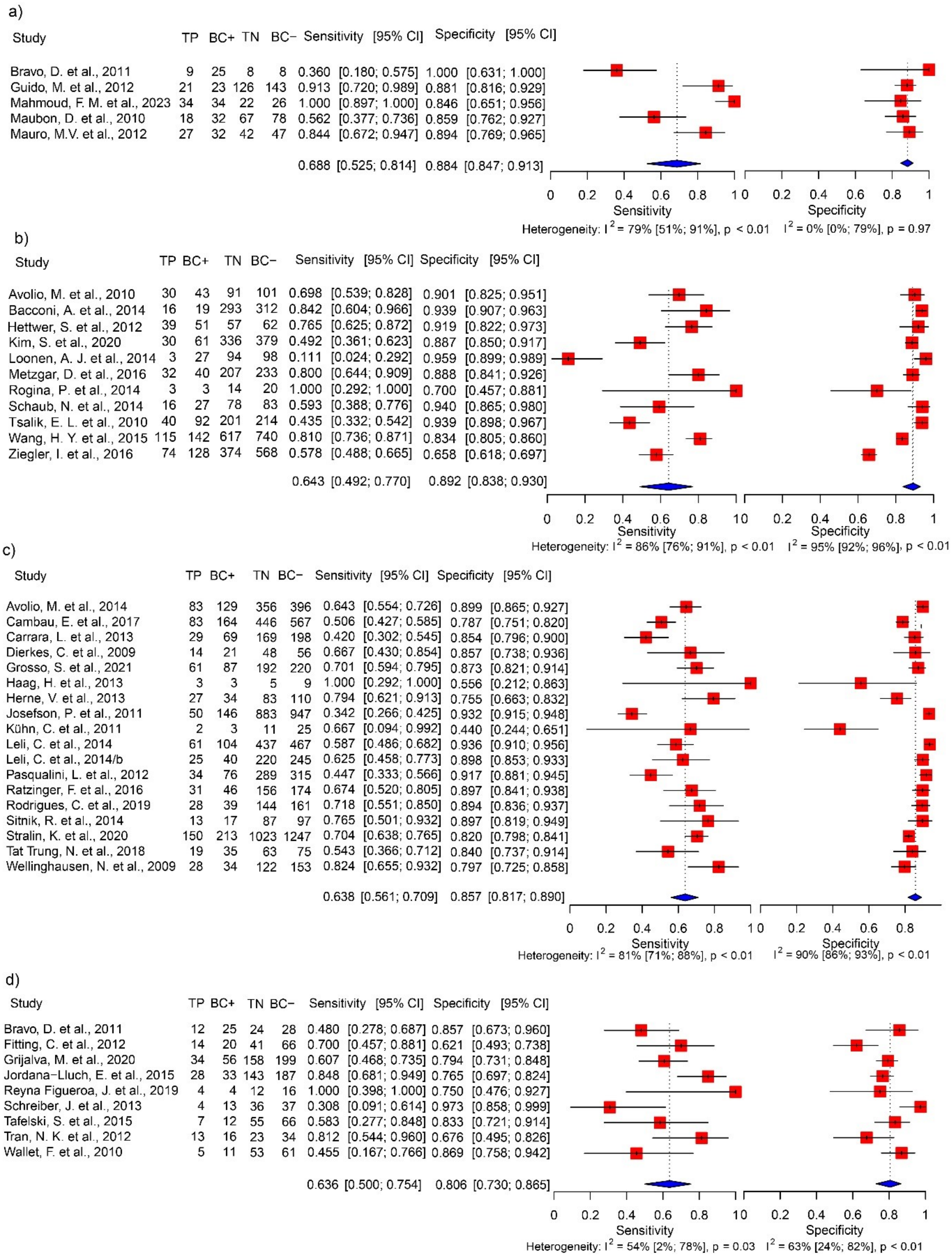

Abbreviations: TP=true positive. BC+=blood culture positive. TN=true negative. BC-=blood culture negative. CI=confidence interval.

Figure S9. Summary of evidence tables (GRADE approach)

Question: Should rapid molecular assays be used for the diagnosis of bloodstream infection in patients with suspected sepsis? Unit of analysis: sample.

Sensitivity	0.67 (95% CI: 0.61 to 0.72)
Specificity	0.86 (95% CI: 0.83 to 0.88)

Outcome	N of studies (N of samples)	Study design	Factors that may decrease certainty of evidence					Effect per 100 patients tested	Test accuracy CoE
			Risk of bias	Indirectness	Inconsistency	Imprecision	Publication bias	pre-test probability of 18.17%	
**True positives** (patients with bloodstream infection)	62 studies 3,409 samples	cross-sectional (cohort type accuracy study)	serious[a]	serious[b]	serious[c]	not serious	publication bias strongly suspected[d]	12 (11 to 13)	⊕○○○ Very low
**False negatives** (patients incorrectly classified as not having bloodstream infection)								6 (5 to 7)	
**True negatives** (patients without bloodstream infection)	62 studies 14,543 samples	cross-sectional (cohort type accuracy study)	serious[a]	serious[b]	serious[c]	not serious	publication bias strongly suspected[d]	70 (68 to 72)	⊕○○○ Very low
**False positives** (patients incorrectly classified as having bloodstream infection)								12 (10 to 14)	

Explanations

a. Downgraded because the gold standard hemoculture is not likely to correctly classify the target condition. In 12 of the 62 studies the index test results were interpreted without the knowledge of the results of the reference test, although it was not sufficient to downgrade the evidence. In 1 case-control type of study, we included data regarding the case-cohort, therefore we did not downgrade the quality of evidence.

b. Different rapid molecular assays (RMA) were compared to blood culture (BC), thus the results of different devices are pooled. Due to the different index tests, we downgraded the quality of evidence by one for indirectness.

c. The examined outcome showed a high between-study heterogeneity (sensitivity: I^2=85% [81%; 88%], specificity: I^2=91% [89%; 92%]).

d. Publication bias was examined with Deek's test (p= 0.0034).

Question: Should rapid molecular assays be used for the diagnosis of bloodstream infection in patients with suspected sepsis? Unit of analysis: patient.

Sensitivity	0.66 (95% CI: 0.59 to 0.72)
Specificity	0.86 (95% CI: 0.83 to 0.88)

Outcome	N of studies (N of patients)	Study design	Factors that may decrease certainty of evidence					Effect per 100 patients tested	Test accuracy CoE
			Risk of bias	Indirectness	Inconsistency	Imprecision	Publication bias	pre-test probability of 18.56%	
**True positives** (patients with bloodstream infection)	42 studies 2,203 patients	cross-sectional (cohort type accuracy study)	serious[a]	serious[b]	serious[c]	not serious	none	12 (11 to 13)	⊕○○○ Very low
**False negatives** (patients incorrectly classified as not having bloodstream infection)								7 (6 to 8)	
**True negatives** (patients without bloodstream infection)	42 studies 9,190 patients	cross-sectional (cohort type accuracy study)	serious[a]	serious[b]	serious[c]	not serious	none	70 (68 to 72)	⊕○○○ Very low
**False positives** (patients incorrectly classified as having bloodstream infection)								11 (9 to 13)	

Explanations

a. Downgraded because the gold standard hemoculture is not likely to correctly classify the target condition. In 7 of the 43 studies, the index test results were interpreted without the knowledge of the results of the reference test, though it was not sufficient to downgrade the evidence. In 1 case-control type of study we included data regarding the case-cohort, therefore we did not downgrade the quality of evidence.

b. Rapid molecular assays (RMA) were compared to hemoculture (BC), thus the results of different devices are pooled. Due to the different index tests, we downgraded the quality of evidence by one for indirectness.

c. The examined outcome showed a high between-study heterogeneity (sensitivity: I^2=78% [71%; 83%], specificity: I^2=90% [88%; 92%]).

Abbreviation: CoE=certainty of evidence. N=number. CI=confidence interval.

Figure S10. The Deek's funnel-plot asymmetry test. Filled dots represent individual studies, dashed line represents the regression line.
A) Articles where the unit of analysis was sample. B) Articles where the unit of analysis was patient.

a)

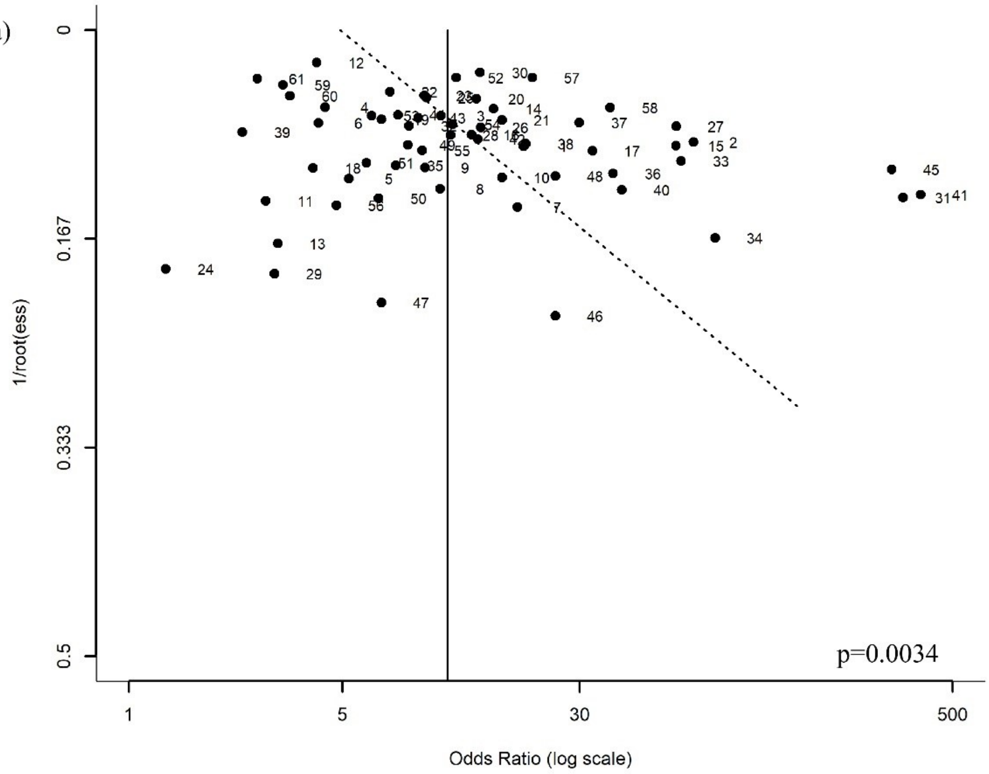

b)

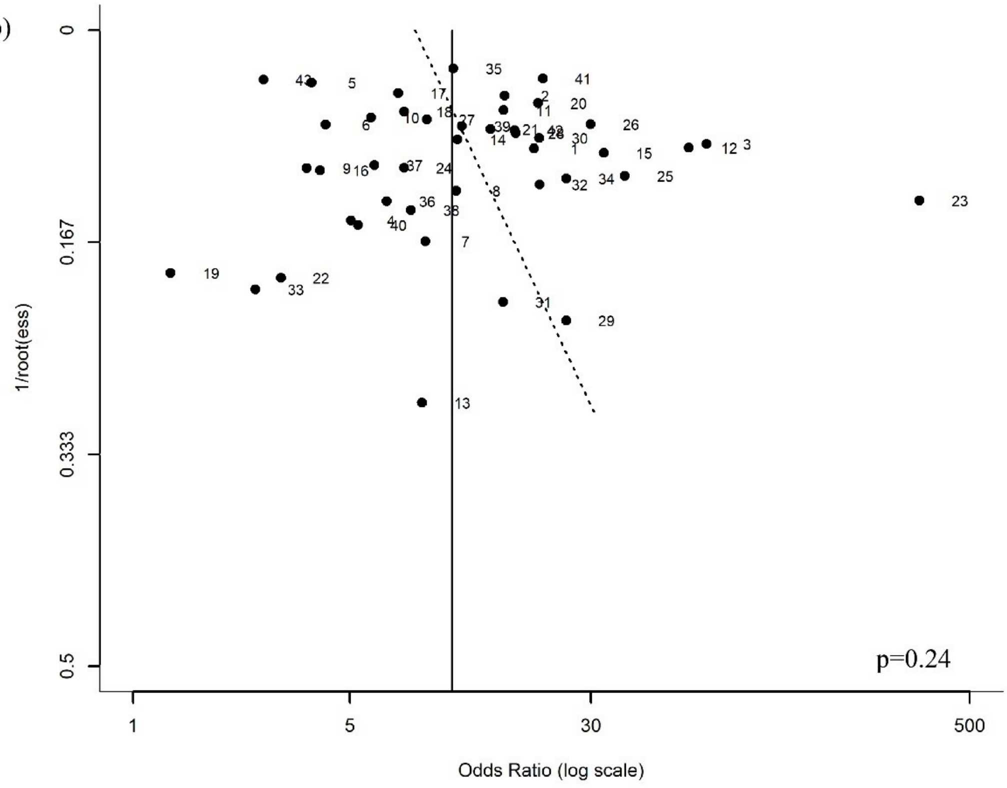

Figure S11. Supplementary analysis. Receiver operating curve (ROC) plots of the diagnostic accuracy of the LightCycler SeptiFast Test MGRADE®and blood culture with 95% confidence (ellipses with dashed lines) and 95% prediction regions (ellipses with continuous lines).

The perfection of the reference test is not assumed. Filled red and green circles represent summary diagnostic accuracy estimates. A) Analysis of articles where at least two blood culture sets were performed. B) Analysis of articles where less than two blood culture sets were performed or the number of sets was unavailable.

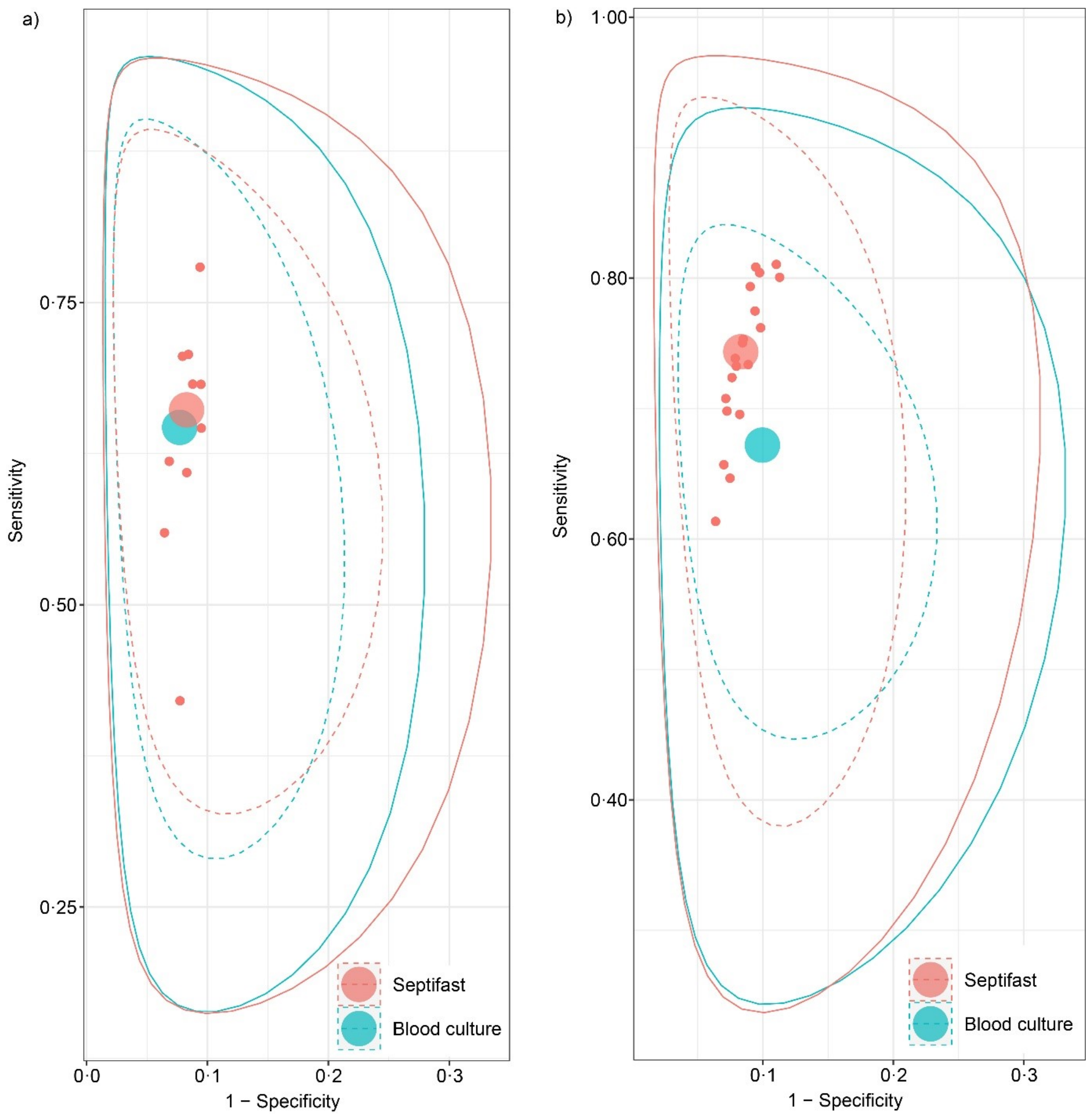

References

1 Stevenson M, Pandor A, Martyn-St James M, et al. Sepsis: the LightCycler SeptiFast Test MGRADE®, SepsiTest™ and IRIDICA BAC BSI assay for rapidly identifying bloodstream bacteria and fungi - a systematic review and economic evaluation. *Health Technol Assess* 2016; **20**(46): 1–246.

2 Avolio M, Diamante P, Zamparo S, et al. Molecular identification of bloodstream pathogens in patients presenting to the emergency department with suspected sepsis. *Shock* 2010; **34**(1): 27–30.

3 Avolio M, Diamante P, Modolo ML, De Rosa R, Stano P, Camporese A. Direct Molecular Detection of Pathogens in Blood as Specific Rule-In Diagnostic Biomarker in Patients With Presumed Sepsis: Our Experience on a Heterogeneous Cohort of Patients With Signs of Infective Systemic Inflammatory Response Syndrome. *Shock* 2014; **42**(2): 86–92.

4 Bacconi A, Richmond GS, Baroldi MA, et al. Improved sensitivity for molecular detection of bacterial and Candida infections in blood. *J Clin Microbiol* 2014; **52**(9): 3164–74.

5 Bloos F, Sachse S, Kortgen A, et al. Evaluation of a polymerase chain reaction assay for pathogen detection in septic patients under routine condition: an observational study. *PLoS One* 2012; **7**(9): Sep 27. DOI: 10.1371/journal.pone.0046003.

6 Bloos F, Hinder F, Becker K, et al. A multicenter trial to compare blood culture with polymerase chain reaction in severe human sepsis. *Intensive Care Med* 2010; **36**(2): 241–7.

7 Bravo D, Blanquer J, Tormo M, et al. Diagnostic accuracy and potential clinical value of the LightCycler SeptiFast assay in the management of bloodstream infections occurring in neutropenic and critically ill patients. *International journal of infectious diseases : IJID : official publication of the International Society for Infectious Diseases* 2011; **15**: e326–31.

8 Cambau E, Durand-Zaleski I, Bretagne S, et al. Performance and economic evaluation of the molecular detection of pathogens for patients with severe infections: the EVAMICA open-label, cluster-randomised, interventional crossover trial. *Intensive Care Med* 2017; **43**(11): 1613–25.

9 Carrara L, Navarro F, Turbau M, et al. Molecular diagnosis of bloodstream infections with a new dual-priming oligonucleotide-based multiplex PCR assay. *J Med Microbiol* 2013; **62**(Pt 11): 1673–9.

10 Casalta JP, Gouriet F, Roux V, Thuny F, Habib G, Raoult D. Evaluation of the LightCycler SeptiFast test in the rapid etiologic diagnostic of infectious endocarditis. *Eur J Clin Microbiol Infect Dis* 2009; **28**(6): 569–73.

11 Chaidaroglou A, Manoli E, Marathias E, et al. Use of a multiplex polymerase chain reaction system for enhanced bloodstream pathogen detection in thoracic transplantation. *J Heart Lung Transplant* 2013; **32**(7): 707–13.

12 Desmet S, Maertens J, Bueselinck K, Lagrou K. Broad-Range PCR Coupled with Electrospray Ionization Time of Flight Mass Spectrometry for Detection of Bacteremia and Fungemia in Patients with Neutropenic Fever. *J Clin Microbiol* 2016; **54**(10): 2513–20.

13 Dierkes C, Ehrenstein B, Siebig S, Linde HJ, Reischl U, Salzberger B. Clinical impact of a commercially available multiplex PCR system for rapid detection of pathogens in patients with presumed sepsis. *BMC Infect Dis* 2009; **9**: 126.

14 Fernández-Romero N, Quiles I, Jiménez C, et al. Use of multiplex PCR in diagnosis of bloodstream infections in kidney patients. *Diagn Microbiol Infect Dis* 2014; **80**(2): 93–6.

15 Fitting C, Parlato M, Adib-Conquy M, et al. DNAemia detection by multiplex PCR and biomarkers for infection in systemic inflammatory response syndrome patients. *PLoS One* 2012; **7**(6): Jun 15. DOI: 0.1371/journal.pone.0038916.

16 Gosiewski T, Flis A, Sroka A, et al. Comparison of nested, multiplex, qPCR; FISH; SeptiFast and blood culture methods in detection and identification of bacteria and fungi in blood of patients with sepsis. *BMC Microbiology* 2014; **14**(1): 313.

17 Greco R, Barbanti MC, Mancini N, et al. Adjuvant role of SeptiFast to improve the diagnosis of sepsis in a large cohort of hematological patients. *Bone Marrow Transplant* 2018; **53**(4): 410–6.

18 Grijalva M, De La Torre K, Sánchez N. The clinical impact of a multiplex real-time PCR system for microbiological diagnosis of sepsis: a mortality study. *New Microbiol* 2020; **43**(2): 64–9.

19 Grosse-Onnebrink J, Stehling F, Tschiedel E, et al. Bacteraemia and fungaemia in cystic fibrosis patients with febrile pulmonary exacerbation: a prospective observational study. *BMC Pulm Med* 2017; **17**(1): June 29. DOI: 10.1186/s12890-017-0440-4.

20 Grosso S, Pagani L, Tosoni N, et al. A new molecular method for rapid etiological diagnosis of sepsis with improved performance. *Future Microbiol* 2021; **16**: 741–51.

21 Guido M, Quattrocchi M, Zizza A, et al. Molecular approaches in the diagnosis of sepsis in neutropenic patients with haematological malignances. *J Prev Med Hyg* 2012; **53**(2): 104–8.

22 Haag H, Locher F, Nolte O. Molecular diagnosis of microbial aetiologies using SepsiTest™ in the daily routine of a diagnostic laboratory. *Diagn Microbiol Infect Dis* 2013; **76**(4): 413–8.
23 Herne V, Nelovkov A, Kütt M, Ivanova M. Diagnostic performance and therapeutic impact of LightCycler SeptiFast assay in patients with suspected sepsis. *European Journal of Microbiology and Immunology EuJMI* 2013; **3**(1): 68–76.
24 Hettwer S, Wilhelm J, Schürmann M, et al. Microbial diagnostics in patients with presumed severe infection in the emergency department. *Med Klin Intensivmed Notfmed* 2012; **107**(1): 53–62.
25 Idelevich EA, Silling G, Niederbracht Y, et al. Impact of multiplex PCR on antimicrobial treatment in febrile neutropenia: a randomized controlled study. *Med Microbiol Immunol* 2015; **204**(5): 585–92.
26 Jordana-Lluch E, Giménez M, Quesada MD, et al. Evaluation of the Broad-Range PCR/ESI-MS Technology in Blood Specimens for the Molecular Diagnosis of Bloodstream Infections. *PLoS One* 2015; **10**(10): Oct 16. DOI: 0.1371/journal.pone.0140865.
27 Jordana-Lluch E, Rivaya B, Marcó C, et al. Molecular diagnosis of bloodstream infections in onco-haematology patients with PCR/ESI-MS technology. *J Infect* 2017; **74**(2): 187–94.
28 Josefson P, Strålin K, Ohlin A, et al. Evaluation of a commercial multiplex PCR test (SeptiFast) in the etiological diagnosis of community-onset bloodstream infections. *Eur J Clin Microbiol Infect Dis* 2011; **30**(9): 1127–34.
29 Kim S, Kim J, Kim HY, Uh Y, Lee H. Efficient Early Diagnosis of Sepsis Using Whole-Blood PCR-Reverse Blot Hybridization Assay Depending on Serum Procalcitonin Levels. *Front Med (Lausanne)* 2020; **7**: Jul 31. DOI: 10.3389/fmed.2020.00390.
30 Korber F, Zeller I, Grünstäudl M, et al. SeptiFast versus blood culture in clinical routine - A report on 3 years experience. *Wien Klin Wochenschr* 2017; **129**(11-12): 427–34.
31 Kühn C, Disqué C, Mühl H, Orszag P, Stiesch M, Haverich A. Evaluation of commercial universal rRNA gene PCR plus sequencing tests for identification of bacteria and fungi associated with infectious endocarditis. *J Clin Microbiol* 2011; **49**(8): 2919–23.
32 Lehmann LE, Hunfeld KP, Steinbrucker M, et al. Improved detection of blood stream pathogens by real-time PCR in severe sepsis. *Intensive Care Med* 2010; **36**(1): 49–56.
33 Leli C, Cardaccia A, Ferranti M, et al. Procalcitonin better than C-reactive protein, erythrocyte sedimentation rate, and white blood cell count in predicting DNAemia in patients with sepsis. *Scand J Infect Dis* 2014; **46**(11): 745–52.
34 Leli C, Cardaccia A, D'Alò F, Ferri C, Bistoni F, Mencacci A. A prediction model for real-time PCR results in blood samples from febrile patients with suspected sepsis. *J Med Microbiol* 2014; **63**(Pt 5): 649–58.
35 Liotti FM, Posteraro B, Mannu F, et al. Development of a Multiplex PCR Platform for the Rapid Detection of Bacteria, Antibiotic Resistance, and Candida in Human Blood Samples. *Front Cell Infect Microbiol* 2019; **9**: 389.
36 Lodes U, Bohmeier B, Lippert H, König B, Meyer F. PCR-based rapid sepsis diagnosis effectively guides clinical treatment in patients with new onset of SIRS. *Langenbecks Arch Surg* 2012; **397**(3): 447–55.
37 Loonen AJ, de Jager CP, Tosserams J, et al. Biomarkers and molecular analysis to improve bloodstream infection diagnostics in an emergency care unit. *PLoS One* 2014; **9**(1): Jan 27. DOI: 10.1371/journal.pone.0087315.
38 Lucignano B, Ranno S, Liesenfeld O, et al. Multiplex PCR allows rapid and accurate diagnosis of bloodstream infections in newborns and children with suspected sepsis. *J Clin Microbiol* 2011; **49**(6): 2252–8.
39 Mahmoud FM, Khedr RA, Ebeid E, El-Mahallawy HA, Hassan SS. Impact of Rapid Molecular Diagnostic Technique on Time to Optimal Antimicrobial Therapy and Hospital Outcomes in Pediatric Cancer Patients with Sepsis. *Asian Pac J Cancer Prev* 2023; **24**(7): 2465–-71.
40 Makristathis A, Harrison N, Ratzinger F, et al. Substantial diagnostic impact of blood culture independent molecular methods in bloodstream infections: Superior performance of PCR/ESI-MS. *Scientific Reports* 2018; **8**(1): October 30. DOI: 10.1038/s41598-018-34298-7.
41 Mancini N, Clerici D, Diotti R, et al. Molecular diagnosis of sepsis in neutropenic patients with haematological malignancies. *J Med Microbiol* 2008; **57**(Pt 5): 601–4.
42 Markota A, Seme K, Golle A, Poljak M, Sinkovič A. SeptiFast real-time PCR for detection of bloodborne pathogens in patients with severe sepsis or septic shock. *Coll Antropol* 2014; **38**(3): 829–33.
43 Maubon D, Hamidfar-Roy R, Courby S, et al. Therapeutic impact and diagnostic performance of multiplex PCR in patients with malignancies and suspected sepsis. *J Infect* 2010; **61**(4): 335–42.
44 Mauro MV, Cavalcanti P, Perugini D, Noto A, Sperlì D, Giraldi C. Diagnostic utility of LightCycler SeptiFast and procalcitonin assays in the diagnosis of bloodstream infection in immunocompromised patients. *Diagn Microbiol Infect Dis* 2012; **73**(4): 308–11.
45 Metzgar D, Frinder MW, Rothman RE, et al. The IRIDICA BAC BSI Assay: Rapid, Sensitive and Culture-Independent Identification of Bacteria and Candida in Blood. *PLoS One* 2016; **11**(7): Jul 6. DOI: 10.1371/journal.pone.0158186.

46 Mongelli G, Romeo MA, Denaro C, Gennaro M, Fraggetta F, Stefani S. Added value of multi-pathogen probe-based real-time PCR SeptiFast in the rapid diagnosis of bloodstream infections in patients with bacteraemia. *J Med Microbiol* 2015; **64**(7): 670–5.
47 Nieman AE, Savelkoul PHM, Beishuizen A, et al. A prospective multicenter evaluation of direct molecular detection of blood stream infection from a clinical perspective. *BMC Infect Dis* 2016; **16**: June 30. DOI: 10.1186/s12879-016-1646-4.
48 Obara H, Tanabe M, Kitajima M, et al. The role of a real-time PCR technology for rapid detection and identification of bacterial and fungal pathogens in whole-blood samples. *Journal of Infection and Chemotherapy* 2011; **17**(3): 327–33.
49 Ortiz Ibarra J, Trevino Valdez P, Valenzuela Mendez E, et al. Evaluation of the Light-Cycler® SeptiFast Test in Newborns With Suspicion of Nosocomial Sepsis. *Iran J Pediatr* 2015; **25**(1): Jan 17. DOI: 0.5812/ijp.253.
50 Ozkaya-Parlakay A, Cengiz AB, Ceyhan M, et al. Evaluation of multiplex real time polymerase chain reaction and procalcitonin in the diagnosis of sepsis. *Clin Lab* 2014; **60**(7): 1075–81.
51 Paolucci M, Stanzani M, Melchionda F, et al. Routine use of a real-time polymerase chain reaction method for detection of bloodstream infections in neutropaenic patients. *Diagn Microbiol Infect Dis* 2013; **75**(2): 130–4.
52 Pasqualini L, Mencacci A, Leli C, et al. Diagnostic performance of a multiple real-time PCR assay in patients with suspected sepsis hospitalized in an internal medicine ward. *J Clin Microbiol* 2012; **50**(4): 1285–8.
53 Rath PM, Saner F, Paul A, et al. Multiplex PCR for rapid and improved diagnosis of bloodstream infections in liver transplant recipients. *J Clin Microbiol* 2012; **50**(6): 2069–71.
54 Ratzinger F, Tsirkinidou I, Haslacher H, et al. Evaluation of the Septifast MGrade Test on Standard Care Wards--A Cohort Study. *PLoS One* 2016; **11**(3): Mar 17. DOI: 0.1371/journal.pone.0151108.
55 Regueiro BJ, Varela-Ledo E, Martinez-Lamas L, et al. Automated extraction improves multiplex molecular detection of infection in septic patients. *PLoS One* 2010; **5**(10): Oct 13. DOI: 0.1371/journal.pone.0013387.
56 Reyna Figueroa J, Rodríguez-Sánchez M, Matsumoto P, Ortiz- Ibarra F, Limón-Rojas A. Decrease in the Hospital Stay of Neonates with Suspected Nosocomial Sepsis with the Use of a Molecular Biology Technique. *Journal of Biosciences and Medicines* 2019; **07**: 44–51.
57 Rodrigues C, Siciliano RF, Filho HC, et al. The effect of a rapid molecular blood test on the use of antibiotics for nosocomial sepsis: a randomized clinical trial. *J Intensive Care* 2019; **7**: July 22. DOI: 10.1186/s40560-019-0391-3.
58 Rogina P, Skvarc M, Stubljar D, Kofol R, Kaasch A. Diagnostic utility of broad range bacterial 16S rRNA gene PCR with degradation of human and free bacterial DNA in bloodstream infection is more sensitive than an in-house developed PCR without degradation of human and free bacterial DNA. *Mediators Inflamm* 2014; **2014**: July 9. DOI: 10.1155/2014/108592.
59 Schaub N, Boldanova T, Noveanu M, et al. Incremental value of multiplex real-time PCR for the early diagnosis of sepsis in the emergency department. *Swiss Med Wkly* 2014; **144**: February 4. DOI: 10.4414/smw.2014.13911.
60 Schreiber J, Nierhaus A, Braune SA, de Heer G, Kluge S. Comparison of three different commercial PCR assays for the detection of pathogens in critically ill sepsis patients. *Med Klin Intensivmed Notfmed* 2013; **108**(4): 311–8.
61 Sitnik R, Marra AR, Petroni RC, et al. SeptiFast for diagnosis of sepsis in severely ill patients from a Brazilian hospital. *Einstein (Sao Paulo)* 2014; **12**(2): 191–6.
62 Strålin K, Rothman RE, Özenci V, et al. Performance of PCR/Electrospray Ionization-Mass Spectrometry on Whole Blood for Detection of Bloodstream Microorganisms in Patients with Suspected Sepsis. *J Clin Microbiol* 2020; **58**(9): August 24. DOI: 10.1128/jcm.01860-19.
63 Suberviola B, Márquez-López A, Castellanos-Ortega A, Fernández-Mazarrasa C, Santibáñez M, Martínez LM. Microbiological Diagnosis of Sepsis: Polymerase Chain Reaction System Versus Blood Cultures. *Am J Crit Care* 2016; **25**(1): 68–75.
64 Tafelski S, Nachtigall I, Adam T, et al. Randomized controlled clinical trial evaluating multiplex polymerase chain reaction for pathogen identification and therapy adaptation in critical care patients with pulmonary or abdominal sepsis. *J Int Med Res* 2015; **43**(3): 364–77.
65 Tat Trung N, Van Tong H, Lien TT, et al. Clinical utility of an optimised multiplex real-time PCR assay for the identification of pathogens causing sepsis in Vietnamese patients. *Int J Infect Dis* 2018; **67**: 122–8.
66 Tkadlec J, Peckova M, Sramkova L, et al. The use of broad-range bacterial PCR in the diagnosis of infectious diseases: a prospective cohort study. *Clin Microbiol Infect* 2019; **25**(6): 747–52.
67 Tran NK, Wisner DH, Albertson TE, et al. Multiplex polymerase chain reaction pathogen detection in patients with suspected septicemia after trauma, emergency, and burn surgery. *Surgery* 2012; **151**(3): 456–63.

68 Tröger B, Härtel C, Buer J, et al. Clinical Relevance of Pathogens Detected by Multiplex PCR in Blood of Very-Low-Birth Weight Infants with Suspected Sepsis - Multicentre Study of the German Neonatal Network. *PLoS One* 2016; **11**(7): Jul 29. DOI: 10.1371/journal.pone.0159821.
69 Tsalik EL, Jones D, Nicholson B, et al. Multiplex PCR to diagnose bloodstream infections in patients admitted from the emergency department with sepsis. *J Clin Microbiol* 2010; **48**(1): 26–33.
70 Varani S, Stanzani M, Paolucci M, et al. Diagnosis of bloodstream infections in immunocompromised patients by real-time PCR. *J Infect* 2009; **58**(5): 346–51.
71 Wallet F, Nseir S, Baumann L, et al. Preliminary clinical study using a multiplex real-time PCR test for the detection of bacterial and fungal DNA directly in blood. *Clin Microbiol Infect* 2010; **16**(6): 774–9.
72 Wang HY, Kim J, Kim S, et al. Performance of PCR-REBA assay for screening and identifying pathogens directly in whole blood of patients with suspected sepsis. *Journal of Applied Microbiology* 2015; **119**(5): 1433–42.
73 Wellinghausen N, Kochem AJ, Disqué C, et al. Diagnosis of bacteremia in whole-blood samples by use of a commercial universal 16S rRNA gene-based PCR and sequence analysis. *J Clin Microbiol* 2009; **47**(9): 2759–65.
74 Westh H, Lisby G, Breysse F, et al. Multiplex real-time PCR and blood culture for identification of bloodstream pathogens in patients with suspected sepsis. *Clin Microbiol Infect* 2009; **15**(6): 544–51.
75 Zboromyrska Y, Cillóniz C, Cobos-Trigueros N, et al. Evaluation of the Magicplex™ Sepsis Real-Time Test for the Rapid Diagnosis of Bloodstream Infections in Adults. *Front Cell Infect Microbiol* 2019; **9**: March 12. 0.3389/fcimb.2019.00056.
76 Ziegler I, Fagerström A, Strålin K, Mölling P. Evaluation of a Commercial Multiplex PCR Assay for Detection of Pathogen DNA in Blood from Patients with Suspected Sepsis. *PLoS One* 2016; **11**(12): Dec 20. DOI: 10.1371/journal.pone.0167883.
77 Burdino E, Ruggiero T, Allice T, et al. Combination of conventional blood cultures and the SeptiFast molecular test in patients with suspected sepsis for the identification of bloodstream pathogens. *Diagn Microbiol Infect Dis* 2014; **79**(3): 287–92.
78 Camp I, Manhart G, Schabereiter-Gurtner C, Spettel K, Selitsch B, Willinger B. Clinical evaluation of an in-house panfungal real-time PCR assay for the detection of fungal pathogens. *Infection* 2020; **48**(3): 345–55.
79 Camp I, Füszl A, Selitsch B, et al. Is the T2MR Candida Panel a suitable alternative to the SeptiFast for the rapid diagnosis of candidemia in routine clinical practice? *Clin Microbiol Infect* 2024; **30**(6): 816-21.
80 Cortegiani A, Russotto V, Montalto F, et al. Procalcitonin as a marker of Candida species detection by blood culture and polymerase chain reaction in septic patients. *BMC Anesthesiology* 2014; **14**(1): 9.
81 Delcò C, Karam O, Pfister R, et al. Rapid detection and ruling out of neonatal sepsis by PCR coupled with Electrospray Ionization Mass Spectrometry (PCR/ESI-MS). *Early Hum Dev* 2017; **108**: 17–22.
82 Dinç F, Akalin H, Özakin C, et al. Comparison of blood culture and multiplex real-time PCR for the diagnosis of nosocomial sepsis. *Minerva Anestesiol* 2016; **82**(3): 301–9.
83 Elges S, Arnold R, Liesenfeld O, et al. Prospective evaluation of the SeptiFAST multiplex real-time PCR assay for surveillance and diagnosis of infections in haematological patients after allogeneic stem cell transplantation compared to routine microbiological assays and an in-house real-time PCR method. *Mycoses* 2017; **60**(12): 781–8.
84 Gies F, Tschiedel E, Felderhoff-Müser U, Rath P-M, Steinmann J, Dohna-Schwake C. Prospective evaluation of SeptiFast Multiplex PCR in children with systemic inflammatory response syndrome under antibiotic treatment. *BMC Infectious Diseases* 2016; **16**(1): 378.
85 Huber S, Weinberger J, Pilecky M, et al. A high leukocyte count and administration of hydrocortisone hamper PCR-based diagnostics for bloodstream infections. *Eur J Clin Microbiol Infect Dis* 2021; **40**(7): 1441–9.
86 Kasper DC, Altiok I, Mechtler TP, et al. Molecular detection of late-onset neonatal sepsis in premature infants using small blood volumes: proof-of-concept. *Neonatology* 2013; **103**(4): 268–73.
87 Lamoth F, Jaton K, Prod'hom G, et al. Multiplex blood PCR in combination with blood cultures for improvement of microbiological documentation of infection in febrile neutropenia. *J Clin Microbiol* 2010; **48**(10): 3510–6.
88 Lehmann LE, Alvarez J, Hunfeld KP, et al. Potential clinical utility of polymerase chain reaction in microbiological testing for sepsis. *Crit Care Med* 2009; **37**(12): 3085–90.
89 Ljungström L, Enroth H, Claesson BE, et al. Clinical evaluation of commercial nucleic acid amplification tests in patients with suspected sepsis. *BMC Infect Dis* 2015; **15**: April 28. DOI: 10.1186/s12879-015-0938-4.
90 Louie RF, Tang Z, Albertson TE, Cohen S, Tran NK, Kost GJ. Multiplex polymerase chain reaction detection enhancement of bacteremia and fungemia. *Crit Care Med* 2008; **36**(5): 1487–92.
91 Matsushima A, Tasaki O, Shimazu T. Potential Clinical Usefulness of the Polymerase Chain Reaction Test to Detect Pathogens Causing Sepsis. *Journal of Medical Microbiology & Diagnosis* 2012; **01**: March 9. DOI: 10.4172/2161-0703.1000106.

92 Mencacci A, Leli C, Cardaccia A, et al. Procalcitonin predicts real-time PCR results in blood samples from patients with suspected sepsis. *PLoS One* 2012; **7**(12): Dec 27. DOI: 10.1371/journal.pone.0053279.
93 Mihajlovic D, Brkic S, Uvelin A, Draskovic B, Vrsajkov V. Use of presepsin and procalcitonin for prediction of SeptiFast results in critically ill patients. *J Crit Care* 2017; **40**: 197–201.
94 Phung TTB, Suzuki T, Phan PH, et al. Pathogen screening and prognostic factors in children with severe ARDS of pulmonary origin. *Pediatr Pulmonol* 2017; **52**(11): 1469–77.
95 Pilarczyk K, Rath PM, Steinmann J, et al. Multiplex polymerase chain reaction to diagnose bloodstream infections in patients after cardiothoracic surgery. *BMC Anesthesiol* 2019; **19**(1): April 23. DOI: 10.1186/s12871-019-0727-5
96 Simms LA, Davies C, Jayasundara N, et al. Performance evaluation of InfectID-BSI: A rapid quantitative PCR assay for detecting sepsis-associated organisms directly from whole blood. *J Microbiol Methods* 2023; **211**: 106783.
97 Stein A, Soukup D, Rath PM, Felderhoff-Müser U. Diagnostic Accuracy of Multiplex Polymerase Chain Reaction in Early Onset Neonatal Sepsis. *Children (Basel)* 2023; **10**(11).
98 Straub J, Paula H, Mayr M, et al. Diagnostic accuracy of the ROCHE Septifast PCR system for the rapid detection of blood pathogens in neonatal sepsis-A prospective clinical trial. *PLoS One* 2017; **12**(11): Nov 8. DOI: 10.1371/journal.pone.0187688.
99 Tassinari M, Zannoli S, Farabegoli P, et al. Rapid diagnosis of bloodstream infections in the critically ill: Evaluation of the broad-range PCR/ESI-MS technology. *PLoS One* 2018; **13**(5): May 15. DOI: 0.1371/journal.pone.0197436.
100 Tschiedel E, Steinmann J, Buer J, et al. Results and relevance of molecular detection of pathogens by SeptiFast--a retrospective analysis in 75 critically ill children. *Klin Padiatr* 2012; **224**(1): 12–6.
101 Vincent JL, Brealey D, Libert N, et al. Rapid Diagnosis of Infection in the Critically Ill, a Multicenter Study of Molecular Detection in Bloodstream Infections, Pneumonia, and Sterile Site Infections. *Crit Care Med* 2015; **43**(11): 2283–91.
102 von Lilienfeld-Toal M, Lehmann LE, Raadts AD, et al. Utility of a commercially available multiplex real-time PCR assay to detect bacterial and fungal pathogens in febrile neutropenia. *J Clin Microbiol* 2009; **47**(8): 2405–10.
103 Warhurst G, Maddi S, Dunn G, et al. Diagnostic accuracy of SeptiFast multi-pathogen real-time PCR in the setting of suspected healthcare-associated bloodstream infection. *Intensive Care Med* 2015; **41**(1): 86–93.
104 Wu Z, Yao Y, Li X, et al. Sensitive and rapid identification of pathogens by droplet digital PCR in a cohort of septic patients: a prospective diagnostic study. *Infect Dis (Lond)* 2024; **56**(10): 830–41.
105 Yanagihara K, Kitagawa Y, Tomonaga M, et al. Evaluation of pathogen detection from clinical samples by real-time polymerase chain reaction using a sepsis pathogen DNA detection kit. *Crit Care* 2010; **14**(4): August 24. DOI: 10.1186/cc9234.

Supplementary Data

Data S1. Data extracted and analyzed from studies included in the quantitative synthesis

**Author**	**year**	**index test**	**referenc e test**	**all samples**	**TP (samples)**	**TN (samples)**	**FP (samples)**	**FN (samples)**	**all patients**	**TP (patients)**	**TN (patients)**	**FP (patients)**	**FN (patients)**	**patient population**	**sponsor ship status**	**at least 2 blood culture sets***	**age category**	**Properly reported contamin ant results**
Avolio et al	2010	LightCycler SeptiFast Test MGRADE	Bact/Aler t 3D	144	30	91	10	13	144	30	91	10	13	emergency department	unclear	unclear	adults	yes
Avolio et al	2014	LightCycler SeptiFast Test MGRADE	BacT/Ale rt 3D	NA	NA	NA	NA	NA	525	83	356	40	46	mixed population	unclear	unclear	adults	yes
Bacconi et al	2014	IRIDICA BAC BSI	BACTEC FX	331	16	293	19	3	331	16	293	19	3	emergency department	yes	yes	adults	yes
Bloos et al	2012	VYOO	NA	311	27	199	67	18	NA	NA	NA	NA	NA	intensive care	yes	unclear	adults	yes
Bloos et al	2010	LightCycler SeptiFast Test MGRADE	NA	236	31	146	51	8	NA	NA	NA	NA	NA	intensive care	yes	unclear	adults	unclear
Bravo et al	2011	LightCycler SeptiFast Test MGRADE	Bactec 9420	53	12	24	4	13	53	12	24	4	13	intensive care	no	yes	adults	yes
Bravo et al	2011	LightCycler SeptiFast Test MGRADE	Bactec 9420	33	9	8	0	16	33	9	8	0	16	immunosup pression	no	yes	adults	yes
Cambau et al	2017	LightCycler SeptiFast Test MGRADE	NA	NA	NA	NA	NA	NA	731	83	446	121	81	mixed population	no	yes	adults	yes

Carrara et al	2013	MagicPlex Sepsis Test	BacT/Alert	267	29	169	29	40	267	29	169	29	40	mixed population	no	unclear	adults	yes
Casalta et al	2009	LightCycler SeptiFast Test MGRADE	BACTEC	NA	NA	NA	NA	NA	63	8	41	3	11	NA	yes	unclear	NA	NA
Chaidaroglou et al	2013	LightCycler SeptiFast Test MGRADE	Bactec 9120	130	5	113	9	3	NA	NA	NA	NA	NA	immunosuppression	no	unclear	adults	yes
Desmet et al	2016	IRIDICA BAC BSI	BacT/Alert	105	14	69	5	17	NA	NA	NA	NA	NA	immunosuppression	yes	yes	adults	yes
Dierkes et al	2009	LightCycler SeptiFast Test MGRADE	Bactec 9240	101	17	63	12	9	77	14	48	8	7	mixed population	yes	unclear	adults	yes
Fernández-Romero et al	2014	LightCycler SeptiFast Test MGRADE	Bact/Alert	96	15	66	9	6	NA	NA	NA	NA	NA	mixed population	yes	yes	adults	NA
Fitting et al	2012	VYOO	NA	NA	NA	NA	NA	NA	86	14	41	25	6	intensive care	yes	unclear	adults	unclear
Gosiewski et al	2014	LightCycler SeptiFast Test MGRADE	Bact/Alert 3D	71	10	37	8	16	NA	NA	NA	NA	NA	intensive care	no	unclear	adults	yes
Greco et al	2018	LightCycler SeptiFast Test MGRADE	Bact/Alert 3D	1837	174	1174	346	143	NA	NA	NA	NA	NA	immunosuppression	no	yes	NA	yes
Grijalva et al	2020	LightCycler SeptiFast Test MGRADE	NA	255	34	158	41	22	255	34	158	41	22	intensive care	no	unclear	NA	NA
Grosse-Onnebrink et al	2017	LightCycler SeptiFast Test MGRADE	BACTEC 9240	72	2	57	8	5	NA	NA	NA	NA	NA	mixed population	no	unclear	NA	yes
Grosso et al	2021	MicrobScan	BacT/Alert	307	70.7*	87.3†	68.5‡	88.1§	307	70.7*	87.3†	68.5‡	88.1§	mixed population	yes	yes	NA	unclear

Grosso et al	2021	MicrobScan-Kairos24/7	BacT/Alert	441	76.7*	95.1†	88.8‡	89§	441	76.7*	95.1†	88.8‡	89§	mixed population	yes	yes	NA	unclear
Guido et al	2012	LightCycler SeptiFast Test MGRADE	BacT/Alert 3D	166	21	126	17	2	166	21	126	17	2	immunosuppression	unclear	no	adults	unclear
Haag et al	2013	SepsiTest	BacT/ALERT	NA	NA	NA	NA	NA	12	3	5	4	0	mixed population	no	unclear	adults	yes
Herne et al	2013	LightCycler SeptiFast Test MGRADE	BacT/ALERT 3D	160	27	99	27	7	144	27	83	27	7	mixed population	no	yes	adults	unclear
Hettwer et al	2012	LightCycler SeptiFast Test MGRADE	BacT/Alert 3D	113	39	57	5	12	113	39	57	5	12	emergency department	yes	unclear	adults	unclear
Oritz Ibarra et al	2015	LightCycler SeptiFast Test MGRADE	BacT/ALERT	86	9	48	25	4	86	9	48	25	4	NA	no	unclear	newborn-infant	yes
Idelevich et al	2015	LightCycler SeptiFast Test MGRADE	BACTEC 9240	253	46	141	21	45	NA	NA	NA	NA	NA	immunosuppression	yes	no	NA	yes
Jordana-Lluch et al	2015	IRIDICA BAC BSI	Bactec 9240	220	28	143	44	5	NA	NA	NA	NA	NA	intensive care	yes	yes	NA	yes
Jordana-Lluch et al	2017	IRIDICA BAC BSI	Bactec 9420	463	52	333	56	22	NA	NA	NA	NA	NA	immunosuppression	yes	yes	NA	yes
Josefson et al	2011	LightCycler SeptiFast Test MGRADE	Bactec 9240	1093	50	883	64	96	1093	50	883	64	96	mixed population	yes	yes	NA	yes
Kim et al	2020	REBA Sepsis-ID	BacT/Alert 3D	NA	NA	NA	NA	NA	440	30	336	43	31	emergency department	no	yes	adults	yes
Korber et al	2017	LightCycler SeptiFast Test MGRADE	BacT/ALERT 3D	470	49	323	76	22	NA	NA	NA	NA	NA	mixed population	yes	no	NA	yes

Kühn et al	2011	SepsiTest	NA	28	2	11	14	1	28	2	11	14	1	mixed population	unclear	unclear	adults	yes
Lehmann et al	2010	LightCycler SeptiFast Test MGRADE	BACTEC 9240	453	40	321	74	18	NA	NA	NA	NA	NA	intensive care	yes	unclear	adults	yes
Leli et al/a	2014	LightCycler SeptiFast Test MGRADE	BACTEC FX	NA	NA	NA	NA	NA	571	61	437	30	43	mixed population	unclear	yes	NA	yes
Leli et al/b	2014	LightCycler SeptiFast Test MGRADE	Bactec FX	285	25	220	25	15	285	25	220	25	15	mixed population	unclear	yes	NA	yes
Liotti et al	2019	MicrobScan	BacT/Alert	229	35	169	21	4	0	NA	NA	25	NA	mixed population	yes	unclear	NA	yes
Lodes et al	2012	LightCycler SeptiFast Test MGRADE	BACTEC 9240	148	25	84	34	5	NA	NA	NA	NA	NA	intensive care	no	unclear	adults	yes
Loonen et al	2014	SepsiTest	BacT/ALERT	125	3	94	4	24	125	3	94	4	24	emergency department	yes	yes	adults	yes
Loonen et al	2014	MagicPlex Sepsis Test	BacT/ALERT	125	12	75	23	15	125	12	75	23	15	emergency department	yes	yes	adults	yes
Lucignano et al	2011	LightCycler SeptiFast Test MGRADE	BACTEC 9240/9120	1673	142	1303	113	115	803	NA	NA	NA	NA	mixed population	no	unclear	pediatric	yes
Mahmoud et al	2023	LightCycler SeptiFast Test MGRADE	Bactec 9120 BacT/Alert 3D	60	34	22	4	0	60	34	22	4	0	immunosuppression	no	unclear	pediatric	NA
Makristathis et al	2018	IRIDICA BAC BSI	BacT/Alert 3D	193	35	113	29	16	NA	NA	NA	NA	NA	mixed population	yes	yes	adults	yes
Makristathis et al	2018	LightCycler SeptiFast Test MGRADE	BacT/Alert 3D	193	31	131	11	20	NA	NA	NA	NA	NA	mixed population	yes	yes	adults	yes
Mancini et al	2008	LightCycler SeptiFast	BacT/Alert 3D	103	20	68	14	1	NA	NA	NA	NA	NA	immunosuppression	yes	unclear	adults	yes

		Test MGRADE																
Markota et al	2014	LightCycler SeptiFast Test MGRADE	BacT/Alert 3D	63	8	51	3	1	NA	NA	NA	NA	NA	intensive care	unclear	yes	adults	NA
Maubon et al	2010	LightCycler SeptiFast Test MGRADE	NA	110	18	67	11	14	110	18	67	11	14	immunosuppression	yes	yes	NA	yes
Mauro et al	2012	LightCycler SeptiFast Test MGRADE	Bactec 9240	79	27	42	5	5	79	27	42	5	5	immunosuppression	unclear	yes	NA	unclear
Metzgar et al	2016	IRIDICA BAC BSI	BACTEC FX 200	273	32	207	26	8	273	32	207	26	8	emergency department	unclear	yes	NA	yes
Mongelli et al	2015	LightCycler SeptiFast Test MGRADE	BacT/Alert 3D	138	25	88	20	5	NA	NA	NA	NA	NA	intensive care	unclear	yes	adults	yes
Nieman et al	2016	SepsiTest	BacT/Alert 3D	236	14	160	33	29	NA	NA	NA	NA	NA	intensive care	yes	unclear	adults	yes
Obara et al	2011	LightCycler SeptiFast Test MGRADE	BACTEC 9000	78	11	56	10	1	NA	NA	NA	NA	NA	mixed population	yes	unclear	adults	unclear
Ozkaya-Parlakay et al	2014	LightCycler SeptiFast Test MGRADE	NA	79	19	55	0	5	NA	NA	NA	NA	NA	mixed population	no	unclear	NA	unclear
Paolucci et al	2013	LightCycler SeptiFast Test MGRADE	BacT/Alert 3D	437	0.39†	0.96‡	0.67§	0.87¶	201	NA	NA	NA	NA	immunosuppression	yes	unclear	NA	yes
Pasqualini et al	2012	LightCycler SeptiFast Test MGRADE	BACTEC 9240	391	34	289	26	42	391	34	289	26	42	mixed population	unclear	unclear	adults	yes
Rath et al	2012	LightCycler SeptiFast	BACTEC 9240	225	57	110	35	23	NA	NA	NA	NA	NA	intensive care	unclear	yes	adults	yes

		Test MGRADE																
Ratzinger et al	2016	LightCycler SeptiFast Test MGRADE	BacT/Alert 3D	NA	NA	NA	NA	NA	220	31	156	18	15	mixed population	no	unclear	adults	yes
Regueiro et al	2010	LightCycler SeptiFast Test MGRADE	BacT/Alert 3D	106	16	78	1	11	NA	NA	NA	NA	NA	intensive care	no	unclear	adults	yes
Regueiro et al	2010	LightCycler SeptiFast Test MGRADE	BacT/Alert 3D	106	25	77	2	2	NA	NA	NA	NA	NA	intensive care	no	unclear	adults	yes
Reyna Figueroa et al	2019	LightCycler SeptiFast Test MGRADE	NA	20	4	12	4	0	20	4	12	4	0	intensive care	no	unclear	newborn-infant	yes
Rodrigues et al	2019	LightCycler SeptiFast Test MGRADE	BACTEC FX	NA	NA	NA	NA	NA	200	28	144	17	11	mixed population	no	yes	adults	NA
Rogina et al	2014	SepsiTest	BACTEC 9240	NA	NA	NA	NA	NA	23	3	14	6	0	emergency department	no	yes	adults	yes
Schaub et al	2014	LightCycler SeptiFast Test MGRADE	BacT/Alert 3D	NA	NA	NA	NA	NA	110	16	78	5	11	emergency department	yes	no	NA	yes
Schreiber et al	2013	LightCycler SeptiFast Test MGRADE	BACTEC 9240/9120	50	3	33	4	10	50	3	33	4	10	intensive care	yes	unclear	NA	yes
Schreiber et al	2013	SepsiTest	BACTEC 9240/9120	50	4	35	2	9	50	4	35	2	9	intensive care	yes	unclear	NA	yes
Schreiber et al	2013	VYOO	BACTEC 9240/9120	50	4	36	1	9	50	4	36	1	9	intensive care	yes	unclear	NA	yes
Sitnik et al	2014	LightCycler SeptiFast	BACTEC 9240	114	13	87	10	4	114	13	87	10	4	mixed population	yes	unclear	NA	yes

		Test MGRADE																
Stralin et al	2020	IRIDICA BAC BSI	NA	NA	NA	NA	NA	NA	1460	150	1023	224	63	mixed population	yes	no	NA	yes
Suberviola et al	2016	LightCycler SeptiFast Test MGRADE	BACTEC FX 200	119	30	55	27	7	NA	NA	NA	NA	NA	intensive care	yes	no	NA	yes
Tafelski et al	2015	LightCycler SeptiFast Test MGRADE	BACTEC	78	7	55	11	5	78	7	55	11	5	intensive care	yes	yes	NA	yes
Tat Trung et al	2018	LightCycler SeptiFast Test MGRADE	BD BACTEC 9120	110	19	63	12	16	110	19	63	12	16	mixed population	no	no	NA	unclear
Tkadlec et al	2019	UMD-SelectNA	Bactec FX	848	123	556	122	47	NA	NA	NA	NA	NA	mixed population	no	unclear	NA	
Tran et al	2012	LightCycler SeptiFast Test MGRADE	NA	NA	NA	NA	NA	NA	50	13	23	11	3	intensive care	yes	unclear	NA	NA
Tröger et al	2016	LightCycler SeptiFast Test MGRADE	NA	214	45	94	65	10	NA	NA	NA	NA	NA	NA	no	unclear	newborn-infant	yes
Tsalik et al	2010	LightCycler SeptiFast Test MGRADE	BacT/Alert	306	40	201	13	52	306	40	201	13	52	emergency department	no	unclear	adults	yes
Varani et al	2009	LightCycler SeptiFast Test MGRADE	BacT/Alert 3D	154	22	101	13	18	NA	NA	NA	NA	NA	immunosuppression	yes	unclear	NA	yes
Wallet et al	2009	LightCycler SeptiFast Test MGRADE	BacT/Alert	100	4	79	11	6	72	5	53	8	6	intensive care	yes	no	NA	yes
Wang et al	2015	REBA Sepsis-ID	BacT/Alert 3D	882	115	617	123	27	882	115	617	123	27	emergency department	no	yes	adults	yes

Wellinghausen et al	2009	SepsiTest	BACTEC 9240	342	47	247	41	7	187	28	122	31	6	mixed population	no	unclear	NA	yes
Westh et al	2009	LightCycler SeptiFast Test MGRADE	BacT/Alert	613	51	382	135	45	NA	NA	NA	NA	NA	NA	yes	yes	NA	yes
Zboromyrska et al	2019	MagicPlex Sepsis Test	BACTEC 9240	809	45	574	58	132	NA	NA	NA	NA	NA	mixed population	no	unclear	NA	yes
Ziegler et al	2016	MagicPlex Sepsis Test	BACTEC	696	74	374	194	54	696	74	374	194	54	emergency department	yes	yes	adults	yes

TN=true negative. TP=true positive. FP=false postivive. FN=false negative. NA=not available data for statistical analysis
*2 blood culture sets=2 aerobic and 2 anaerobic bottles
†sensitivity
‡ specificity
§ positive predictive value
¶ negative predictive value

Studies excluded at full-text screening stage with brief reasons

Author	DOI/PMID/URL	Reason for exclusion*
Yuan, L.	10.1093/labmed/lmad046.	not available
Alonso-Cadenas, J. A.	10.1136/archdischild-2024-327367	not available
O. Y. Kutsevalova	10.51620/0869-2084-2022-67-2-101-105	not available
R. H. Edgar	10.1117/12.2510210	not available
N. Ambrosio	10.1002/central/CN-02361916/full	not available
L. T. Nguyen	10.21161/mjm.211331	not available
F. Pirali	PMID: 8366820	not available
N. A. Pathare	PMID: 3244121	not available
G. Dimopoulos	PMID: 17523273	not available
T. M. Bingold	https://www.researchgate.net/publication/295954384_Clinical_utility_of_a_new_PCR-based_assay_for_rapid_pathogen_detection_SeptiFast_R_in_patients_with_clinical_sepsis_compared_to_standard_blood_culture	not available
M. Suenaga	10.1016/j.jamcollsurg.2020.12.001	not available
Y. Li	10.1166/jmihi.2018.2281	not available
F. Wallet	10.2174/187152611796504845	not available
S. Sheikhbahaei	10.2174/1871530319666190211163245	not available
S. Lata	https://www.embase.com/search/results?subaction=viewrecord&id=L2018900580&from=export	not available
M. J. O'Dwyer	10.1016/j.cmi.2016.11.010	data from a previous article
I. Ziegler	10.1186/1471-2334-14-155	data from a previous article
A. Markota	10.1177/0300060517719768	data from a previous article
G. Warhurst	10.3310/hta19350	data from a previous article
W. P. Lin	10.3760/cma.j.cn501225-20230803-00036	non-english article
Lou, C. Y.	10.7499/j.issn.1008-8830.2311079.	non-english article
E. Goudarzi	https://www.embase.com/search/results?subaction=viewrecord&id=L373925273&from=export	non-english article
S. Hanna	https://www.embase.com/records?subaction=viewrecord&id=L36806844	non-english article
T. Arishima	10.3314/jjmm.47.283	non-english article
A. Gholami	https://www.embase.com/search/results?subaction=viewrecord&id=L608667154&from=export	non-english article
E. Čeljuska-Tošev	https://www.embase.com/search/results?subaction=viewrecord&id=L358662708&from=export	non-english article
M. L. Celadilla	10.1002/central/CN-02333614/full	non-english article
B. Kim	10.3947/ic.2011.43.3.240	non-english article
G. Erdoğan	10.5578/mb.20229705	non-english article
A. Ferroni	10.1016/s0929-693x(07)80041-8	non-english article
M. Ferreira	10.20344/amp.8493	non-english article
S. Afsharpaiman	https://www.embase.com/search/results?subaction=viewrecord&id=L2002440431&from=export	non-english article
D. A. Popov	PMID: 22164420	non-english article
Z. Qiao	PMID: 16464377	non-english article
E. Torres-Martos	10.1016/j.eimc.2012.09.012	non-english article

P. Diamante	https://www.embase.com/search/results?subaction=viewrecord&id=L359503759&from=export	non-english article
L. Bayer	10.1055/s-2000-9610	non-english article
Y. V. Ostankova	https://www.embase.com/search/results?subaction=viewrecord&id=L625847863&from=export	non-english article
A. Shiralinezhad	https://www.cabdirect.org/globalhealth/abstract/20203359636	non-english article
G. Sobol	https://www.embase.com/search/results?subaction=viewrecord&id=L355779719&from=export	non-english article
Z. Vacková	https://www.embase.com/search/results?subaction=viewrecord&id=L613554915&from=export	non-english article
U. Lodes	10.1055/s-0028-1098776	non-english article
C. Loïez	10.1007/s13546-013-0664-4	non-english article
R. B. Luo	10.3760/cma.j.cn501120-20201017-00440	non-english article
L. Martinez-Lamas	10.1016/j.eimc.2011.03.014	non-english article
E. Mimaroglu	10.5578/mb.61826	non-english article
W. Storm	10.1055/s-2008-1034426	non-english article
E. O. Kotova	10.20996/1819-6446-2023-02-03	non-english article
T. Şimşek Bozok	10.5578/mb.20219708	non-english article
Ramanathan, A.	10.1016/j.heliyon.2024.e34538	tat >12 hours
Zhang, D.	10.3389/fmicb.2024.1384166	tat >12 hours
Yu, J.	10.3389/fcimb.2024.1338861	tat >12 hours
Y. Fu	10.1128/spectrum.00270-22	tat >12 hours
P. Trabasso	10.1007/s11046-014-9830-9	tat >12 hours
I. Garcia-Gudino	10.1007/s00431-017-3036-3	tat >12 hours
J. Y. Chien	10.1128/spectrum.00746-22	tat >12 hours
W. Tang	10.3389/fimmu.2021.696403	tat >12 hours
L. W. Roberts	10.1099/mgen.0.000530	tat >12 hours
E. Schulz	10.1093/ofid/ofac393	tat >12 hours
R. A. Komorowski	10.1093/ajcp/59.1.56	tat >12 hours
C. Lin	10.1016/j.pan.2022.07.006	tat >12 hours
T. Gosiewski	10.1007/s10096-016-2805-7	tat >12 hours
N. E. Connor	10.1136/bmjgh-2022-009706	no device information
S. Emler	10.1093/clinids/20.4.772	no device information
C. Edge	10.1016/j.jinf.2016.07.016	no device information
V. D'Acremont	10.1056/NEJMoa1214482	no device information
J. Sun	10.21037/apm-22-1071	no device information
S. K. Saha	10.1016/s0140-6736(18)31127-9	no device information
R. Sklavou	10.1016/j.clinbiochem.2012.07.088	no device information
L. Sun	10.3389/fcimb.2022.905132	no device information
L. Ward	10.1007/s10096-019-03581-4	no index test was performed
Munir Abu-Helalah	10.3390/vaccines11091396	not commercial device
Agudelo-Pérez, S.	10.3390/tropicalmed9070152.	not commercial device
Aralar, A	10.1016/j.jmoldx.2024.01.013.	not commercial device
Aralar, A	10.1101/2023.09.07.23295215.	not commercial device
Arvay M. L.	10.1016/S2214-109X(22)00244-3	not commercial device
Y. Cao	10.3389/fcimb.2023.1144625	not commercial device
Zhao X.	10.3389/fcimb.2023.1131258.	not commercial device
Yang H.	10.1186/s12879-024-09236-w.	not commercial device

Fu Y.	10.2147/IDR.S379582	not commercial device
Lieu A.	10.1128/jcm.01518-23	not commercial device
Wang, R.	10.3390/bios13100910	not commercial device
Li, X.	10.3389/fcimb.2023.1170687	not commercial device
Hong, M.	10.1111/jcmm.17651	not commercial device
Miyakoshi, A.	10.1038/s41598-023-50864-0.	not commercial device
Song, J.	10.1111/1751-7915.14380	not commercial device
Jiang, S.	10.3389/fcimb.2024.1358801	not commercial device
Iyer, V.	10.1128/jcm.01498-23.	not commercial device
Han, D.	10.1016/j.jinf.2024.106166.	not commercial device
Ishikawa, T.	10.1016/j.jiac.2023.11.024.	not commercial device
Kawai, Y.	10.1016/j.mimet.2024.106982.	not commercial device
Sharma, A.	10.1016/j.diagmicrobio.2024.116398.	not commercial device
Lengert, A. V. H.	10.1016/j.diagmicrobio.2024.116426	not commercial device
Yin, S.	10.2147/idr.S439683	not commercial device
E. Jordana-Lluch	10.1371/journal.pone.0062108	not commercial device
O. Esparcia	10.1016/j.diagmicrobio.2010.10.022	not commercial device
M. schur	10.1016/j.cimid.2007.10.005	not commercial device
P. Athamanolap	10.1021/acs.analchem.7b02809	not commercial device
M. Ashrafi	10.18869/acadpub.cmm.1.1.35	not commercial device
C. Zhang	10.1038/s41598-018-31200-3	not commercial device
M. Y. Yuhana	10.3390/tropicalmed7050077	not commercial device
Y. Xiao	10.3389/fcimb.2019.00361	not commercial device
Y. Uejima	10.1128/spectrum.00198-22	not commercial device
C. Y. Turenne	10.1128/jcm.37.6.1846-1851.1999	not commercial device
Y. Tong	10.1155/2022/2549413	not commercial device
J. Nölling	10.1128/mBio.00345-16	not commercial device
H. Niimi	10.1038/srep12543	not commercial device
P. Badiee	PMID: 20565365	not commercial device
C. Azzari	10.1016/j.vaccine.2013.09.055	not commercial device
M. Flahaut	10.1128/jcm.36.2.395-401.1998	not commercial device
A. Portillo	10.3390/pathogens9030189	not commercial device
A. Ganguli	10.1073/pnas.2209607119	not commercial device
B. Forsyth	10.3390/bios11080288	not commercial device
Z. Cheng	10.12659/MSM.937041	not commercial device
L. H. Chen	10.1177/0009922809333972	not commercial device
S. Guiducci	10.1371/journal.pone.0212922	not commercial device
M. Guembe	10.1128/JCM.02414-12	not commercial device
J. L. Goodman	10.1016/s0002-9343(99)80097-7	not commercial device
F. Firoozeh	10.2174/1874285801913010101	not commercial device
A. M. Castro	10.1007/s00436-002-0679-3	not commercial device
S. H. Kim	10.1128/jcm.00027-08	not commercial device
L. Hasseine	10.1016/j.jinf.2015.04.005	not commercial device
Y. Guo	10.1007/s11046-015-9977-z	not commercial device

Y. C. Chuang	10.1097/CCM.0000000000003264	not commercial device
Y. C. Chuang	10.1097/CCM.0b013e3182515190	not commercial device
M. Khlif	10.1016/j.mycmed.2007.10.001	not commercial device
F. Gouriet	10.1007/s10096-012-1599-5	not commercial device
H. Einsele	10.1128/jcm.35.6.1353-1360.1997	not commercial device
R. Fisa	10.1016/s0035-9203(02)90075-1	not commercial device
N. A. A. H. Hassanin	10.3855/jidc.12101	not commercial device
R. M. C. Checa	10.1097/cce.0000000000000707	not commercial device
J. Faber	10.1111/j.1439-0507.2008.01565.x	not commercial device
D. F. Kinane	10.1111/j.1600-051X.2005.00741.x	not commercial device
H. Koh	10.1016/j.jiac.2016.08.001	not commercial device
H. H. Al-Ajlan	PMID: 21630571	not commercial device
Ahlstr. E	10.1111/apm.12182	not commercial device
C. Aguiar	10.1111/j.1365-3156.2011.02936.x	not commercial device
S. Agha Kuchak Afshari	10.1016/j.mycmed.2017.09.002	not commercial device
J. L. Camacho-Cardoso	10.24875/gmm.17002535	not commercial device
R. J. Abduzzahra	https://www.embase.com/search/results?subaction=viewrecord&id=L2019588881&from=export	not commercial device
A. Nakamura	10.1097/SHK.0b013e31819716fa	not commercial device
R. P. H. Peters	10.1128/jcm.01056-07	not commercial device
J. Zhang	10.1186/s12879-022-07793-6	not commercial device
D. Zhang	10.1002/jcla.24686	not commercial device
U. H. Tirodker	10.1038/sj.jp.7210868	not commercial device
A. K. Yadav	PMID: 16085969	not commercial device
S. Zelenin	10.1007/s10529-014-1734-8	not commercial device
T. T Zhang	10.1038/s41598-021-87824-5	not commercial device
F. B. Rowther	10.1128/JCM.00418-07	not commercial device
A. Rohit	10.4103/0971-5916.178613	not commercial device
D. Ren	10.1186/s12879-021-06934-7	not commercial device
L. C. Reis	10.1016/j.joen.2016.05.013	not commercial device
L. C. Reis	10.1111/odi.12792	not commercial device
J. F. Ramos	10.1007/s10096-021-04361-9	not commercial device
S. Rahn	10.1016/j.ijmm.2016.10.003	not commercial device
A. Nakamura	10.1128/jcm.01700-09	not commercial device
J. A. Jordan	10.1016/s1525-1578(10)60015-3	not commercial device
S. Dutta	10.1001/archpediatrics.2008.513	not commercial device
M. R. Drummond	10.1371/journal.pntd.0010603	not commercial device
S. Decuypere	10.1371/journal.pntd.0004470	not commercial device
E. De Vitis	10.1093/trstmh/trab107	not commercial device
D'Ávila	10.1371/journal.pone.0208133	not commercial device
R. Bu	10.1099/jmm.0.45856-0	not commercial device
J. R. Bogner	10.3109/00365549709035898	not commercial device

I. Bogdan	10.3390/antibiotics11040437	not commercial device
W. Z. Zhang	10.3748/wjg.v7.i2.289	not commercial device
E. Lahtni	10.1007/s10096-006-0225-9	not commercial device
N. Wellinghausen	10.1099/jmm.0.007906-0	not commercial device
A. Ohlin	10.1159/000334655	not commercial device
M. C. Mora	10.1645/ge-549r.1	not commercial device
M. J. Marin	10.4317/medoral.20842	not commercial device
A. Martinez-Gamboa	10.1371/journal.pntd.0009215	not commercial device
I. R. Makhoul	PMID: 19344010	not commercial device
D. A. Midan	10.1080/14767058.2016.1219994	not commercial device
M. Millar	10.3310/hta15070	not commercial device
J. C. Moisi	10.4269/ajtmh.15-0431	not commercial device
E. A. Morad	10.1155/2020/8889086	not commercial device
M. S. Moreira-Oliveira	10.1007/s10096-005-0041-7	not commercial device
S. M. Morrissey	10.1007/s10096-017-2938-3	not commercial device
L. Savarrio	10.1016/j.jdent.2004.09.008	not commercial device
Y. Shachor-Meyouhas	10.1097/MPH.0b013e31829eec78	not commercial device
S. Shang	10.1203/01.Pdr.0000169580.64191.8b	not commercial device
H. J. Shen	10.1021/acsami.6b06671	not commercial device
S. H. Shinkafi	10.4314/ahs.v19i3.11	not commercial device
K. M. Song	10.1097/BPO.0b013e3181982533	not commercial device
Y. Song	10.1371/journal.pone.0219086	not commercial device
N. Srisawat	10.1371/journal.pone.0143367	not commercial device
I. Stranieri	10.1590/S1678-9946201860061	not commercial device
D. L. Valle	10.1590/s0074-02762010000200001	not commercial device
L. M. Vanhee	10.1128/jcm.00035-10	not commercial device
M. R. Vutukuru	10.1016/j.mimet.2016.10.008	not commercial device
Y. Wouters	10.1111/1751-7915.13491	not commercial device
A. Lau	10.1128/jcm.01650-09	not commercial device
K. Lin	10.1128/spectrum.01378-22	not commercial device
H. Obrucova	10.1128/jcm.00118-16	not commercial device
R. van Haeften	10.1016/s0732-8893(03)00129-9	not commercial device
C. Izquierdo	10.1017/s0950268820002708	not commercial device
T. G. Laffler	10.1128/JCM.00876-13	not commercial device
M. I. Lara	10.1177/1129729820934354	not commercial device
C. Lass-Florl	10.1111/j.1439-0507.2005.01104.x	not commercial device
M. T. Lin	10.1086/322631	not commercial device
C. L. Liu	10.1016/j.arcped.2013.11.015	not commercial device
Y. Maaroufi	10.1128/jcm.41.7.3293-3298.2003	not commercial device
I. R. Makhoul	10.1128/jcm.43.9.4823-4825.2005	not commercial device
F. Modaresi	10.21276/ambi.2019.06h.1.oa05	not commercial device
P. Siondalski	PMID: 15702912	not commercial device
J. Sleigh	10.1007/s001340100981	not commercial device
M. L. DeMarco	10.1016/j.cll.2013.03.013	not commercial device
K Greisen	10.1128/jcm.32.2.335-351.1994	not commercial device
G. Greub	10.1016/j.amjmed.2004.12.014	not commercial device
G. N. Becker	10.1016/j.mimet.2021.106182	not commercial device
I. Barišić	10.1099/jmm.0.000192	not commercial device

M. B. Rahmati	https://irisweb.ir/rdsm_jarticle_list.php?slc_lang=fa&sid=1&mod=jarticle_profile&jart_id=81182&rds_id=	non-english article
O. A. Eltantawy	10.21608/nrmj.2021.178298	not commercial device
L. Chen	10.1128/spectrum.01195-22	not commercial device
I. Ziegler	10.1371/journal.pone.0224656	not commercial device
A. J. Heeroma	10.3390/s20154182	not commercial device
P-E. Fournier	10.1086/653675	not commercial device
S. E. Gawhary	10.1093/tropej/fmv066	not commercial device
L. Klingspor	10.1111/j.1469-0691.2006.01498.x	not commercial device
Qu H.	10.1182/blood-2022-162289.	non-original research article
Yao, L.	10.1182/blood-2023-184584	non-original research article
Kurtipek, F. B.	10.1016/j.htct.2023.09.079.	non-original research article
P. Orszag	10.1128/jcm.02493-13	non-original research article
P. Gaibani	10.1016/s0924-8579(09)70559-x	non-original research article
C. S. P. Gomes	10.1016/j.ijid.2014.03.1177	non-original research article
R. Plettig	https://www.embase.com/search/results?subaction=viewrecord&id=L70639590&from=export	non-original research article
D. Pak	10.1097/inf.0000000000002643	non-original research article
J. Tkadlec	10.1002/mbo3.1007	non-original research article
J. S. Hassan	10.51248/.v41i2.802	non-original research article
Y. W. Han	10.1016/j.ajog.2011.10.076	non-original research article
A. Goehler	https://www.embase.com/search/results?subaction=viewrecord&id=L72056316&from=export	non-original research article
N. Galindo-Sevilla	10.3109/14767058.2014.924236	non-original research article
M. Karrasch	10.1515/cclm-2017-1110	non-original research article
M. Kami	10.1038/sj.bmt.1704997	non-original research article
X. A. Y. Han	10.1128/9781555815455.ch20	non-original research article
M. Paolucci	10.1099/jmm.0.003848-0	non-original research article
R. Arjun	10.5005/jp-journals-10071-24329	non-original research article
C. Chaisomboon	10.1515/cclm-2015-5005	non-original research article
J. Gutierrez	https://www.embase.com/search/results?subaction=viewrecord&id=L623264036&from=export	non-original research article
B. Etchebarne	10.1111/acem.13424	non-original research article
S. Hassan	10.1111/ajt.13898	non-original research article
H. S. Kim	https://www.embase.com/search/results?subaction=viewrecord&id=L70427212&from=export	non-original research article
M. Katsiari	10.1111/j.1469-0691.2011.03558.x	non-original research article
E. Halász	10.1515/cclm-2012-0427	non-original research article
J. Guinea	10.1111/j.1469-0691.2011.03558.x	non-original research article
R. Greco	https://www.embase.com/search/results?subaction=viewrecord&id=L70772009&from=export	non-original research article
R. Greco	https://www.embase.com/search/results?subaction=viewrecord&id=L71760395&from=export	non-original research article
R. Greco	10.1038/bmt.2012.36	non-original research article
E. Gimeno	https://www.embase.com/search/results?subaction=viewrecord&id=L70711982&from=export	non-original research article
A. Cortegiani	10.1007/s15010-009-1004-1	non-original research article

D. Clerici	https://www.embase.com/search/results?subaction=viewrecord&id=L70013228&from=export	non-original research article
A. Chaidaroglou	https://www.embase.com/search/results?subaction=viewrecord&id=L71251669&from=export	non-original research article
A. Chaidaroglou	10.1016/j.healun.2011.01.438	non-original research article
A. Chaidaroglou	10.1111/j.1469-0691.2012.03802.x	non-original research article
A. Chaidaroglou	10.1186/cc9133	non-original research article
M. Avolio	10.1111/j.1469-0691.2010.03239.x	non-original research article
K. Grif	10.1007/s00508-012-0159-4	non-original research article
A. Fouad	10.1111/j.1469-0691.2010.03239.x	non-original research article
P. S. Fanourgiakis	PMID: 22143022	non-original research article
M. E. Falagas	10.1097/CCM.0b013e31817045e5	non-original research article
T. Higuchi	10.1097/INF.0000000000003651	non-original research article
M. D. Gupta	10.4103/0255-0857.180351	non-original research article
A. J. Kindo	10.1111/j.1439-0507.2011.02092.x	non-original research article
L. Knabl	10.1016/j.mimet.2015.12.001	non-original research article
A. Kortgen	10.1007/s15010-009-1004-1	non-original research article
K. Amin-Desai	https://www.embase.com/search/results?subaction=viewrecord&id=L613102253&from=export	non-original research article
B. Adamik	10.1007/s15010-011-0175-8	non-original research article
S. Fuchs	10.3390/jof5030086	non-original research article
A. Kalenka	https://www.embase.com/search/results?subaction=viewrecord&id=L70190974&from=export	non-original research article
E. Gimeno	https://www.embase.com/search/results?subaction=viewrecord&id=L70013229&from=export	non-original research article
Y. Reers	10.1016/j.diagmicrobio.2016.04.024	non-original research article
Y. Zhao	https://clinicaltrials.gov/show/NCT05190861 2022	non-original research article
P. Badiee	https://www.embase.com/records?subaction=viewrecord&id=L71976580	non-original research article
F. Bloos	10.1186/cc8065	non-original research article
U. Nawrot	10.1111/j.1439-0507.2011.02092.x	non-original research article
M. Paolucci	10.1099/jmm.0.003848-0	non-original research article
U. H. Tirodker	10.1007/978-3-642-18480-2_19	non-original research article
C. Tavano	10.1128/JCM.00175-18	non-original research article
R. Zhang	10.1016/S1413-8670(11)70241-0	non-original research article
N. L. Zitterkopf	10.1309/lms6d81lgcnpnwwx	non-original research article
Y. Niederbracht	10.1016/j.ijmm.2013.08.004	non-original research article
P. Ranjan	https://www.embase.com/search/results?subaction=viewrecord&id=L71239325&from=export	non-original research article
P. D. Navalkele	https://www.embase.com/search/results?subaction=viewrecord&id=L71523425&from=export	non-original research article
E. L. Tsalik	https://www.embase.com/search/results?subaction=viewrecord&id=L70190972&from=export	non-original research article
T. Pleskova	10.1515/CCLM.2011.519	non-original research article
A. M. Planes	10.1016/j.medcli.2009.02.015	non-original research article
V. Pavone	https://www.embase.com/search/results?subaction=viewrecord&id=L70013222&from=export	non-original research article

S. Johri	10.1016/S0377-1237(05)80071-1	non-original research article
O. Janae Martin	https://www.embase.com/search/results?subaction=viewrecord&id=L70758213&from=export	non-original research article
S. Jain	10.1179/2047773214y.0000000133	non-original research article
C. Disque	https://www.embase.com/search/results?subaction=viewrecord&id=L70300974&from=export	non-original research article
F. Dell'Omo	10.1515/jpm-2015-2003	non-original research article
C. F. Burger	https://www.embase.com/search/results?subaction=viewrecord&id=L362409105&from=export	non-original research article
L. Bianchi	https://www.embase.com/search/results?subaction=viewrecord&id=L71665885&from=export	non-original research article
V. Bhat	https://www.cochranelibrary.com/central/doi/10.1002/central/CN-01654946/full	non-original research article
N. D. Beyda	https://www.embase.com/search/results?subaction=viewrecord&id=L358070526&from=export	non-original research article
A. E. Nieman	10.1111/j.1469-0691.2011.03559.x	non-original research article
A. Berger	10.1055/s-0030-1261288	non-original research article
B. Barzoloski-O'Connor	10.1097/01.CCN.0000451020.07574.3c	non-original research article
D. Zhang	10.1111/resp.13699	non-original research article
I. Zeller	10.1111/myc.12975	non-original research article
Y. M. C. Yang	10.1186/cc10400	non-original research article
X. Xiang	10.1097/01.HS9.0000852292.38263.b8	non-original research article
A. Thornberg	https://www.embase.com/search/results?subaction=viewrecord&id=L621784397&from=export	non-original research article
E. A. Idelevich	10.1016/j.ijmm.2011.08.002	non-original research article
L. E. Lehmann	10.1007/s15010-009-1004-1	non-original research article
E. Leitner	10.1016/j.mimet.2012.12.012	non-original research article
J. Linares	10.1086/511885	non-original research article
U. Lodes	10.1186/s40635-018-0201-6	non-original research article
U. Lodes	10.1016/j.ijmm.2011.08.002	non-original research article
A. Lupetti	10.1099/jmm.0.069161-0	non-original research article
M. Mai	10.1007/978-1-4939-1776-1_14	non-original research article
J. Mancilla	10.1515/jpm-2013-2001	non-original research article
D. Maneg	10.1515/cclm-2016-0723	non-original research article
T. Mann Ben Yehudah	10.1186/s13054-016-1208-6	non-original research article
A. Markota	10.1007/s00134-013-3095-5	non-original research article
E. M. Marlowe	10.1128/9781555816834.ch28	non-original research article
S. L. Martin	10.1186/s40635-017-0149-y	non-original research article
A. R. Mohee	10.1111/bju.12137	non-original research article
Z. Oikonomopoulou	10.1093/ofid/ofz360.340	non-original research article
C. Orasch	10.1111/j.1469-0691.2009.02858.x	non-original research article
F. Ortiz Ibarra	https://www.embase.com/search/results?subaction=viewrecord&id=L70925951&from=export	non-original research article
S. Owusu-Ofori	10.1111/trf.12401	non-original research article
A. Santos Bouza	10.1007/s00134-010-2000-8	non-original research article
N. Schaub	10.1111/j.1469-0691.2009.02858.x	non-original research article
H. Schirmer	10.1111/myc.12380	non-original research article
R. Sitnik	https://www.embase.com/search/results?subaction=viewrecord&id=L70655750&from=export	non-original research article

G. Sobol-Milejska	10.1002/pbc.24719	non-original research article
J. Sóki	10.1111/j.1469-0691.2010.03239.x	non-original research article
J. Steinmann	10.1016/j.ijmm.2011.08.002	non-original research article
T. Stetzler	10.1093/ofid/ofz360.830	non-original research article
A. Szekely	10.1111/myc.12674	non-original research article
K. Van De Groep	10.1186/s40635-018-0201-6	non-original research article
G. Vrioni	10.1099/jmm.0.037127-0	non-original research article
G. Vrioni	10.1111/j.1469-0691.2011.03559.x	non-original research article
G. Vrioni	10.1111/j.1469-0691.2012.03801.x	non-original research article
G. Vrioni	https://www.embase.com/search/results?subaction=viewrecord&id=L72086888&from=export	non-original research article
G. Vrioni	10.1111/myc.12380	non-original research article
G. Vrioni	10.1111/myc.12674	non-original research article
E. Wade	10.1177/1751143718772957	non-original research article
S. Wolf	10.1159/000518417	non-original research article
J. Mancilla-Ramirez	https://www.embase.com/search/results?subaction=viewrecord&id=L619372980&from=export	non-original research article
M. N. Massi	10.1016/j.ijmm.2005.01.003	non-original research article
A. R. Mohee	10.1111/bju.12136	non-original research article
H. Obara	10.1089/sur.2009.9946	non-original research article
P. Orszag	10.1016/j.ijmm.2013.08.004	non-original research article
S. Sachse	https://www.webofscience.com/wos/woscc/full-record/WOS:000263264200019?SID=EUW1ED0F47PWTLdzkvnkW4MOuLjnX	non-original research article
T. Schmoch	10.1097/md.0000000000026403	non-original research article
M. Shennan	https://www.embase.com/search/results?subaction=viewrecord&id=L70655778&from=export	non-original research article
I. Vives	10.3109/14767058.2012.679162	non-original research article
J. Wagner	https://www.embase.com/search/results?subaction=viewrecord&id=L71659789&from=export	non-original research article
M. S. Wu	10.1016/j.mimet.2017.06.019	non-original research article
M. C. Barbanti	10.1038/bmt.2015.30	non-original research article
P. Bernaschi	10.1111/j.1469-0691.2010.03239.x	non-original research article
T. Bingold	10.1007/s15010-009-1004-1	non-original research article
B. P. Blanco	10.1542/peds.146.1_MeetingAbstract.153	non-original research article
F. Bloos	10.1007/s15010-011-0175-8	non-original research article
F. Bloos	10.1007/s15010-009-1004-1	non-original research article
E. Burdino	10.1111/j.1469-0691.2012.03802.x	non-original research article
C. Disqué	10.1007/s15010-011-0175-8	non-original research article
M. Nabili	10.1111/j.1439-0507.2012.02206.x	non-original research article
T. Nejtek	10.1007/s15010-019-01341-2	non-original research article
J. C. Palomares	10.1111/j.1469-0691.2009.02858.x	non-original research article
S. M. Raineri	10.1186/cc7540	non-original research article
E. Raukas	10.1111/j.1469-0691.2012.03802.x	non-original research article
C. Rodrigues	10.1186/cc12926	non-original research article
W. Rozemeijer	10.1111/j.1469-0691.2011.03557.x	non-original research article
S. Al Johani	10.1111/j.1469-0691.2010.03239.x	non-original research article
R. A. Ammann	10.1007/s00520-007-0235-x	non-original research article
A. Asundi	10.1093/ofid/ofaa417.463	non-original research article
A. Conen	10.1111/j.1469-0691.2009.02858.x	non-original research article
C. Kühn	10.1007/s15010-011-0175-8	non-original research article

R. Krause	10.1111/j.1469-0691.2010.03154.x	non-original research article
M. Kotnik	10.1111/j.1469-0691.2010.03239.x	non-original research article
A. Kadoom	10.1093/ofid/ofaa417.349	non-original research article
D. Greenwood	10.1016/0140-6736(93)92875-t	non-original research article
S. H. Ahmed	10.1186/1756-0500-2-76	non-original research article
S. Omar	10.7196/SAJCC.2021.v37i3.495	non-original research article
J. Serra	https://www.embase.com/search/results?subaction=viewrecord&id=L70925915&from=export	non-original research article
M. Skvarc	10.1016/j.ijmm.2012.08.002	non-original research article
S. Varani	10.1016/s0924-8579(07)71249-9	non-original research article
E. A. Idelevich	10.3238/arztebl.2018.0822	non-original research article
J. M. Molina	10.1016/S0213-005X(08)76544-3	non-original research article
M. J. G. Vehreschild	10.1159/000360634	non-original research article
B. L. Wasilauskas	https://www.embase.com/search/results?subaction=viewrecord&id=L27341486&from=export	non-original research article
D. J. Ecker	10.1038/nrmicro1918	non-original research article
J. Klastersky	10.1016/j.ijantimicag.2007.06.012	non-original research article
A. Irwin	https://www.researchgate.net/profile/Adam-Irwin/publication/320741016_The_Diagnosis_of_Serious_Bacterial_Infections_in_the_Children's_Emergency_Department/links/5adff628aca272fdaf8b566d/The-Diagnosis-of-Serious-Bacterial-Infections-in-the-Childrens-Emergency-Department.pdf	non-original research article
J. Steinmann	10.1128/jcm.00478-16	non-original research article
D. Marco	10.1007/s00431-016-2745-3	non-original research article
D. Milzman	10.1097/01.ccm.0000440059.14959.60	non-original research article
M. S. Moore	https://www.embase.com/search/results?subaction=viewrecord&id=L71660144&from=export	non-original research article
H. Imai	10.2147/idr.S345361	study population ≤10
Gillis, E. L. M. G.	10.1016/j.jmii.2024.03.003	target list <20
Zhao, Z.	10.1016/j.heliyon.2024.e27523.	target list <20
R. G. Maggi	10.1016/j.mimet.2020.106022	target list <20
N. T. Trung	10.1038/s41598-019-50150-y	target list <20
M. Fujimori	10.1186/1471-2431-10-53	target list <20
M. D. Zhao	10.3389/fcimb.2022.958858	target list <20
Y. C. Zhang	10.1016/s1995-7645(13)60032-2	target list <20
F. Bwanga	10.1186/s12879-015-0785-3	target list <20
P. Badiee	10.1186/s13052-022-01306-6	target list <20
P. Badiee	10.3855/jidc.807	target list <20
N. Andini	10.1373/clinchem.2018.290189	target list <20
C. Azzari	10.1099/jmm.0.2008/000935-0	target list <20
C. Azzari	10.1186/s13052-015-0189-4	target list <20
H. Frickmann	10.1016/j.actatropica.2018.12.004	target list <20
M. J. Hepburn	10.1086/506348	target list <20
P. Y. I. Tam	10.1371/journal.pone.0152253	target list <20
C. J. Tann	10.1371/journal.pone.0097259	target list <20
C. J. Tann	10.1136/archdischild-2017-312744	target list <20
R. Pradhan	10.1371/journal.pone.0047531	target list <20
J. Fortun	10.1093/jac/dku225	target list <20
S. Elsayed	10.5858/2001-125-0344-EOTCEL	target list <20

J. S. Hassan	10.25258/ijpqa.v9i01.11362	target list <20
I. Furtado	10.1590/s0036-46652014000100012	target list <20
T. Kakita	10.1371/journal.pntd.0009993	target list <20
F. Huber	10.3390/bios12110994	target list <20
S. J. Hackett	10.1136/adc.86.1.44	target list <20
B. E. Etchebarne	10.3389/fmicb.2017.02211	target list <20
S. Esposito	10.1097/INF.0b013e31825384ae	target list <20
M. Khilf	10.1111/j.1469-0691.2009.02762.x	target list <20
J. A. Crump	10.1128/JCM.05963-11	target list <20
F. G Correa	10.1590/s0100-879x2012007500082	target list <20
C. E. Corless	10.1128/JCM.39.4.1553-1558.2001	target list <20
L. O. Conterno	10.1086/520099	target list <20
L. Folgueira	10.1128/jcm.34.3.512-515.1996	target list <20
M. Ferranti	PMID: 30252925	target list <20
Y. Higashi	10.1038/s41598-020-62276-5	target list <20
M. Kudo	10.1007/s10156-008-0655-7	target list <20
A. Kumar	10.1016/S1567-1348(02)00090-4	target list <20
N. Ambrosio	10.1016/j.archoralbio.2018.11.025	target list <20
W. C. Albrich	10.1136/bmjopen-2014-005953	target list <20
I. A. Al-Zengena,	10.35124/bca.2020.20.1.1513	target list <20
N. E. Aikawa	10.1016/j.rbre.2015.08.004	target list <20
B. Allaouchiche	10.1016/s0163-4453(99)90049-x	target list <20
R. Plettig	10.1186/s40560-015-0116-1	target list <20
M. H. Nguyen	10.1093/cid/cis200	target list <20
R. E. Rothman	10.1086/345367	target list <20
M. Resti	10.1086/656579	target list <20
M. Resti	10.1016/j.clinthera.2009.06.010	target list <20
W. L. Reno	10.1177/000313480106700603	target list <20
J. Rello	10.1378/chest.09-0258	target list <20
T. Reier-Nilsen	10.1186/1471-2431-9-5	target list <20
M. G. Quiles	10.1186/s12879-015-1033-6	target list <20
S. Nagaraj	10.18683/germs.2017.1112	target list <20
J. A. Jordan	10.2353/jmoldx.2006.050138	target list <20
J. A. Jordan	10.1016/S1525-1578(10)60590-9	target list <20
X. Jiang	10.3389/fmicb.2016.00916	target list <20
Deshp	10.4103/0255-0857.90179	target list <20
A. de Zoysa	10.1099/jmm.0.042879-0	target list <20
J. Davis	10.1371/journal.pone.0136472	target list <20
S. Das	10.1186/s12866-016-0723-6	target list <20
P. A. Bryant	10.1128/JCM.42.7.2919-2925.2004	target list <20
B. V. Bhat	10.1007/s12098-015-1956-3	target list <20
Z. Straňák	10.1515/jpm-2021-0184	target list <20
S. Sampath	10.1007/s12098-015-1956-3	target list <20
M. S. Mehta	10.1016/j.diagmicrobio.2014.06.005	target list <20

H. S. Martins	10.3390/ijerph110201465	target list <20
R. R. Maude	10.1186/s12879-016-1886-3	target list <20
M. Meehan	10.1007/s10096-015-2496-5	target list <20
E. Samara	10.1542/peds.2019-1509	target list <20
M. E. Santolaya	10.1097/INF.0b013e31822a37d7	target list <20
S. Scharf	10.1093/mmy/myab045	target list <20
J. B. Searns	10.1093/JPIDS/PIZ040	target list <20
H. Seo	10.1111/myc.13319	target list <20
W. Shibata	10.1016/j.mimet.2022.106566	target list <20
W. P. Silva-Junior	10.3855/jidc.7474	target list <20
K. van de Groep	10.1007/s10096-019-03616-w	target list <20
S. C. Velaphi	10.1371/journal.pone.0214077	target list <20
J. Wagner	10.1111/eci.12126	target list <20
S. Wang	10.2147/ijn.S29629	target list <20
P. Ohlsson	10.1021/acs.analchem.6b00323	target list <20
v. d. Br, M.	10.1186/s13054-018-2010-4	target list <20
D. J. Isaacman	10.1542/peds.101.5.813	target list <20
N. Laforgia	10.1111/j.1651-2227.1997.tb14815.x	target list <20
V. Levterova	https://www.embase.com/search/results?subaction=viewrecord&id=L632774746&from=export	target list <20
C. F. Liu	10.1002/jcla.22256	target list <20
J. B. Liu	10.3855/jidc.8872	target list <20
G. Morace	10.1128/jcm.37.6.1871-1875.1999	target list <20
Y. Mitsuda	PMID: 22926071	target list <20
A. Fernández-Cruz	10.1128/JCM.02882-12	ineligible study population
F. Bloos	10.1016/j.jcrc.2012.07.011	ineligible study population
A. Lefort	10.1111/j.1469-0691.2012.03764.x	ineligible study population
C. Gumbinger	10.1016/j.jns.2012.11.014	ineligible study population
M. W. Eshoo	10.1128/jcm.01669-09	reference test other than blood culture
D. Lim	10.3390/pathogens11020111	reference test other than blood culture
J. Balks	10.1038/s41390-024-03448-1	sample other than whole blood
S. Cai	10.21037/jtd-24-400	sample other than whole blood
Mulet-Bayona J. V.	10.1111/myc.13776	sample other than whole blood
Lao H. Y.	10.3389/fmicb.2023.1324494	sample other than whole blood
Huang, J.	10.3389/fcimb.2023.1251509	sample other than whole blood
Liu, Q.	10.3389/fcimb.2023.1192931	sample other than whole blood
Zuo, Y.	10.3390/diagnostics13020323	sample other than whole blood
Liu, W.	10.3390/pathogens12050719	sample other than whole blood
Janani, R. S.	10.1016/j.ijmmb.2024.100708	sample other than whole blood
Kou, X. X.	10.3389/fmicb.2024.1338395.	sample other than whole blood
Wei, L. Y.	10.1016/j.heliyon.2024.e35802.	sample other than whole blood
L.E. Lehmann	10.1007/s00430-007-0063-0	sample other than whole blood
M. M. Hassan	10.1016/j.bios.2017.07.057	sample other than whole blood
E. A. Hassan	10.1016/j.ijid.2013.12.014	sample other than whole blood

G. Fidler	10.1186/s12879-018-3283-6	sample other than whole blood
T. J. Abram	10.1039/c9lc01212e	sample other than whole blood
Y. Zheng	10.1002/mbo3.1247	sample other than whole blood
P. Zhang	10.7717/peerj.9623	sample other than whole blood
K. Uchida	10.1007/s00776-009-1373-4	sample other than whole blood
H. de Graaf	10.3310/hta21480	sample other than whole blood
P. Badiee	PMID: 20716040	sample other than whole blood
N. Unsworth	10.1093/qjmed/hcm149	sample other than whole blood
J. Townsend	10.1093/ofid/ofv023	sample other than whole blood
S. Teeraputon	10.1016/j.nmni.2017.05.007	sample other than whole blood
M. Rozo	10.1371/journal.pntd.0008381	sample other than whole blood
M. R. Pingle	10.1128/jcm.00226-07	sample other than whole blood
J. Y. Zhao	10.1080/22221751.2022.2076612	sample other than whole blood
N. M. Parrish	10.1002/ibd.20799	sample other than whole blood
L. W. Duan	10.5847/WJEM.J.1920-8642.2021.01.005	sample other than whole blood
H. Duan	10.1186/s12879-020-05746-5	sample other than whole blood
D. Huang	10.1021/acs.analchem.1c04649	sample other than whole blood
E. Hong	10.1016/j.jinf.2017.10.015	sample other than whole blood
S. M. Golden	10.1016/j.diagmicrobio.2004.04.021	sample other than whole blood
J. P. Gangneux	10.3390/jof6030105	sample other than whole blood
Z. F. Fu	10.3389/fcimb.2021.745156	sample other than whole blood
W. C. Cheng	10.1038/s41467-020-18574-7	sample other than whole blood
J. Chen	10.1099/jmm.0.001259	sample other than whole blood
F. Guo	10.21037/tp-21-533	sample other than whole blood
D. He	10.2147/idr.S390256	sample other than whole blood
S. Geng	10.1016/j.ijid.2020.11.166	sample other than whole blood
H. W. Hou	10.1039/c5lc00311c	sample other than whole blood
S. Hu	10.1016/j.diagmicrobio.2018.07.011	sample other than whole blood
V. Fihman	10.1016/j.jinf.2007.09.001	sample other than whole blood
Y. Fang	10.5812/jjm.107520	sample other than whole blood
X. Y. Fang	10.1016/j.ijid.2022.07.061	sample other than whole blood
M. I. El-Amir	10.2147/idr.S213958	sample other than whole blood
E. M. Eichenberger	10.1093/cid/ciac426	sample other than whole blood
E. M. Eichenberger	10.1093/cid/ciab742	sample other than whole blood
J. D. Edgeworth	10.1086/511034	sample other than whole blood
M. Kawazu	10.1128/JCM.42.6.2733-2741.2004	sample other than whole blood
S. Hashemi Fesharaki	10.18502/cmm.4.2.63	sample other than whole blood
J. Gutierrez	10.1093/ofid/ofz090	sample other than whole blood
F. Carlesse	10.1186/s12879-016-1792-8	sample other than whole blood
P. Angelov	https://www.embase.com/search/results?subaction=viewrecord&id=L358231002&from=export	sample other than whole blood
A. Horvath	10.1186/1471-2180-13-300	sample other than whole blood
B. Hu	10.3389/fmicb.2021.641202	sample other than whole blood
J. Klepacka	10.3390/ijerph19031716	sample other than whole blood

O. Koc	10.3390/jof8090972	sample other than whole blood
L. Koh	10.1093/ajhp/zxaa030	sample other than whole blood
A. Kopf	10.3389/fmicb.2021.712775	sample other than whole blood
J. T. Kuenstner	10.3390/microorganisms8122054	sample other than whole blood
C. Andersen	10.1128/spectrum.02407-21	sample other than whole blood
P. Amornchai	10.1128/jcm.00935-07	sample other than whole blood
S. Ahmad	10.1111/myc.13022	sample other than whole blood
S. Ahmad	10.1002/jmv.23604	sample other than whole blood
M. Puig-Asensio	10.1097/ccm.0000000000000221	sample other than whole blood
A. Nicholson	https://www.mona.uwi.edu/fms/wimj/system/files/article_pdfs/dr_nicholson_special_issue_december.qxd_.pdf	sample other than whole blood
S. Narayana Iyengar	10.1021/acssensors.1c01219	sample other than whole blood
J. Xu	10.1136/jcp.55.10.774	sample other than whole blood
Y. C. Zhu	10.1128/jcm.01503-19	sample other than whole blood
Y. W. Tang	10.1016/s1473-3099(13)70364-5	sample other than whole blood
S. Taimur	10.1097/IPC.0000000000000193	sample other than whole blood
E. L. Pultorak	10.1128/JCM.00123-13	sample other than whole blood
A. Dugard	10.1016/j.jcrc.2011.11.005	sample other than whole blood
L. Derbyshire	10.1016/j.ijsu.2013.06.755	sample other than whole blood
T. Deraz	10.1016/j.ejmhg.2012.01.005	sample other than whole blood
S. O. Decker	10.3390/ijms18081796	sample other than whole blood
J. Bua	10.1371/journal.pntd.0002476	sample other than whole blood
C. A. Black	10.3389/fmicb.2020.623574	sample other than whole blood
A. Bharat	10.1126/scitranslmed.aaa8419	sample other than whole blood
J. Beuving	10.1007/s10096-014-2299-0	sample other than whole blood
M. Beganovic	10.1093/ofid/ofy350	sample other than whole blood
M. Bar-Meir	10.1097/inf.0000000000002441	sample other than whole blood
S. A. Balajee	10.1128/aac.48.4.1197-1203.2004	sample other than whole blood
F. K. Bahrani-Mougeot	10.1128/jcm.02004-07	sample other than whole blood
J. M. Janda	10.3201/eid1202.050783	sample other than whole blood
K. H. Rand	10.1006/mcpr.1994.1030	sample other than whole blood
J. Mancilla-Rojano	10.3389/fmicb.2020.576673	sample other than whole blood
N. Manz	10.1007/s00431-018-3157-3	sample other than whole blood
C. McKeating	10.1136/jclinpath-2017-204692	sample other than whole blood
R. D. Menezes	10.1590/s0036-46652015000300001	sample other than whole blood
J. Menotti	10.1016/j.jhin.2004.10.009	sample other than whole blood
E. M. Meumann	10.1128/JCM.00913-06	sample other than whole blood
Y. Midorikawa	https://www.tm.mahidol.ac.th/seameo/2016-47-5/11-67756-970.pdf	sample other than whole blood
H. Mohammed	10.26452/ijrps.v10i1.1848	sample other than whole blood
J. Mohsin	10.3390/antibiotics9100638	sample other than whole blood
M. S. Moore	10.1128/JCM.01981-13	sample other than whole blood
M. Motamedifar	10.7416/ai.2017.2154	sample other than whole blood
R. Murri	10.1007/s10096-017-3117-2	sample other than whole blood
A. Ohlin	10.1111/j.1651-2227.2008.00924.x	sample other than whole blood

U. Okomo	10.1016/S2666-5247(20)30061-6	sample other than whole blood
H. Ozbak	10.1016/j.jmoldx.2011.12.004	sample other than whole blood
S. Z. Sadrossadati	10.18502/cmm.4.2.64	sample other than whole blood
F. Safari	10.1111/myc.13504	sample other than whole blood
M. Said	10.1186/s13756-020-00778-7	sample other than whole blood
M.S. Santos	10.1590/S0100-879X2010007500065	sample other than whole blood
P. Saxenborn	10.3389/fmicb.2021.640408	sample other than whole blood
L. Selva	10.1007/s10096-011-1468-7	sample other than whole blood
E. Senneby	10.1111/j.1469-0691.2011.03609.x	sample other than whole blood
N. J. Shaly	10.3390/life12010094	sample other than whole blood
X. Y. Shi	10.1038/s41598-018-21520-9	sample other than whole blood
G. Singh	10.4103/0255-0857.154857	sample other than whole blood
W. D. Splettstoesser	10.1186/1471-2180-10-72	sample other than whole blood
L. R. Stokening	10.1016/j.jemermed.2012.02.036	sample other than whole blood
R. Subramanian	10.3390/vaccines9070756	sample other than whole blood
L. L. Wang	10.3389/fmolb.2021.659390	sample other than whole blood
M. N. van Kassel	10.1016/s2666-5247(20)30192-0	sample other than whole blood
M. A. Viviani	10.1128/JCM.44.1.218-221.2006	sample other than whole blood
J. Wan	10.3389/fcimb.2022.948602	sample other than whole blood
C. Wang	10.3389/fcimb.2022.949505	sample other than whole blood
H. Wang	10.3389/fmicb.2016.00415	sample other than whole blood
H. Y. Wang	10.1186/1476-0711-13-3	sample other than whole blood
M. Wang	10.1016/s1473-3099(21)00399-6	sample other than whole blood
W. T. Wang	10.3390/jcm11237150	sample other than whole blood
Y. Wang	10.3389/fmicb.2021.702839	sample other than whole blood
V. K. Wong	10.1371/journal.pntd.0004781	sample other than whole blood
J. B. Wood	10.1093/ofid/ofy119	sample other than whole blood
E. Iosifidis	10.1016/j.ajic.2013.02.005	sample other than whole blood
S. Sachse	10.1128/JCM.02242-08	sample other than whole blood
P. Tissori	10.7748/ns.24.26.18.s20	sample other than whole blood
S. M. Finegold	10.1128/am.18.3.458-463.1969	sample other than whole blood
S. Imbert	10.1128/jcm.01409-22	sample other than whole blood
S. N. Leaw	10.1128/jcm.44.3.693-699.2006	sample other than whole blood
K. Levi	10.1128/jcm.41.8.3890-3892.2003	sample other than whole blood
L. H. Li	10.1093/jac/dkaa432	sample other than whole blood
W. Liao	PMID: 33002409	sample other than whole blood
S. L. Lima	10.3390/jof6030110	sample other than whole blood
Q. X. Lin	10.3389/fphar.2021.716324	sample other than whole blood
H. H. Lo	10.1016/j.diagmicrobio.2014.01.027	sample other than whole blood
Y. Maaroufi	10.1128/JCM.42.7.3159-3163.2004	sample other than whole blood
Mir, L. D. N. a	10.1136/bmjopen-2012-000992	sample other than whole blood
S. Mitra	10.1093/tropej/43.3.153	sample other than whole blood
R. L. Moolenaar	10.1086/501739	sample other than whole blood
S. Shang	10.1111/j.1651-2227.2001.tb00281.x	sample other than whole blood

M. P. Weinstein	10.1128/jcm.36.7.2089-2092.1998	sample other than whole blood
M. P. Weinstein	10.1017/S0195941700055582	sample other than whole blood
N. Wellinghausen	10.1016/j.diagmicrobio.2003.11.005	sample other than whole blood
D. J. Ecker	10.1007/978-1-60327-999-4_7	sample other than whole blood
Y. Zhan	10.1021/acs.analchem.9b02030	sample other than whole blood
N. Wolff	10.1038/s41598-021-85438-5	sample other than whole blood
I. Bogdan	10.3390/medicina59071253	sample other than whole blood
R. K. Carver-Brown	10.1155/2012/424808	sample other than whole blood
M. Seki	10.2147/idr.s80123	sample other than whole blood
P. Parize	10.1016/j.cmi.2017.02.006	sample other than whole blood
D. Cai	10.1039/c4lc00669k	sample other than whole blood
S. Pahlow	10.1002/cphc.201300543	sample other than whole blood
K-H. Chen	10.1016/j.bios.2021.113740	sample other than whole blood
I-F- Cheng	10.1038/srep02365	sample other than whole blood
L. Bianchi	10.4081/mm.2015.5084	sample other than whole blood
M. M. P. Faria	10.1186/s12866-018-1211-y	sample other than whole blood
E. M. Marlowe	10.1128/jcm.41.3.1266-1269.2003	sample other than whole blood

*There could be more than one reason for exclusion, if several reasons were present, only one was selected to be included in this table.
„Non-original research article" category includes non-original articles (reviews, meta-analyses, editorials, letters, comments, notes, short reports, communications, correspondences, case reports/series, protocols, book sections, grey literature).
The "Non-commercial device" category includes assays where samples were processed using different kits from various manufacturers, but there was no single manufacturer for all the kits used.
„Not available" articles were inaccessible despite that we contacted the corresponding authors. These atudies were marked as „not retrieved" in Figure 1 in the manuscript.
